# Surface Microbiome Profiling of Dental Elevators Using Third‐Generation Sequencing: Implications for Infection Control in Dental Practice

**DOI:** 10.1002/mbo3.70178

**Published:** 2025-12-08

**Authors:** Jiajia Zheng, Kan Wang, Jinghua He, Yanchen Guan, Yuwei Wu, Jiaqi Wu

**Affiliations:** ^1^ Eighth Clinical Division (Former Employer First Clinical Division/Department of Oral and Maxillofacial Surgery) Peking University School and Hospital of Stomatology & National Center for Stomatology & National Clinical Research Center for Oral Diseases & National Engineering Research Center of Oral Biomaterials and Digital Medical Devices Beijing Shijingshan China; ^2^ Second Clinical Division Peking University School and Hospital of Stomatology & National Center for Stomatology & National Clinical Research Center for Oral Diseases & National Engineering Research Center of Oral Biomaterials and Digital Medical Devices Beijing Chaoyang China; ^3^ Department of General Dentistry Peking University School and Hospital of Stomatology & National Center for Stomatology & National Clinical Research Center for Oral Diseases & National Engineering Research Center of Oral Biomaterials and Digital Medical Devices Beijing Haidian China; ^4^ First Clinical Division Peking University School and Hospital of Stomatology & National Center for Stomatology & National Clinical Research Center for Oral Diseases & National Engineering Research Center of Oral Biomaterials and Digital Medical Devices Beijing Xicheng China

**Keywords:** 16S rRNA gene, dental elevators, microbiome, occupational exposure

## Abstract

This study aimed to characterize the biofilm‐forming microbial communities on clinically used dental elevators to assess their potential risks of cross‐contamination and nosocomial infections resulting from percutaneous injuries in dental healthcare settings. Over a period of 3 consecutive weeks starting on August 1, 2024, biofilm samples were collected from the tips of 15 dental elevators used on the first five wisdom teeth extraction patients daily. Total DNA was extracted, and specific barcoded primers were synthesized to construct SMRTbell sequencing libraries, which were subsequently sequenced using the PacBio Sequel II platform. The sequencing generated 923,990 circular consensus sequences (CCS), with an average of 61,599 CCS per sample. Taxonomic annotation revealed a diverse microbial community dominated by genera such as *Prevotella*, *Fusobacterium*, *Streptococcus*, and *Lactobacillus*, alongside unclassified taxa from the *Candidatus Saccharibacteria* (TM7) group. Alpha and beta diversity analyses demonstrated significant variations in microbial composition across samples, highlighting the heterogeneity of biofilm formation, while strong positive correlations observed between specific bacterial genera, such as *Bacillus* and *Paenibacillus*, suggested potential co‐colonization patterns. These findings underscore the complexity of microbial contamination on dental instruments and emphasize the need for improved sterilization protocols to mitigate infection risks. Consequently, this study provides valuable insights into the microbiological safety of dental practices and highlights the utility of third‐generation sequencing in advancing infection control strategies.

## Graphical abstract

This study utilized PacBio Sequel II sequencing to characterize biofilm‐forming microbial communities on used dental elevators. Analysis of 923,990 CCS sequences revealed diverse contamination with clinically relevant genera including Prevotella, Fusobacterium, and Streptococcus. Findings emphasize enhanced sterilization protocols and demonstrate third‐generation sequencing's value for dental infection control strategies. The image was modified using Doubao‐Seedream‐4.0.

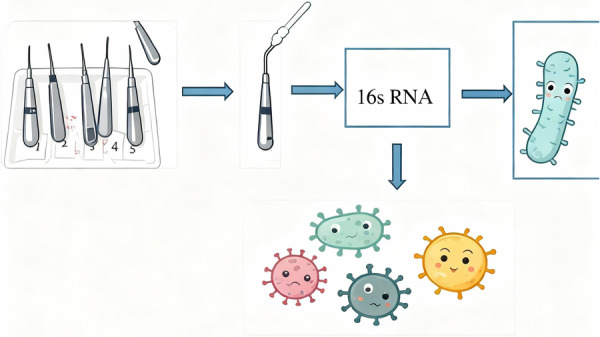

## 1 Introduction

Dental elevators, used for tooth extraction (Bussell and Graham 2008), are exposed to various oral microorganisms, including bacteria, fungi, and viruses, which can form biofilms on their surfaces. Dental personnel face significant risks of occupational exposure injuries during their professional duties due to contact with sharp instruments, infectious materials, or other hazards (Utkarsha Lokesh et al. 2014). These injuries, which can lead to infection spread and long‐term disabilities, are often caused by the mishandling of sharp‐tipped instruments like dental elevators, commonly used in oral surgery. Effective infection control in dental settings requires thorough analysis of microbial contamination on dental elevators, which are frequently exposed to blood, saliva, and tissue debris that may harbor pathogens. Globally, hospital‐associated infections (HAIs) represent a significant and ongoing challenge for healthcare (Chen et al. 2017a). Bacterial analysis of surface contaminants is essential for assessing contamination levels and guiding the implementation of appropriate disinfection protocols (Klassert et al. 2022). Handling contaminated medical instruments before disinfection, especially during transport, poses a high risk of needlestick injuries to healthcare workers (Ippoliti et al. 2025). These injuries are worsened by bacterial growth on instruments, which forms biofilms—structured bacterial communities protected by a slimy matrix (Subramani et al. 2025). Biofilms increase infection risk after injury by helping bacteria survive, resist disinfection, and spread into wounds (Azeredo et al. 2017). Strong infection control practices and regular microbial testing are critical to reduce these risks and prevent healthcare‐associated infections.

This study aims to employ third‐generation 16S rRNA sequencing technology to investigate surface contamination on dental elevators after clinical use. By precisely identifying the microbial communities present on these instruments, the study seeks to provide detailed insights into the composition and diversity of biofilm‐forming microorganisms.

## 2 Materials and Methods

### 2.1 Sample Collection

Starting on August 1, 2024, for a period of 3 consecutive weeks, used dental elevators were collected daily. Following the treatment of each of the first five wisdom teeth extraction patients, the used elevator was placed in the regular instrument waste container in the treatment room. The researchers were entirely uninformed about any patient demographic details or medical records. After the fifth patient, one of the five used elevators was randomly selected, and the tip of this selected elevator was swabbed using a sterile clean swab（1 sample/1 workday). The swab was then immediately stored at −80°C. The time that the elevators remained in the waste container was always less than 3 h (the clinic's disinfection interval). Use a standard single‐headed medical cotton swab to wipe the surface of the contaminated dental extraction instruments. Wipe the surface with moderate pressure for 10–15 s, then place the swab into a 10 mL EP tube. Take two swabs from each object surface.

### 2.2 Library Construction and Sequencing

Total DNA was extracted and amplified using barcoded full‐length 16S rRNA gene primers. PCR products were purified, quantified, and used to construct SMRTbell libraries for sequencing on the PacBio Sequel II platform. Raw sequencing data (BAM format) were processed using SMRTlink to generate circular consensus sequencing (CCS) reads, which underwent three sequential quality control steps: (1) barcode‐based demultiplexing using lima v1.7.0, (2) primer removal and length filtering using cutadapt v1.9.1, and (3) chimera detection and removal using UCHIME v4.2. High‐quality effective CCS sequences were retained for downstream taxonomic and functional analyses.

These Effective‐CCS sequences were clustered into operational taxonomic units (OTUs) using Usearch with a 97% similarity threshold. Feature sequences were then taxonomically annotated using a naive Bayes classifier, referencing the SILVA database. Both alpha and beta diversity analyses were performed. Finally, functional gene prediction was performed using PICRUSt2, with annotation against the KEGG and COG databases.

## 3 Results

This study employed third‐generation sequencing (PacBio platform) to perform a comprehensive analysis of microbial diversity in 15 dental elevator biofilm samples. Sequencing of 15 samples yielded 923,990 circular consensus sequences (CCS), with an average of 61,599 CCS sequences per sample. The sequencing data evaluation results for each sample are presented in Table 1, where: Sample ID represents the sample name; Raw‐CCS indicates the number of CCS reads identified for each sample; Clean‐CCS represents the number of sequences after barcode identification and primer removal; Effective‐CCS denotes the number of sequences retained after length filtering and chimera removal for downstream analysis; AvgLen (bp) indicates the average sequence length for each sample; and Effective (%) represents the percentage of Effective‐CCS reads relative to Raw‐CCS reads.

**Table 1:** Sequencing data quality assessment for each sample.

Sample ID	Raw CCS	Clean CCS	Effective	AvgLen(bp)	Effective(%)
			CCS		
M01	65,868	65,847	62,751	1,454	95.27
M02	54,330	54,307	52,721	1,457	97.04
M04	68,191	68,181	66,533	1,440	97.57
M05	61,555	61,527	59,604	1,462	96.83
M06	56,143	56,069	54,459	1,453	97.0
M07	57,984	57,940	51,874	1,453	89.46
M08	55,024	54,954	50,548	1,451	91.87
M09	66,281	66,262	63,470	1,453	95.76
M10	59,229	59,201	58,224	1,457	98.3
M12	63,419	63,409	61,378	1,445	96.78
M13	68,661	68,646	68,231	1,468	99.37
M14	63,347	63,298	61,873	1,445	97.67
M15	63,751	63,741	62,688	1,463	98.33
M16	61,664	61,545	61,375	1,451	99.53
M17	58,543	58,535	57,081	1,440	97.5

**Figure 1:**
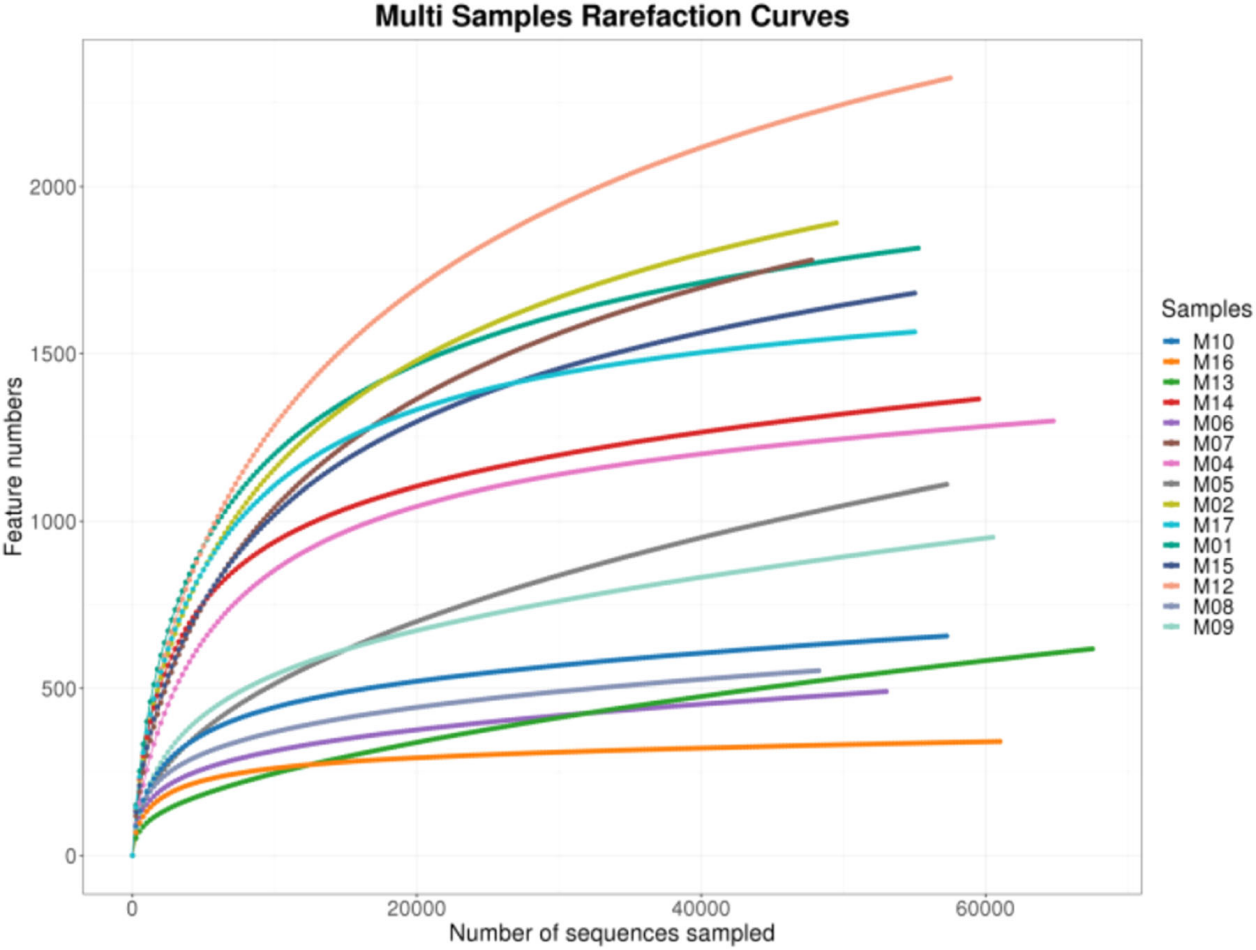
Rarefaction curves of all samples.

**Figure 2:**
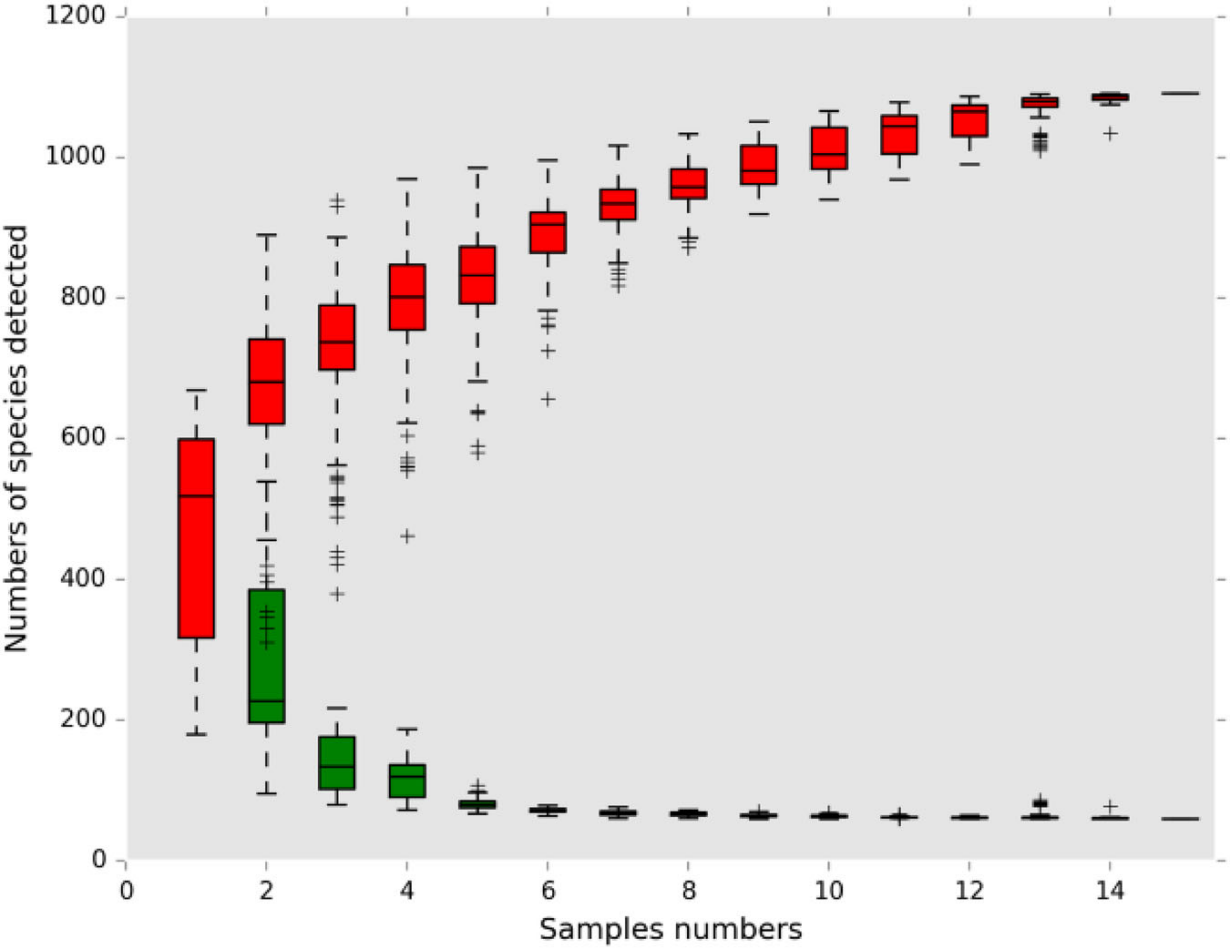
genus‐level species accumulation curves.

Analyses included OTU determination, taxonomic annotation, alpha diversity assessment, beta diversity assessment, differential abundance analysis, correlation analysis, and functional prediction. Rarefaction curves were generated to determine if the sequencing depth was sufficient to capture the microbial diversity of each sample. The resulting sample rarefaction curve is illustrated in Figure 1, and the genus‐level species accumulation curves are shown in Figure 2.

**Figure 3:**
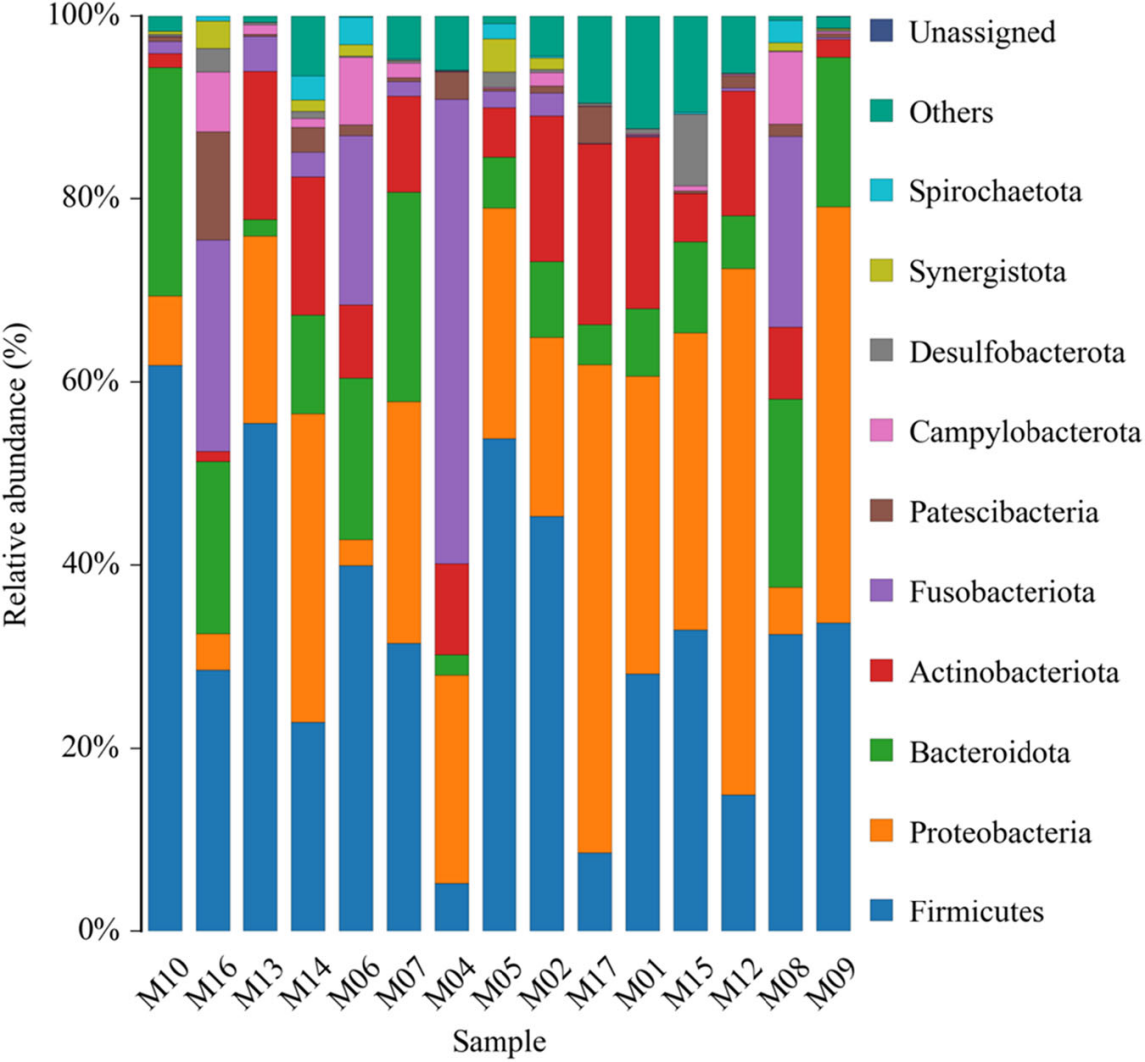
Relative abundance of different bacterial phyla in 15 samples.

The phyla distribution comparing is shown in Figure 3.

The top 10 phyla identified were: *Spirochaetota, Synergistota, Desulfobacterota, Campylobacterota, Patescibacteria, Fusobacteriota, Actinobacteriota, Bacteroidota, Proteobacteria*, and *Firmicutes*.

**Figure 4:**
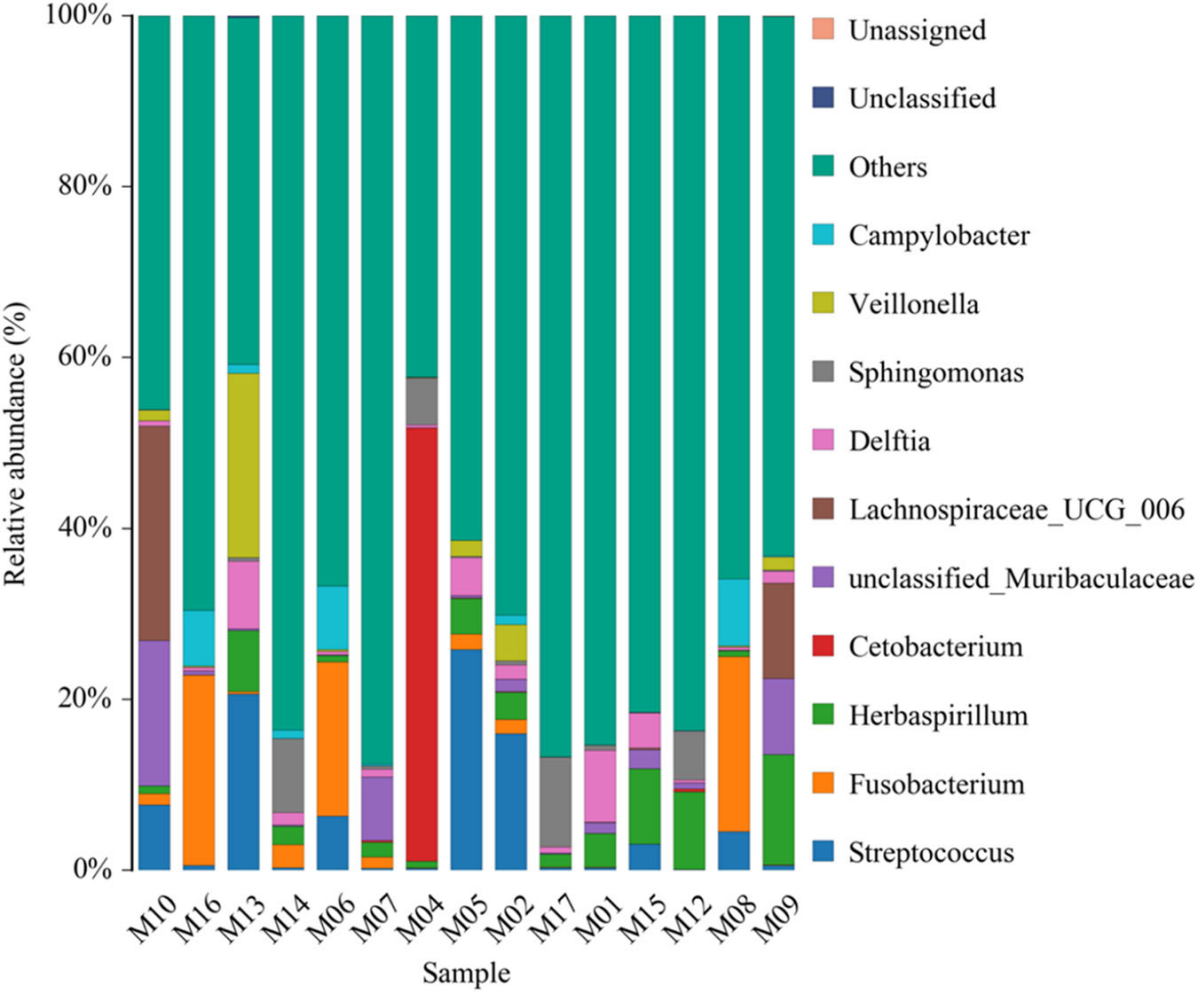
Relative abundance of different bacterial genus in 15 samples.

The genus distribution comparing is shown in Figure 4.

The top 10 genera identified were: *Campylobacter, Veillonella, Sphingomonas, Delfia, Lachnospiraceae_UCG_006, unclassified_Muribaculaceae, Cetobacterium, Herbaspirillum, Fusobacterium, and Streptococcus*.

**Figure 5:**
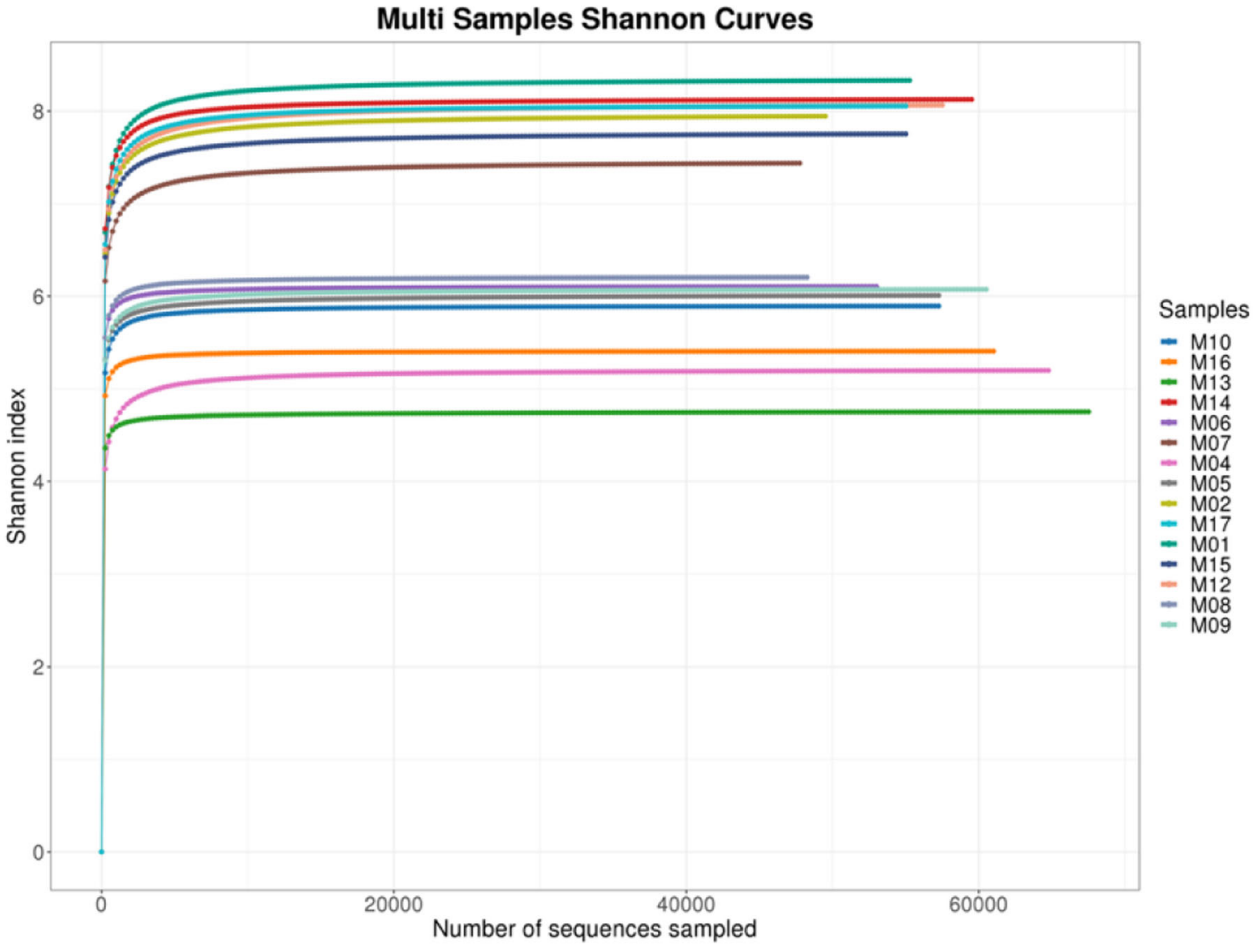
Rarefaction curves of Shannon diversity indices for microbial communities on dental elevators post‐use.

The rarefaction analysis revealed that microbial diversity on used dental elevators, as measured by the Shannon index, increased with sequencing depth and stabilized at approximately 40,000 sequences (Figure 5). The maximum Shannon index reached ~6, indicating high microbial diversity. This plateau suggests that the sequencing effort sufficiently captured the taxonomic richness and evenness of the microbial communities present on the instruments.

**Figure 6:**
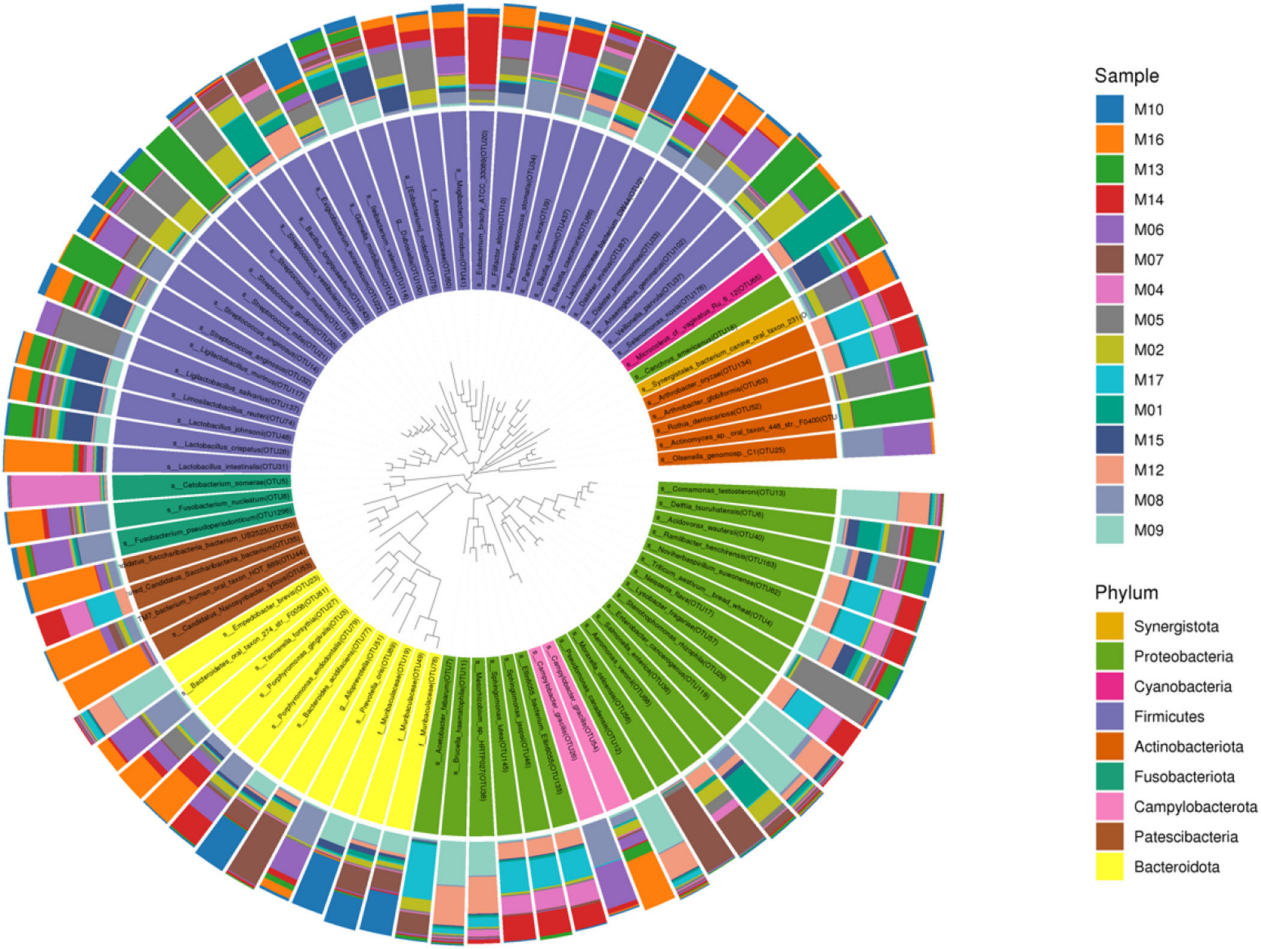
Phylum‐level taxonomic composition of microbial communities on dental elevators post‐use.

Figure 6 summarized the distribution of bacterial phyla across 15 samples (M01–M17). Major phyla include *Proteobacteria, Firmicutes, Actinobacteriota, Fusobacteriota, and Campylobacteriota., alongside less abundant groups such as Synergistota, Cyanobacteria, and Patescibacteria*. The visualization highlights variations in phylum dominance across samples, reflecting differences in microbial community structure.

**Figure 7:**
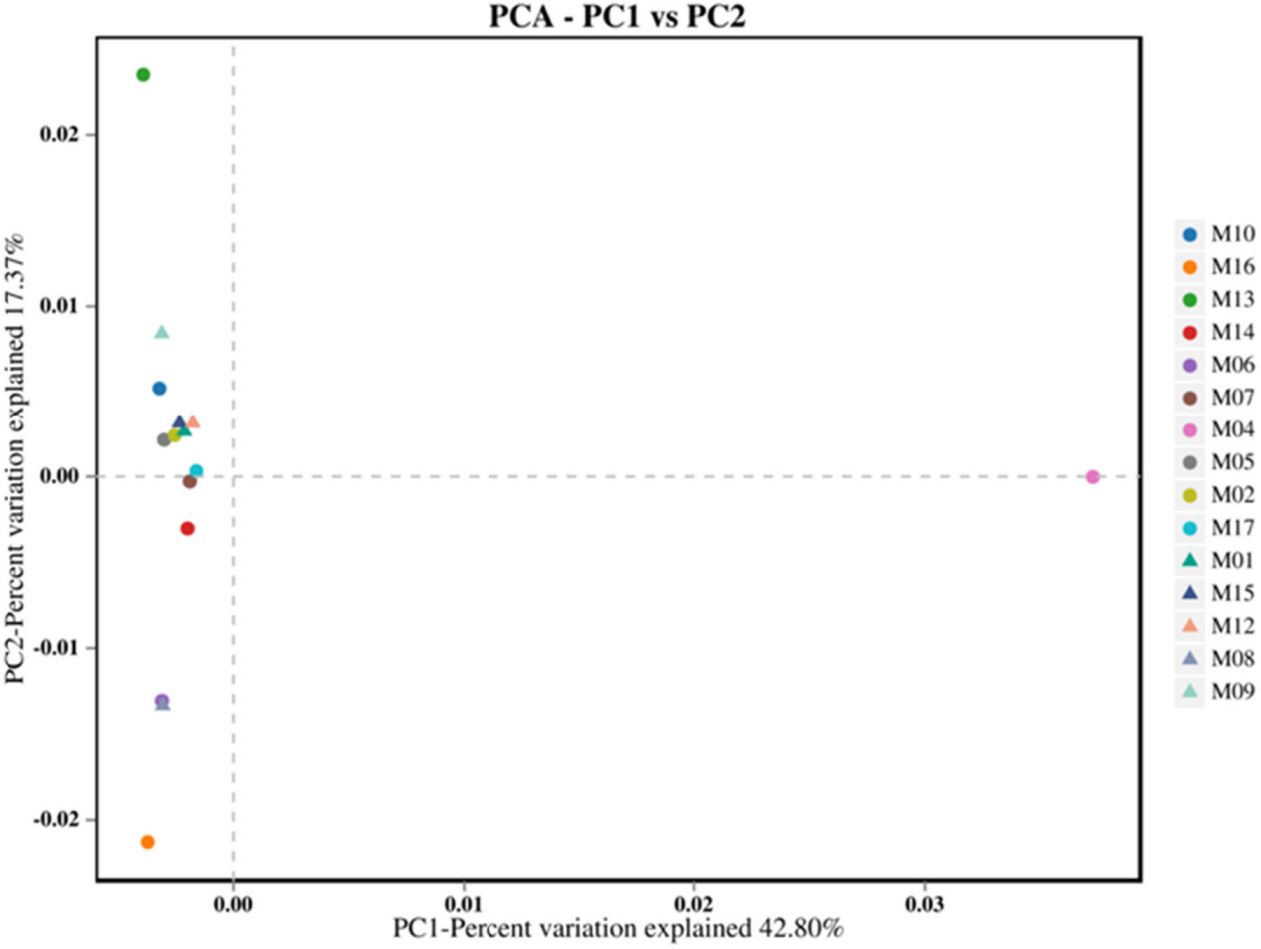
Principal component analysis (PCA) of microbial communities associated with dental elevators post‐use.

PCA revealed distinct patterns in microbial community composition across dental elevators (Figure 7). PC1 accounted for 42.80% of the variance, indicating it captured the dominant source of variation among samples. PC2 explained an additional 17.37% of variance. The distribution of samples along PC1 (range: 0.00–0.03) and PC2 (range: ‐0.02 to 0.01) suggests variability in microbial profiles, with no overt clustering of samples into discrete groups. This implies continuous, rather than categorical, differences in community structure.

**Figure 8:**
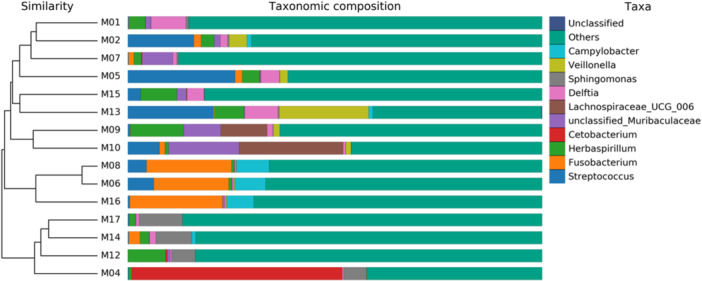
Taxonomic similarity and composition of microbial communities on dental elevators post‐use.

The taxa include both commensal and pathogenic genera (e.g., *Streptococcus, Fusobacterium, Campylobacter*), as well as unclassified groups, which highlight unresolved or novel microbial diversity, as shown in Figure 8. In addition to these identified genera, the figure also highlights a substantial fraction of unclassified groups. The presence of these unclassified taxa suggests the existence of unresolved or potentially novel microbial diversity within the sampled environment.

**Figure 9:**
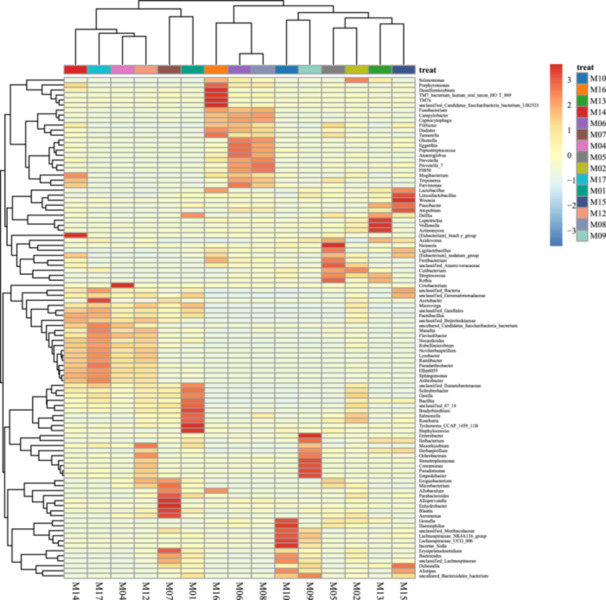
Heatmap displaying the relative abundance of microbial taxa across samples.

The Figure 9 illustrates the taxonomic profiles of 15 samples (M01–M17), highlighting the relative abundance of bacterial genera and unclassified taxa.

**Figure 10:**
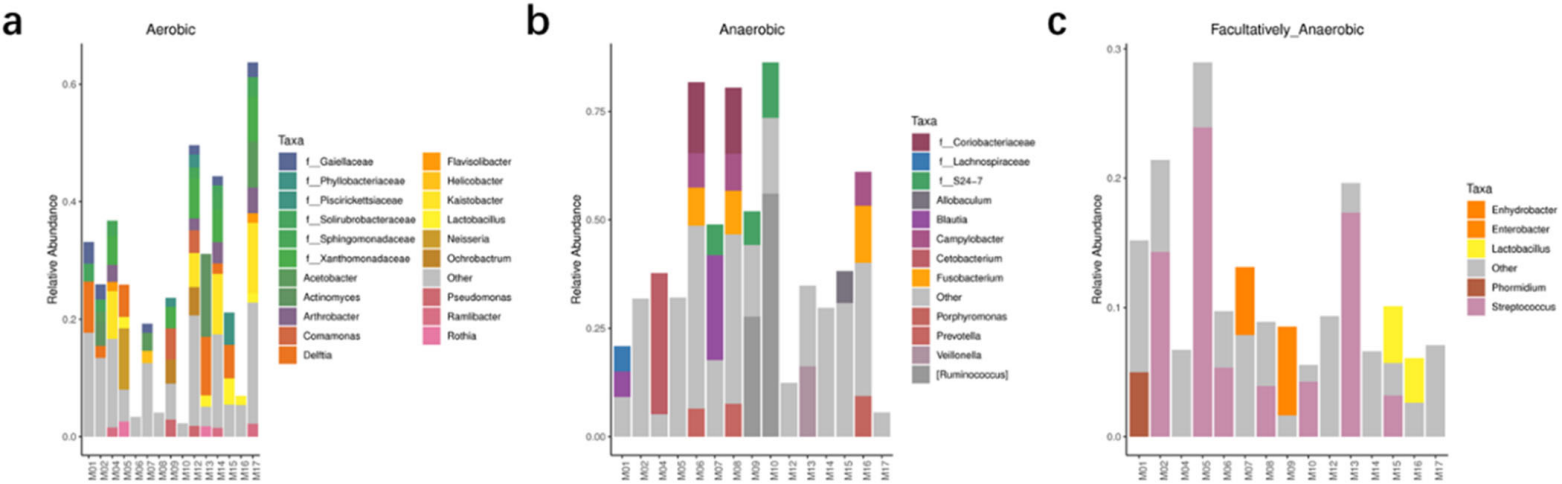
The relative abundance of bacterial taxa across different samples. Samples are grouped based on their oxygen requirements: (a) aerobic, (b) anaerobic, and (c) facultatively anaerobic.

The main groups of aerobic bacteria identified were: *Actinomyces, Pseudomonas, Lactobacillus, Neisseria, and Helicobacter*. The main groups of anaerobic bacteria identified were *Fusobacterium, Prevotella, Porphyromonas, Veillonella*, and *Campylobacter*. The main groups of facultatively anaerobic bacteria identified were *Campylobacter, Streptococcus, Prevotella, Porphyromonas, and Veillonella* (Figure 10).

**Figure 11:**
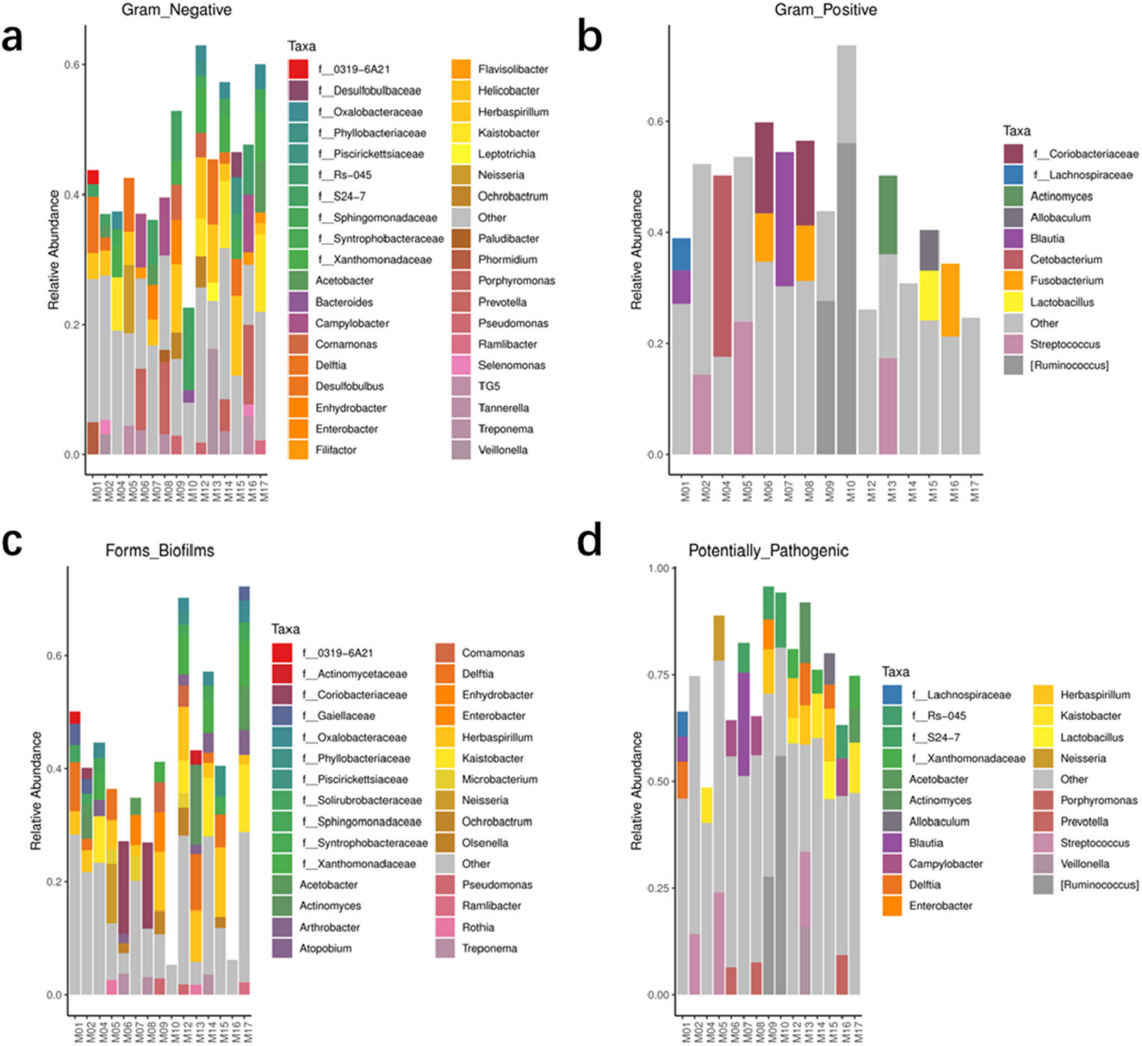
Taxonomic diversity and relative abundance profiles across experimental samples. (a) Gram‐negative genera, (b) Gram‐positive genera, (c) biofilm‐forming genera, (d) potentially pathogenic bacteria.

Figure 11 illustrates the relative abundance of various bacterial taxa identified on dental elevator surfaces. This figure highlights several Gram‐negative genera, including *Pseudomonas, Prevotella, Porphyromonas, Campylobacter, Neisseria*, and *Veillonella*, as well as Gram‐positive genera such as *Streptococcus, Lactobacillus*, and *Actinomyces*. Additionally, biofilm‐forming genera like *Pseudomonas, Actinomyces, Neisseria, Enterobacter*, and *Treponema* are noted. This figure emphasizes the presence of potentially pathogenic bacteria, including *Campylobacter, Streptococcus, Prevotella, Porphyromonas*, and *Veillonella*, which pose a risk of infection to both patients and healthcare workers.

**Figure 12:**
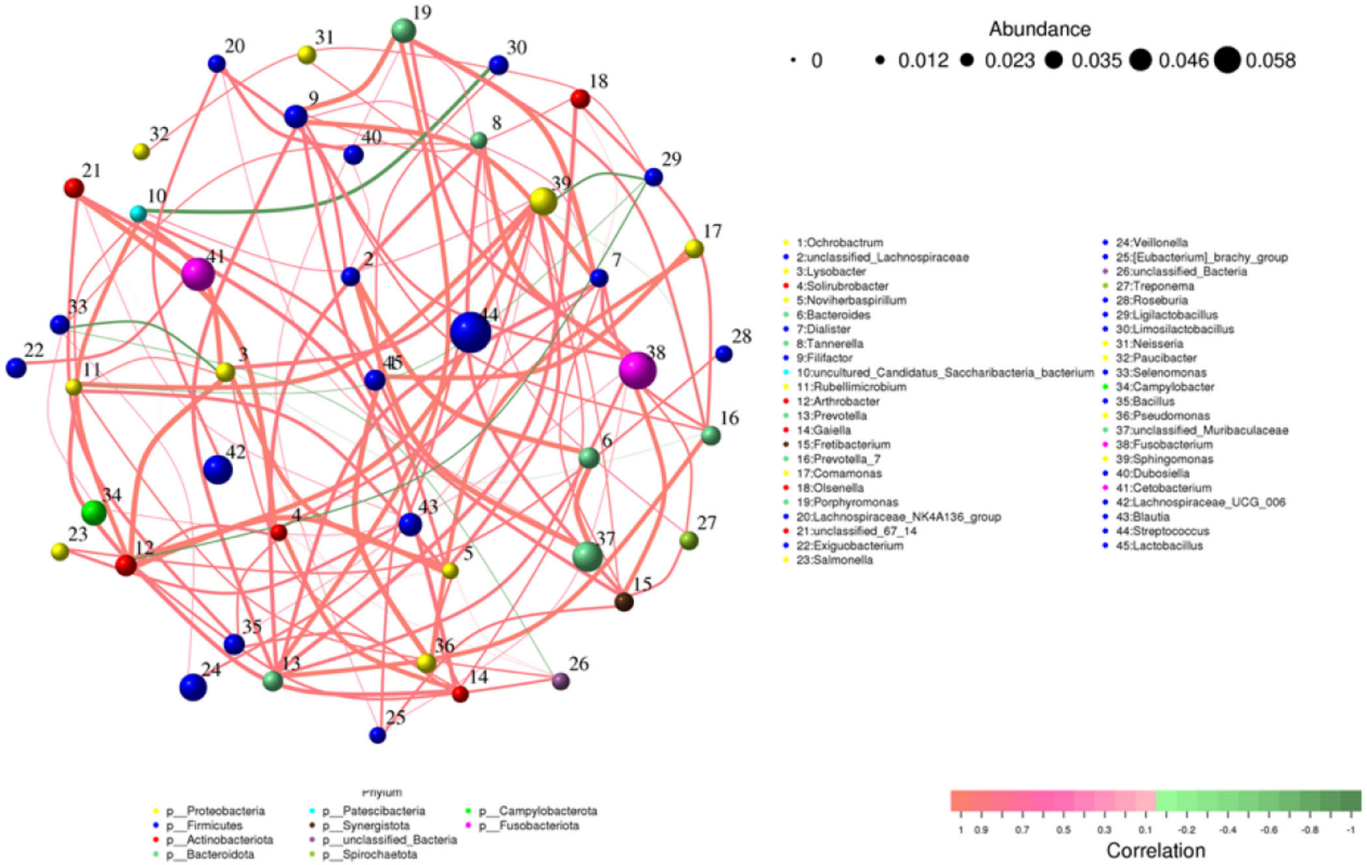
Correlation network analysis elucidated co‐occurrence relationships between microbial taxa. The statistical analysis of node and edge attributes within the network graph indicates that certain taxa exhibit important ecological functions within the microbial community.

Network diagrams (Figure 12) serve as a visual representation of correlation analysis. These networks were constructed by performing Spearman's rank correlation analysis on the abundance and variation of each species across the samples. Data were filtered to include only correlations greater than 0.1 with p‐values less than 0.05.

Strong positive correlations were found between the following bacterial genera pairs: Node 35 (*Bacillus*) and Node 36 (*Paenibacillus*), Node 38 (*Staphylococcus*) and Node 39 (*Streptococcus*), Node 40 (Dubosiella) and Node 41 (Alistipes)—suggest potential co‐colonization patterns and synergistic interactions within the microbial communities on dental elevators.

**Figure 13:**
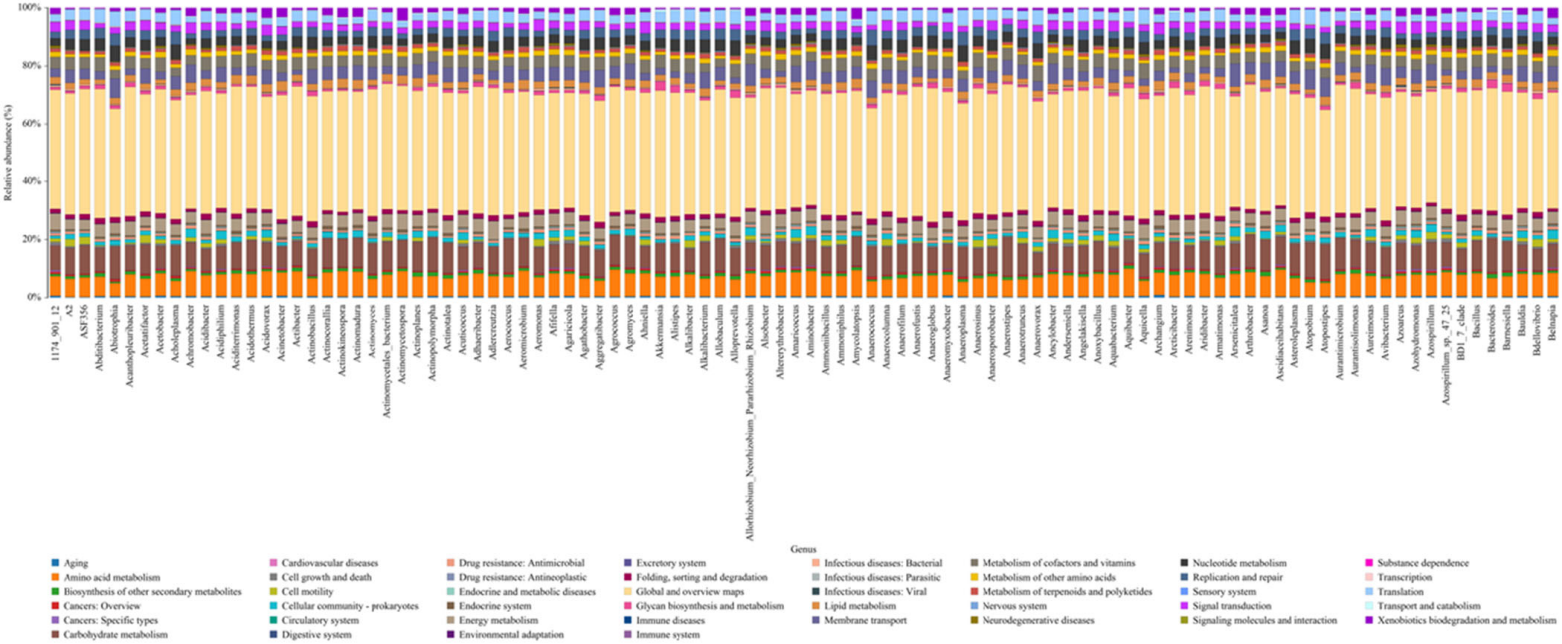
KEGG pathway analysis.

Figure 13 illustrates the distribution of KEGG pathway identifiers (ranging from 1.2 to 1.501) identified in microbial communities on dental elevators post‐use. Each identifier corresponds to specific metabolic, genetic, or disease‐associated pathways. The visualization highlights the functional diversity and potential of the microbiota, with a particular emphasis on pathways related to microbial survival, virulence, and antibiotic resistance.

**Figure 14:**
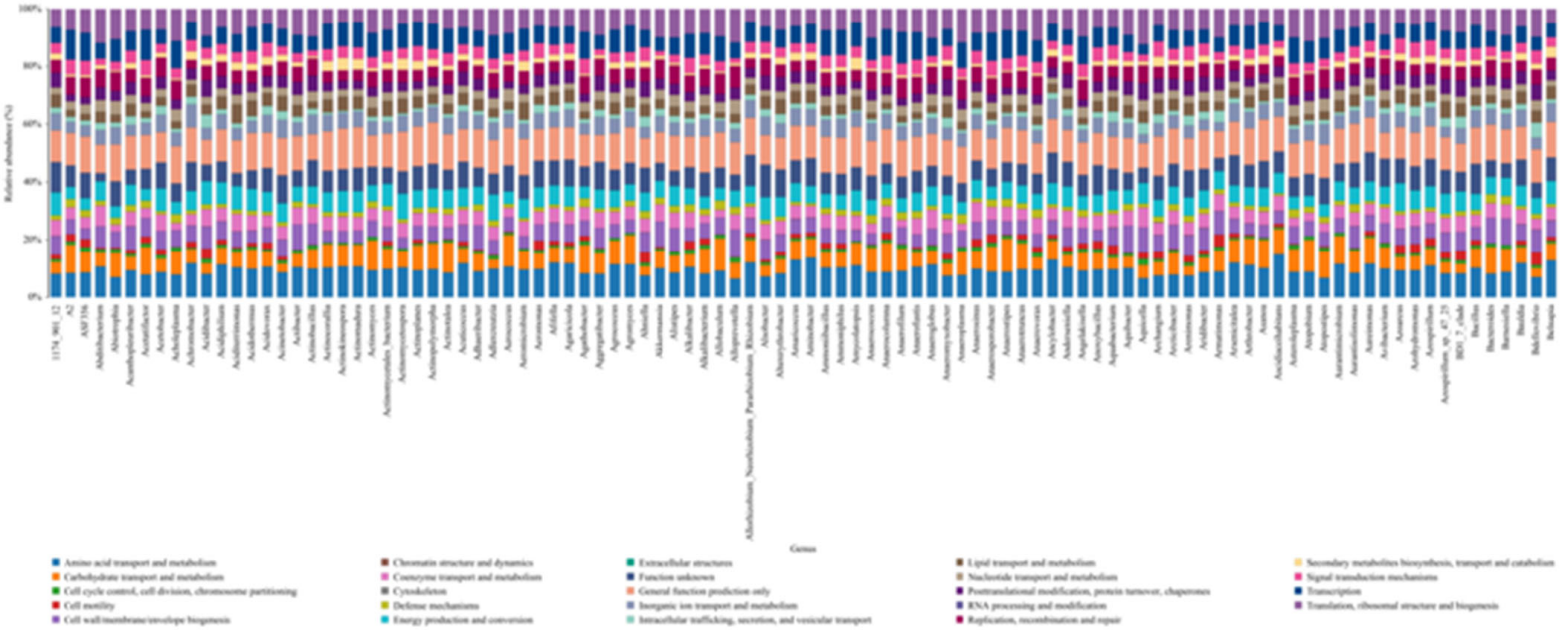
Functional classification of microbial communities on dental elevators post‐use, based on Clusters of Orthologous Groups (COG) categories.

Figure 14 of the COG categorizes microbial genes into five major functional groups: (1) amino acid and carbohydrate metabolism; (2) cellular structure and energy processes; (3) transport and stress resistance; (4) lipid metabolism and biofilm dynamics; and (5) genetic and biosynthetic functions. Within these groups, subcategories further delineate roles in nutrient acquisition, cellular maintenance, resistance mechanisms, biofilm persistence, and genetic regulation.

## 4 Discussion

In dental healthcare, sharp injuries from contaminated instruments like extraction forceps, dental elevators, needles, and blades pose a significant occupational hazard, especially for oral and maxillofacial surgery staff (Jakubovics et al. 2014). These injuries increase the risk of nosocomial infections, threatening staff safety. Dental elevators, frequently used in oral surgery, are particularly prone to contamination by oral bacteria, raising the risk of postoperative infections. Nosocomial pathogens include bacteria, fungi, and viruses, with bacteria being the most common. Identifying the types and levels of bacteria on dental instruments is crucial for improving sterilization and disinfection protocols.

Traditional bacterial culture methods have limitations in detecting the full range of pathogens. However, third‐generation 16S rRNA sequencing technology offers a powerful solution, providing comprehensive insights into the composition of microbial communities on surfaces, including those that are difficult to culture (Regueira‐Iglesias et al. 2023). This technology's increasing use across various fields underscores its value in identifying contamination sources, particularly in healthcare (Vieites et al. 2010).

*Firmicutes*, a large and diverse phylum characterized by a high proportion of Gram‐positive bacteria, were ubiquitously detected in all samples analyzed from the surface of stainless‐steel instruments (Megrian et al. 2020). This phylum exhibits a cosmopolitan distribution, with members found in diverse habitats, including soil, aquatic environments, within animals and plants, and the human gastrointestinal tract (Sun et al. 2023).

*Firmicutes*, a major phylum of Gram‐positive bacteria, play a significant role in oral health and disease. This group includes genera such as *Streptococcus*, *Lactobacillus*, and *Staphylococcus*, which are commonly found in the oral cavity (Peng et al. 2023). While some *Firmicute*s are commensal, others are pathogenic and contribute to oral infections. For instance, *Staphylococcus aureus* can cause localized infections (Baranova et al. 2024), particularly in immunocompromised individuals or following dental procedures. In conditions like pericoronitis, *Firmicutes* can exacerbate inflammation and tissue damage (Mansfield et al. 2012).

Clinically used dental elevators harbor complex microbial communities, dominated b*y Prevotella, Fusobacterium*, and *Streptococcus*. These bacteria, critical in biofilm formation, also contribute to periodontal diseases and postoperative infections, posing a significant occupational risk to sterilization staff. *Prevotella* and *Fusobacterium*, anaerobic pathogens, cause severe infections, especially in immunocompromised individuals (Thol et al. 2024). *Streptococcus*, while often commensal, includes pathogenic species that can cause endocarditis or abscesses from accidental injuries (Mada et al. 2024). The presence of these pathogens on dental elevators not only increases the risk of sharps injuries, common in sterilization settings, and subsequent infections, but also significantly elevates the risk of exposure for staff during the handling and sorting of contaminated instruments (Upendran et al. 2023).

The study identified strong positive correlations between *Bacillus* and *Paenibacillus*, spore‐forming bacteria that likely cooperate to enhance biofilm stability (Bartula et al. 2023). This makes the biofilms more resistant to sterilization. The correlation between *Staphylococcus* and *Streptococcus*, common pathogens, indicates a synergistic relationship that increases biofilm complexity. *Staphylococcus aureus* is a particular concern due to its potential to cause severe infections (Lister and Horswill 2014). Additionally, *Dubosiella* and *Alistipes*, anaerobic bacteria, show a strong positive correlation, suggesting a symbiotic relationship that may contribute to the persistence of anaerobic pathogens. The presence of potentially pathogenic genera, such as *Streptococcus and Campylobacter*, highlights the risk of cross‐contamination and nosocomial infections in dental healthcare settings.

The detection of unclassified taxa, such as *Candidatus Saccharibacteria* (TM7), further underscores the complexity of biofilm communities and the limitations of current disinfection methods (Naud et al. 2023). TM7, a poorly characterized candidate phylum, is thought to have a parasitic relationship with other bacteria, potentially enhancing biofilm stability and resistance to disinfection (Kharitonova et al. 2021). The presence of these bacteria on dental elevators contributes to biofilm persistence and increases infection risks, emphasizing the need for targeted sterilization strategies that disrupt microbial interactions and biofilm matrices, rather than just reducing microbial biomass. These bacteria, which are thought to have a parasitic relationship with other bacteria (Bor et al. 2018), may enhance biofilm stability and resistance to disinfection. This parasitic relationship could contribute to the persistence of biofilms on dental elevators.

KEGG pathway analysis revealed a diverse range of functional pathways in the microbial communities on dental elevators, including those associated with survival, virulence, and antibiotic resistance (Chen et al. 2017b). Pathways related to biofilm formation, adhesion proteins, and efflux pumps likely explain the ability of these microbes to colonize instruments and resist disinfection. Figure 13 provides a comprehensive overview of this functional potential. The presence of pathways for nutrient acquisition, stress response, and biofilm formation allows these communities to persist despite disinfection. Virulence pathways, including adhesion and toxin production, indicate the potential for pathogenicity. The identification of antibiotic resistance pathways, such as efflux pumps and beta‐lactamase production, raises concerns about occupational exposure hazards. The high number of identified pathways (up to 1.501) reinforces the complexity of these microbial ecosystems and the need for effective sterilization to prevent cross‐contamination.

COG functional classification revealed the metabolic versatility of microbial communities on dental elevators, with genes involved in nutrient acquisition, stress response, and biofilm dynamics. The classification categorized genes into five major functional groups, providing insights into their adaptive strategies. Specifically, pathways for amino acid and carbohydrate metabolism, cell cycle control, cell wall biogenesis, energy production, and inorganic ion transport are crucial for their survival (Dai 2011). Pathways for secondary metabolite production and signal transduction enhance their resilience and biofilm formation (Bridges et al. 2022). This functional diversity underscores the challenge of eradicating these microbes. The high microbial diversity, possibly due to direct interaction with oral tissues, and the strong correlations between specific bacterial pairs highlight the need for advanced sterilization strategies to effectively disrupt these communities

The clinical implications of these findings are considerable, as the persistence of biofilms on dental instruments significantly increases the risk of cross‐contamination and nosocomial infections. The observed heterogeneity in microbial communities across samples suggests that factors such as patient oral microbiota, procedural conditions, and instrument handling practices play a crucial role in biofilm formation. These findings collectively highlight the significant occupational health risk for dental staff, who face exposure to potentially pathogenic bacteria through contaminated dental elevators. Consequently, regular monitoring of microbial communities on dental instruments is essential to assess the effectiveness of sterilization protocols and identify areas for improvement.

Future studies should integrate transcriptomic or proteomic data to prioritize pathways with clinical relevance. Furthermore, the biofilm‐forming nature of these bacteria enhances their resistance to disinfection, making them more likely to persist on instruments before initial cleaning. This increases the risk of exposure for staff during the handling and sorting of contaminated instruments. The study underscores the need for stringent protective measures, such as the use of puncture‐resistant gloves, proper handling protocols, and immediate access to post‐exposure prophylaxis, to mitigate these risks (Mahasneh et al. 2020). Enhanced training and awareness about the potential hazards of handling biofilm‐contaminated instruments are also essential to ensure the safety of sterilization room personnel.

## 5 Conclusion

Biofilms on dental instruments like elevators represent a significant risk for occupational hazards. Addressing biofilm‐related risks requires a combination of advanced cleaning technologies, rigorous monitoring, and ongoing education for healthcare professionals.

## Author Contributions

**Jiajia Zheng:** formal analysis, methodology, writing – original draft. **Kan Wang:** methodology, writing – original draft, validation. **Jinghua He:** validation, methodology. **Yanchen Guan:** validation, software, data curation. **Yuwei Wu:** writing – review and editing, project administration, resources. **Jiaqi Wu:** funding acquisition, conceptualization, writing – review and editing, project administration.

## Ethics Statement

The authors have nothing to report.

## Conflicts of Interest

The authors declare no conflicts of interest.

## Supplementary Material

Refer to Web version on PubMed Central for supporting material.

## Supporting information

customer_backup.
