# Supplementary figures and images for: Surface Microbiome Profiling of Dental Elevators Using Third‐Generation Sequencing: Implications for Infection Control in Dental Practice
Source: Microbiologyopen. 2025 Dec 8;14(6):e70178. doi: 10.1002/mbo3.70178 (PMC12685768; doi:10.1002/mbo3.70178)

Relative abundance

$10^{-1}$

$10^{-2}$

$10^{-3}$

$10^{-4}$

$10^{-5}$

0

500

1000

1500

2000

Species rank

M10  
M16  
M13  
M14  
M06  
M07  
M04  
M05  
M02  
M17  
M01  
M15  
M12  
M08  
M09

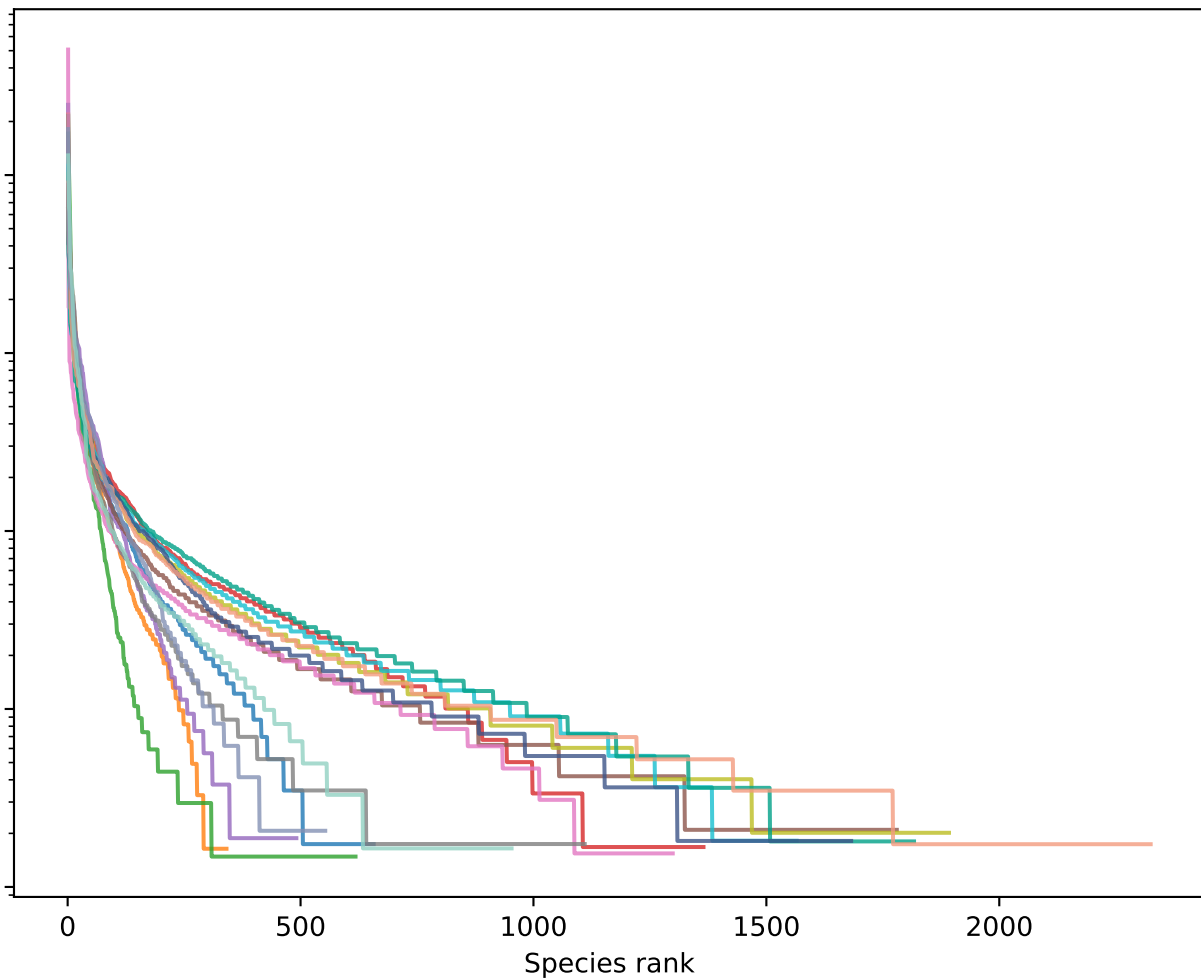

Supplement: Supplementary file 1 — customer_backup. [file MBO3-14-e70178-s001.zip › customer_backup/customer_backup/alpha_diversity/rank_abund_curve/allsample/allsample.rank.abund.curve.pdf]

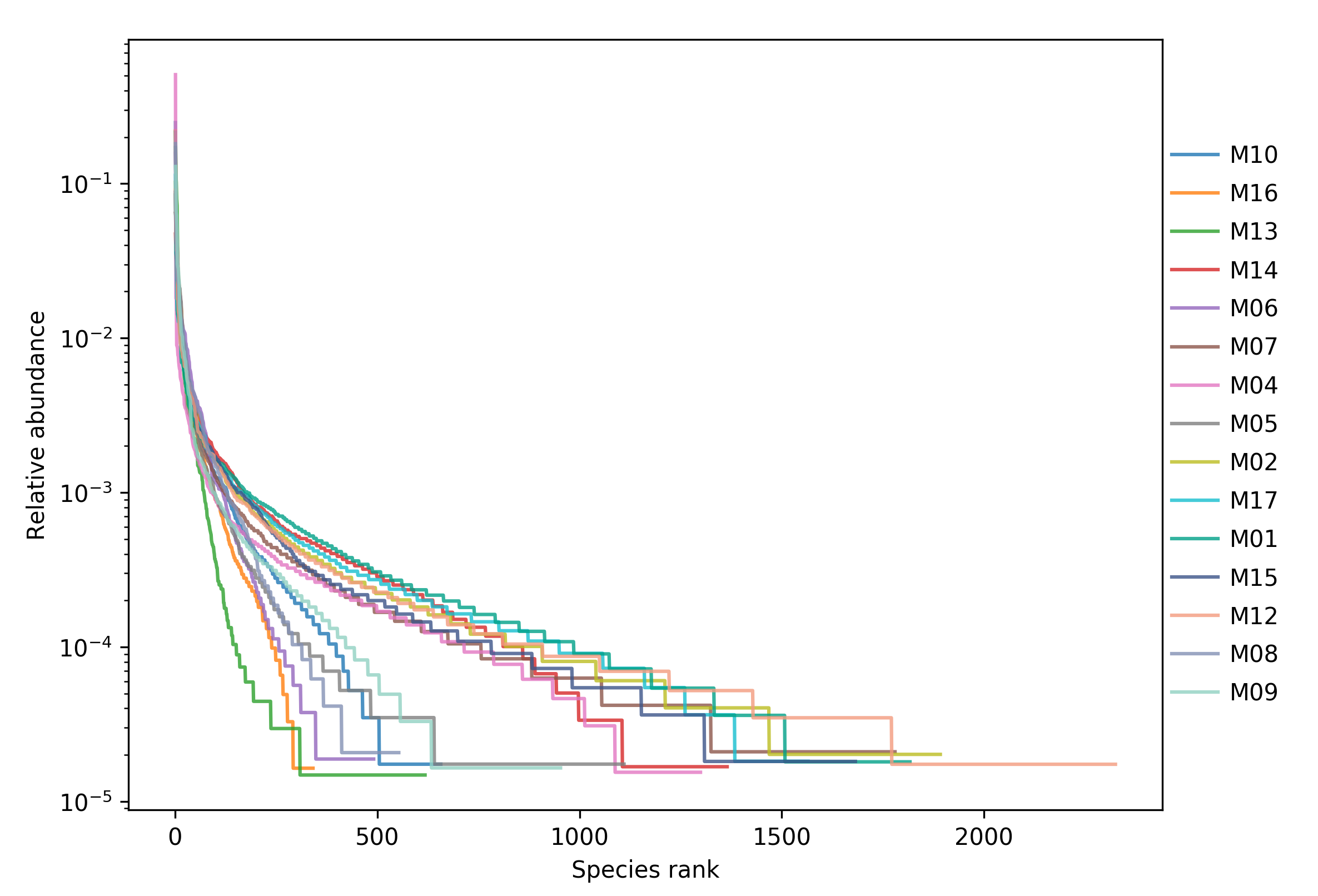

Supplement: Supplementary file 1 — customer_backup. [file MBO3-14-e70178-s001.zip › customer_backup/customer_backup/alpha_diversity/rank_abund_curve/allsample/allsample.rank.abund.curve.png]

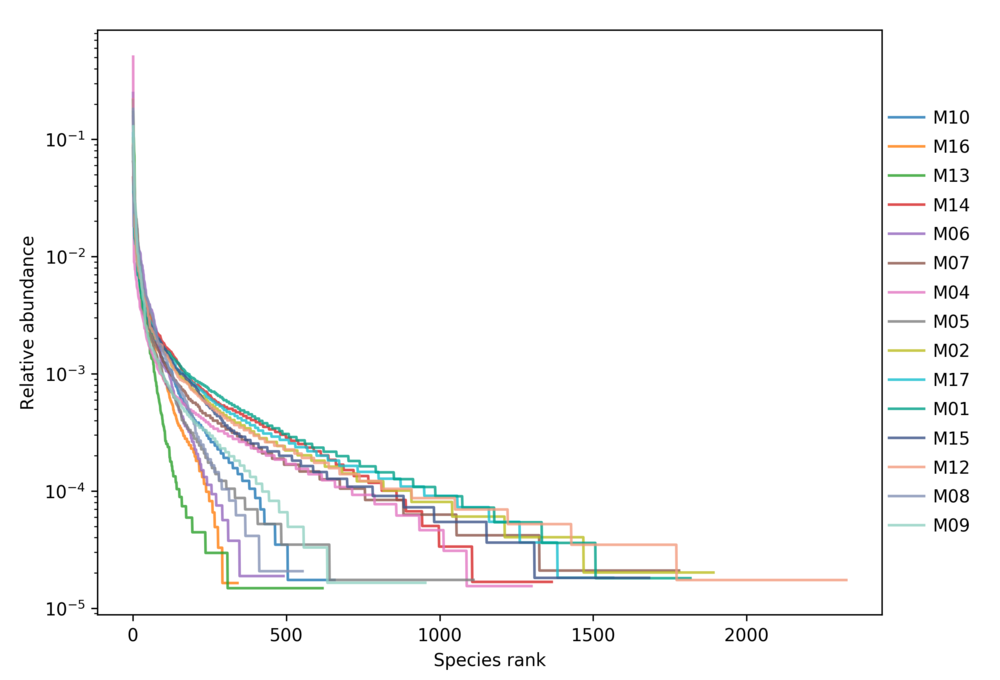

Supplement: Supplementary file 1 — customer_backup. [file MBO3-14-e70178-s001.zip › customer_backup/customer_backup/alpha_diversity/rank_abund_curve/allsample/allsample.rank.abund.curve_small.png]

Relative abundance

$10^{-1}$

$10^{-2}$

$10^{-3}$

$10^{-4}$

$10^{-5}$

0

500

1000

1500

2000

Species rank

M10  
M16  
M13  
M14  
M06  
M07  
M04  
M05  
M02  
M17  
M01  
M15  
M12  
M08  
M09

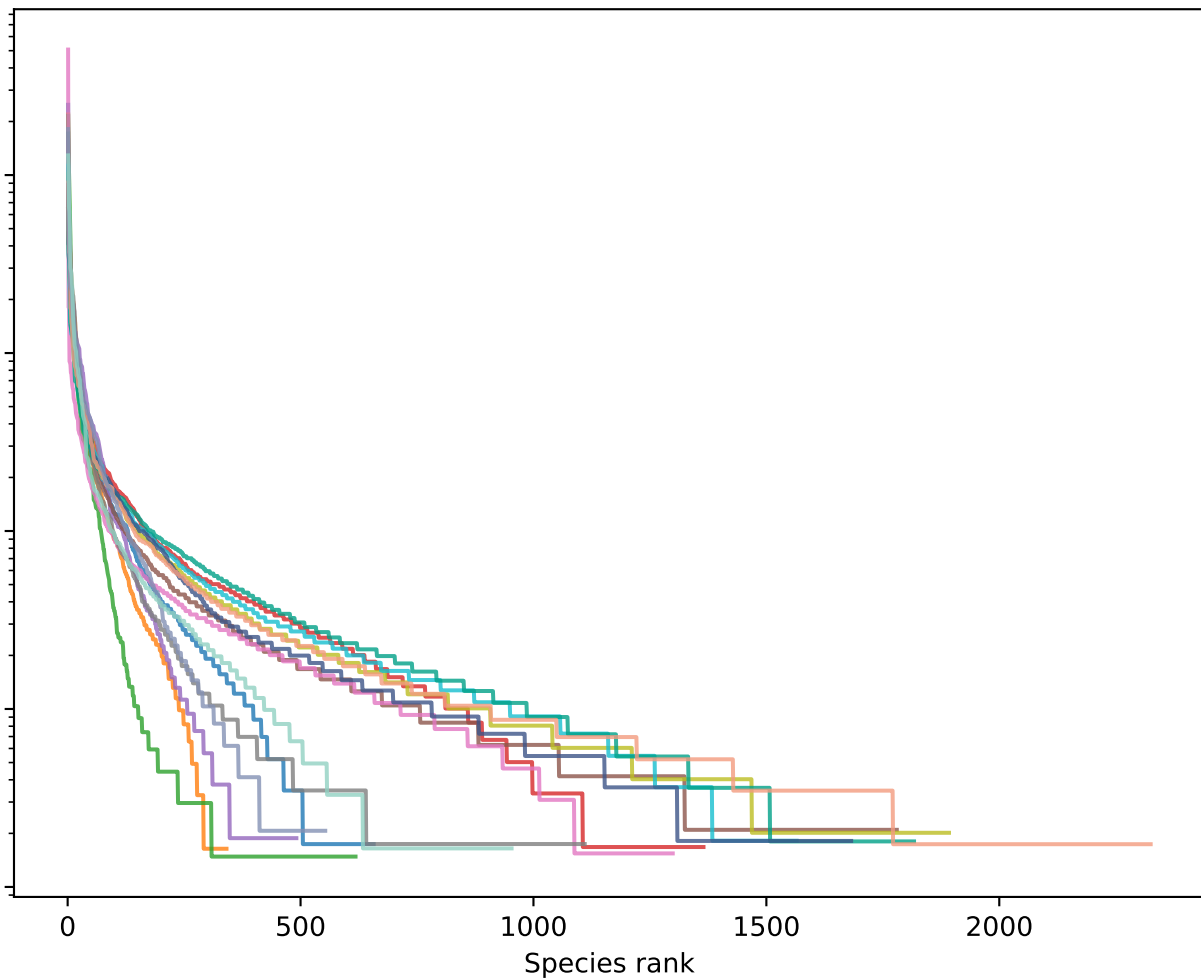

Supplement: Supplementary file 1 — customer_backup. [file MBO3-14-e70178-s001.zip › customer_backup/customer_backup/alpha_diversity/rank_abund_curve/treat/treat.rank.abund.curve.pdf]

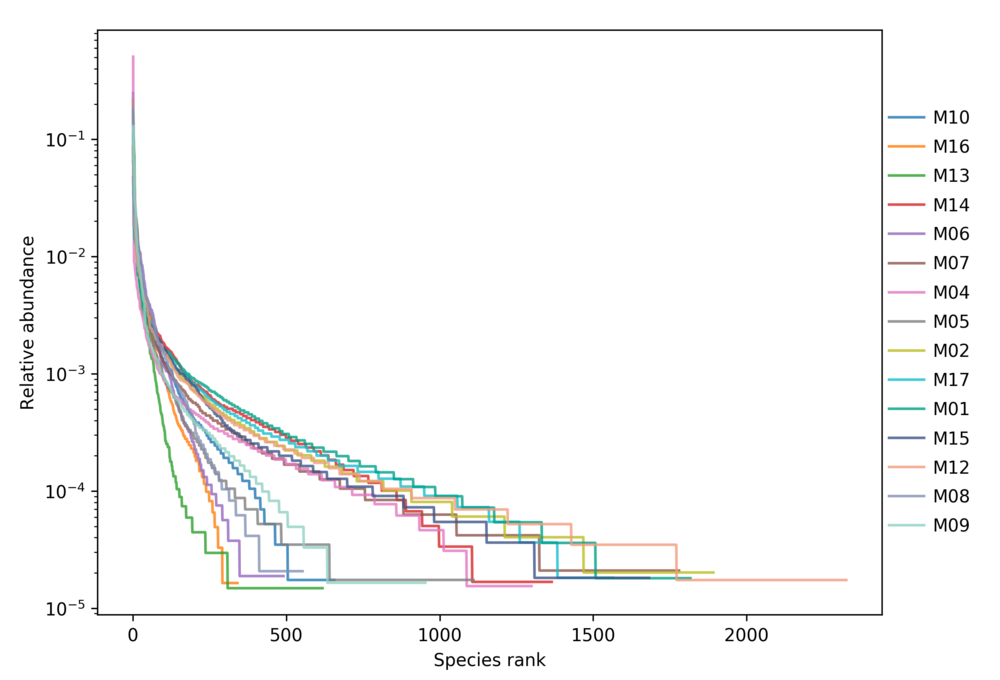

Supplement: Supplementary file 1 — customer_backup. [file MBO3-14-e70178-s001.zip › customer_backup/customer_backup/alpha_diversity/rank_abund_curve/treat/treat.rank.abund.curve_small.png]

Multi Samples Rarefaction Curves

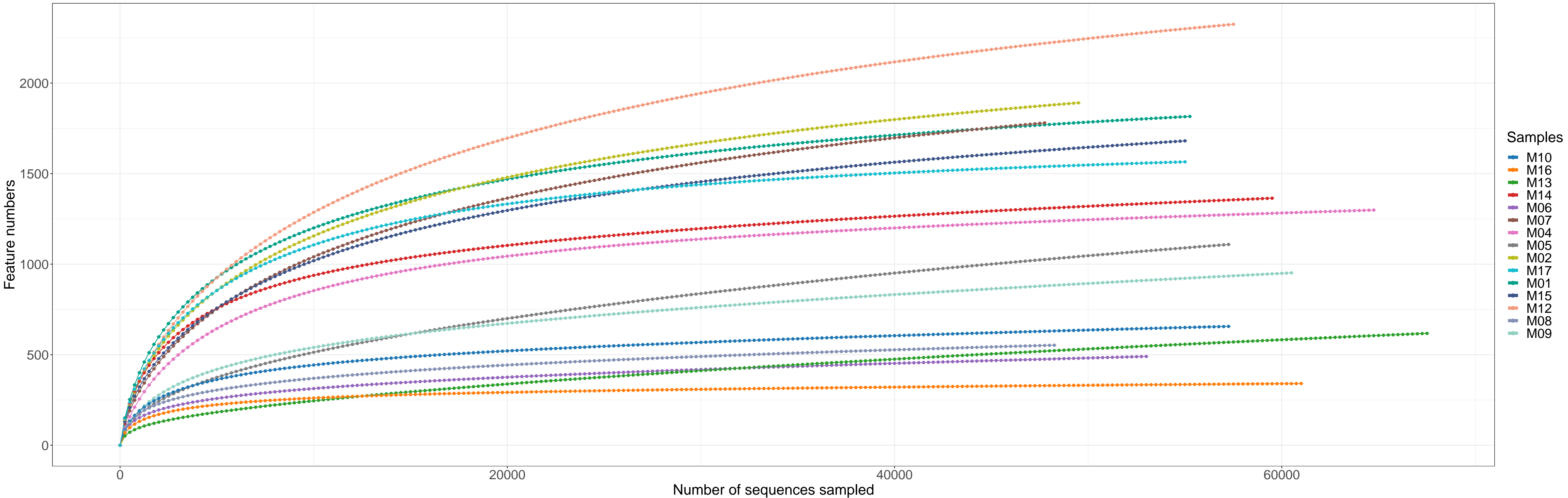

Supplement: Supplementary file 1 — customer_backup. [file MBO3-14-e70178-s001.zip › customer_backup/customer_backup/alpha_diversity/rarefaction_curve/allsample/allsample.Rarefaction.curve_group.pdf]

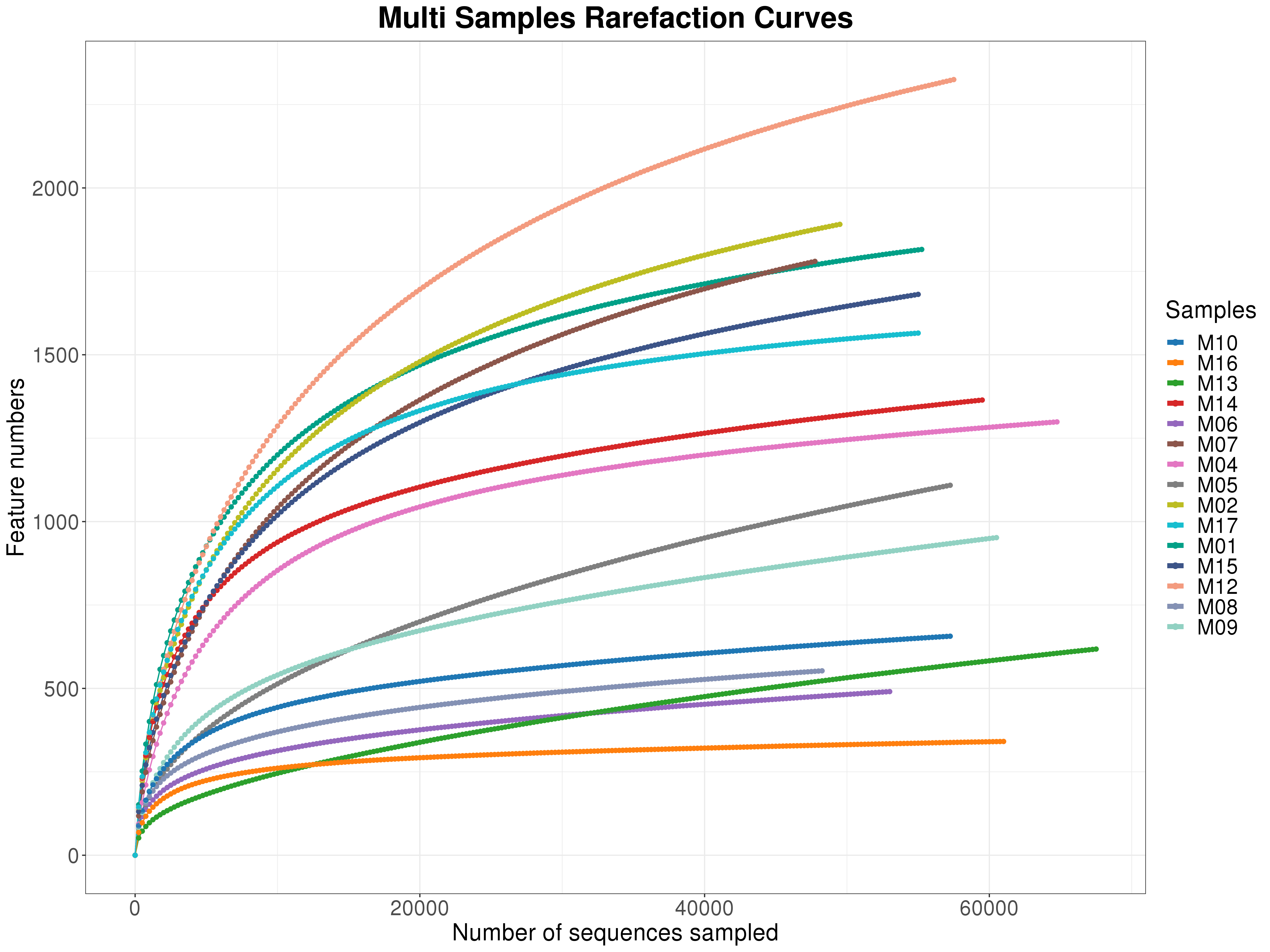

Supplement: Supplementary file 1 — customer_backup. [file MBO3-14-e70178-s001.zip › customer_backup/customer_backup/alpha_diversity/rarefaction_curve/allsample/allsample.Rarefaction.curve_group.png]

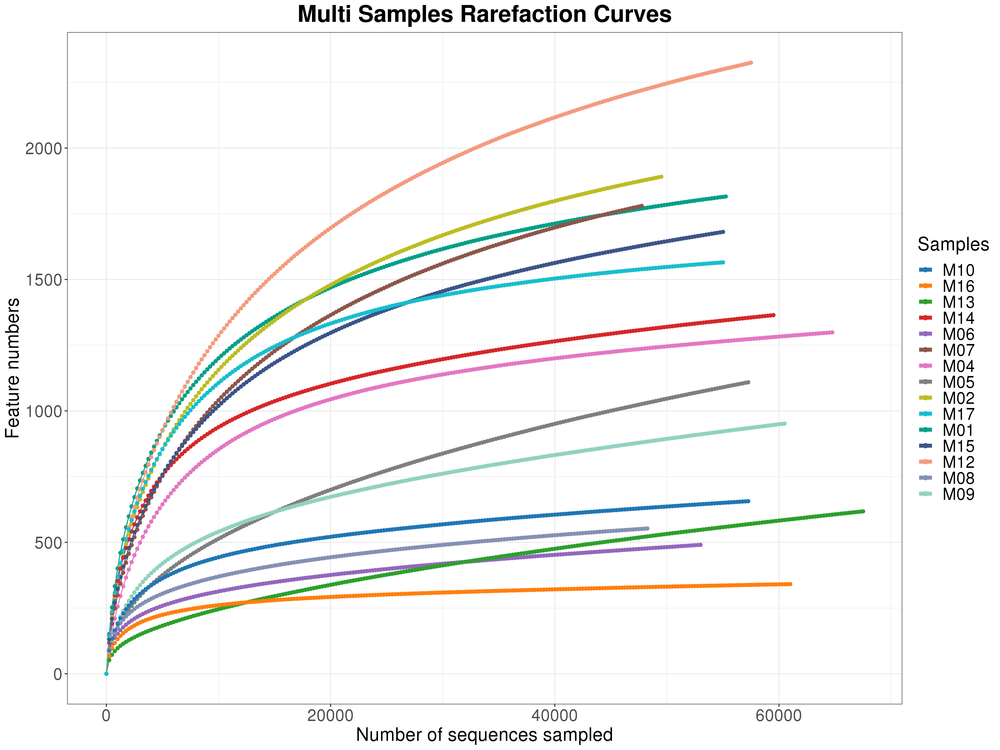

Supplement: Supplementary file 1 — customer_backup. [file MBO3-14-e70178-s001.zip › customer_backup/customer_backup/alpha_diversity/rarefaction_curve/allsample/allsample.Rarefaction.curve_group_small.png]

Multi Samples Rarefaction Curves

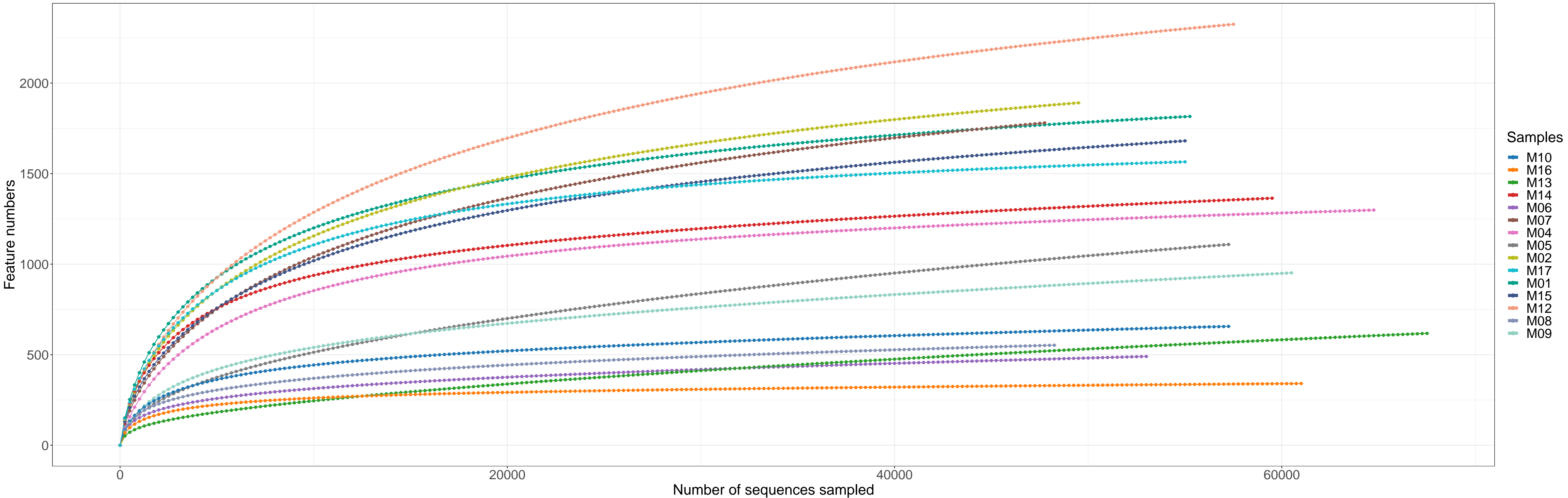

Supplement: Supplementary file 1 — customer_backup. [file MBO3-14-e70178-s001.zip › customer_backup/customer_backup/alpha_diversity/rarefaction_curve/treat/treat.Rarefaction.curve_group.pdf]

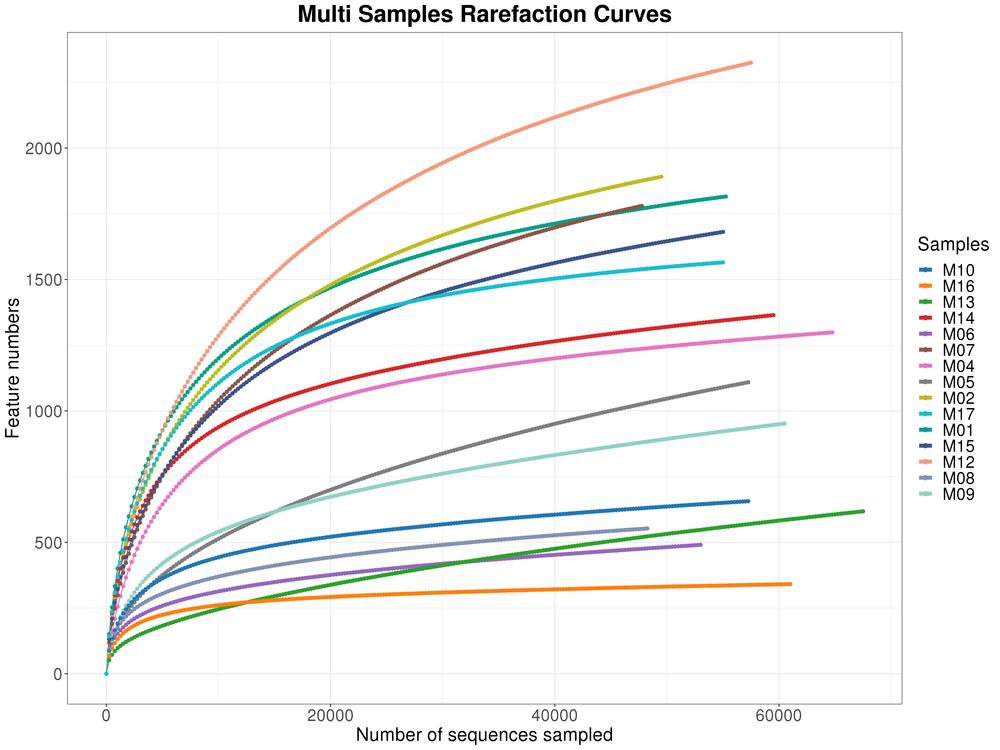

Supplement: Supplementary file 1 — customer_backup. [file MBO3-14-e70178-s001.zip › customer_backup/customer_backup/alpha_diversity/rarefaction_curve/treat/treat.Rarefaction.curve_group_small.png]

Multi Samples Shannon Curves

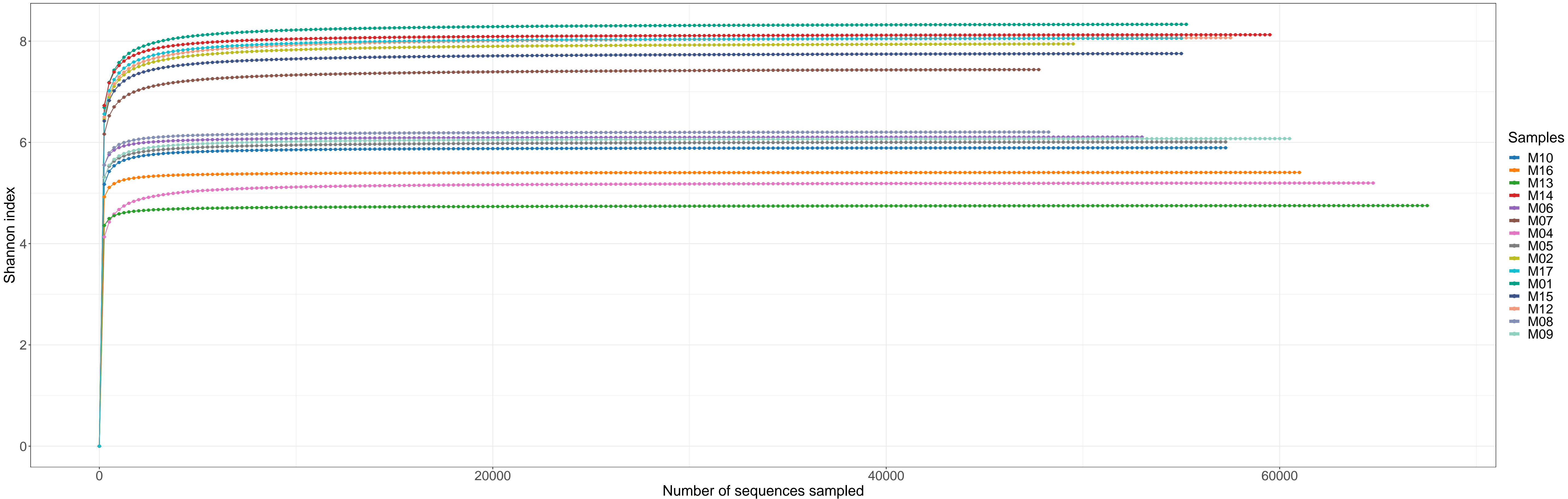

Supplement: Supplementary file 1 — customer_backup. [file MBO3-14-e70178-s001.zip › customer_backup/customer_backup/alpha_diversity/shannon_index_curve/allsample/allsample.shannon.curve_group.pdf]

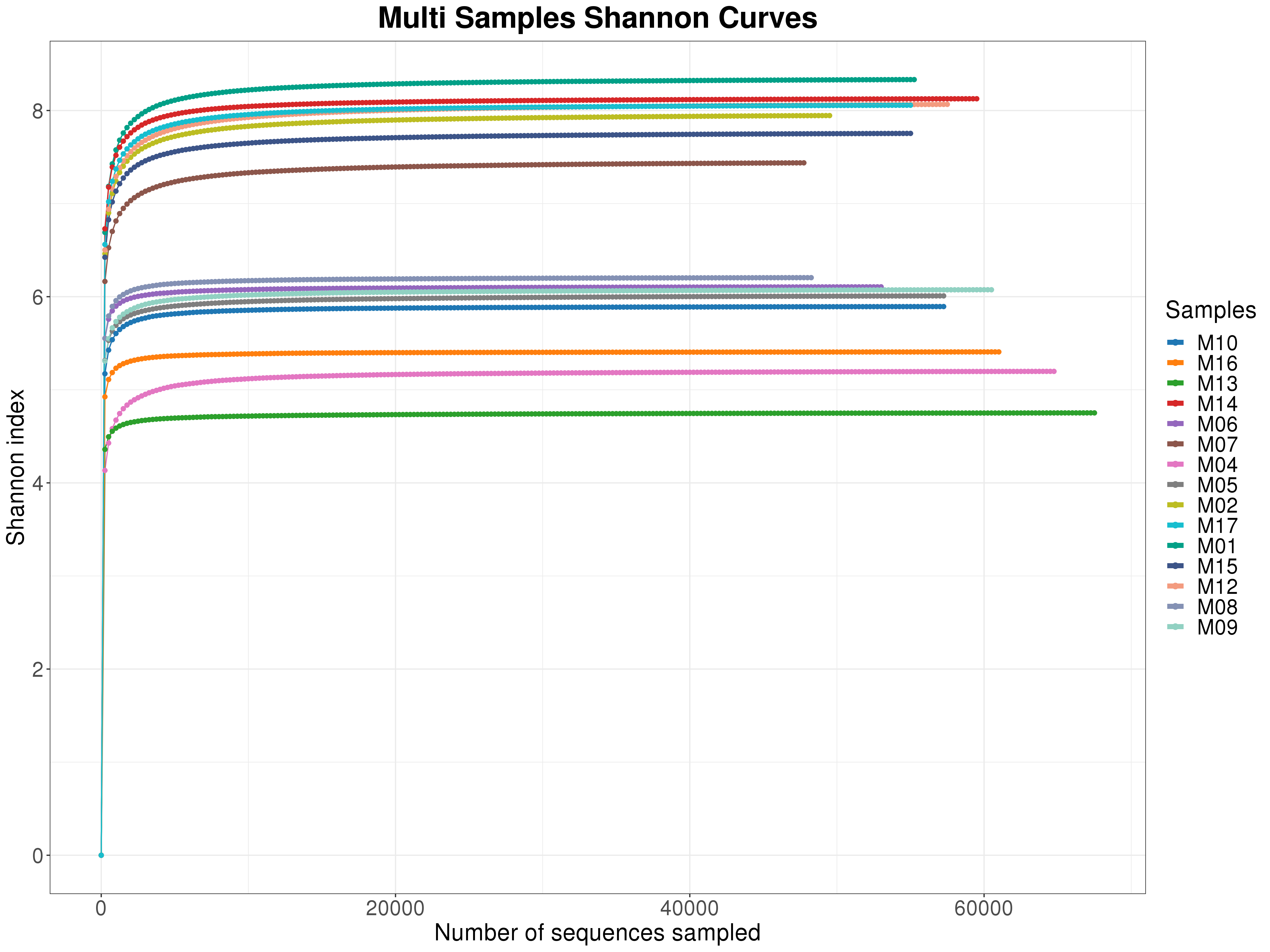

Supplement: Supplementary file 1 — customer_backup. [file MBO3-14-e70178-s001.zip › customer_backup/customer_backup/alpha_diversity/shannon_index_curve/allsample/allsample.shannon.curve_group.png]

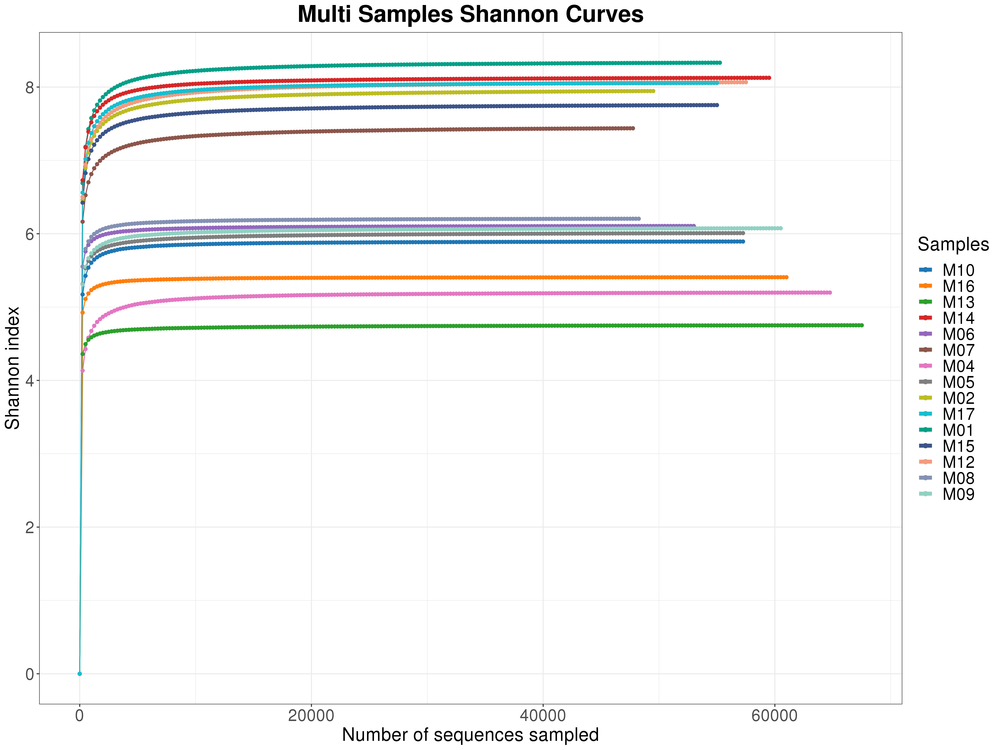

Supplement: Supplementary file 1 — customer_backup. [file MBO3-14-e70178-s001.zip › customer_backup/customer_backup/alpha_diversity/shannon_index_curve/allsample/allsample.shannon.curve_group_small.png]

Multi Samples Shannon Curves

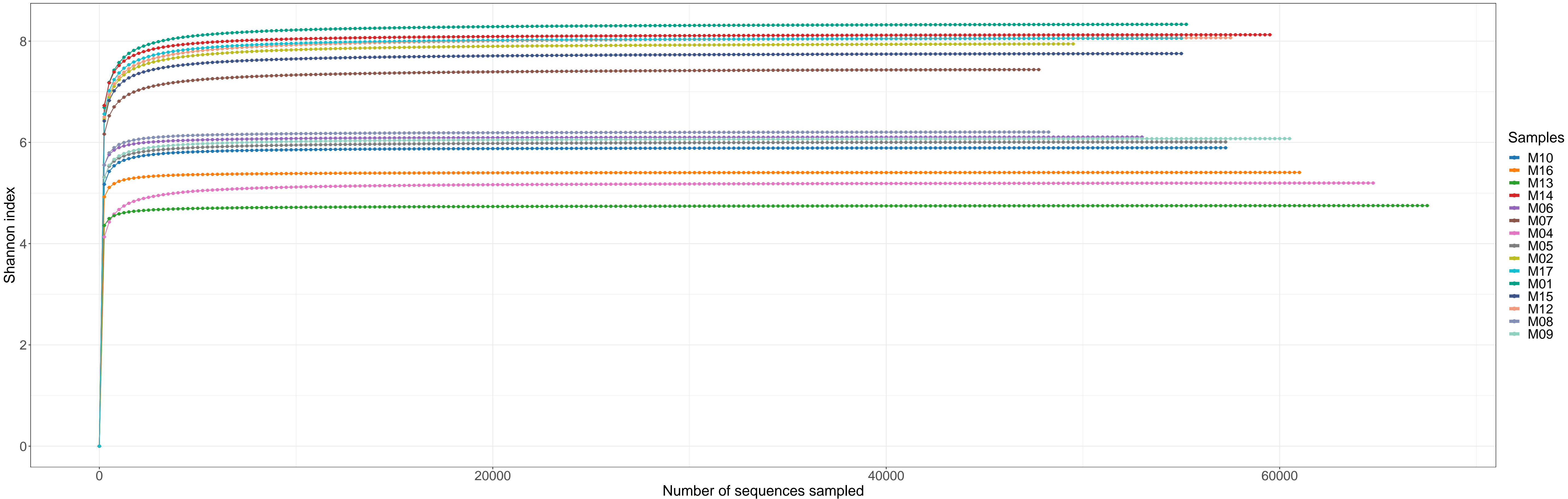

Supplement: Supplementary file 1 — customer_backup. [file MBO3-14-e70178-s001.zip › customer_backup/customer_backup/alpha_diversity/shannon_index_curve/treat/treat.shannon.curve_group.pdf]

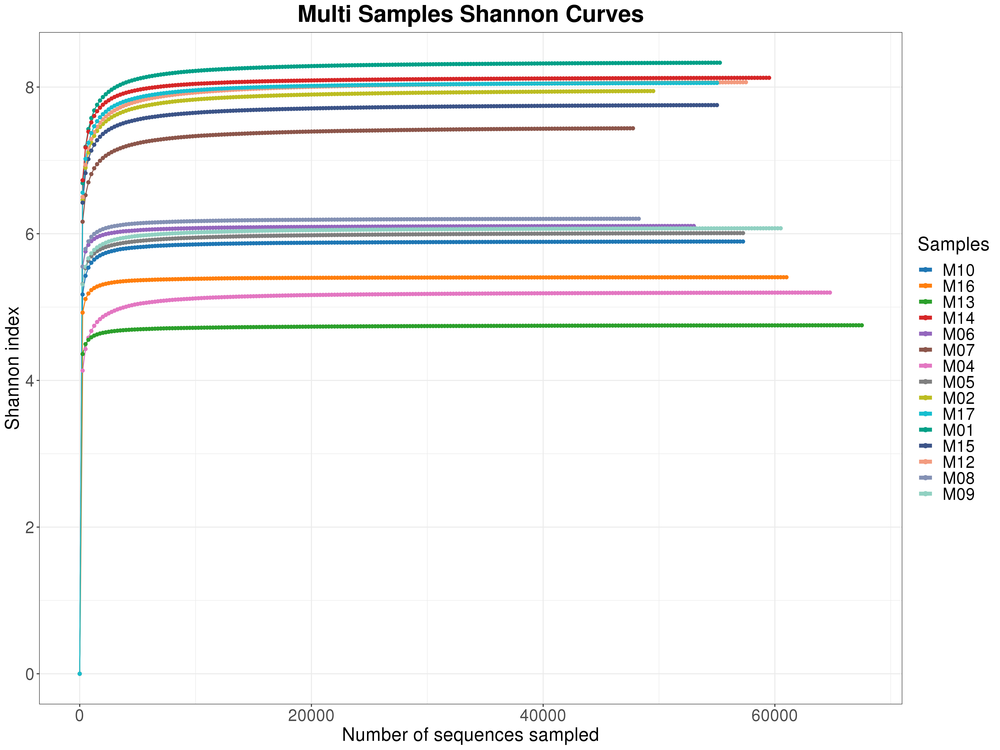

Supplement: Supplementary file 1 — customer_backup. [file MBO3-14-e70178-s001.zip › customer_backup/customer_backup/alpha_diversity/shannon_index_curve/treat/treat.shannon.curve_group_small.png]

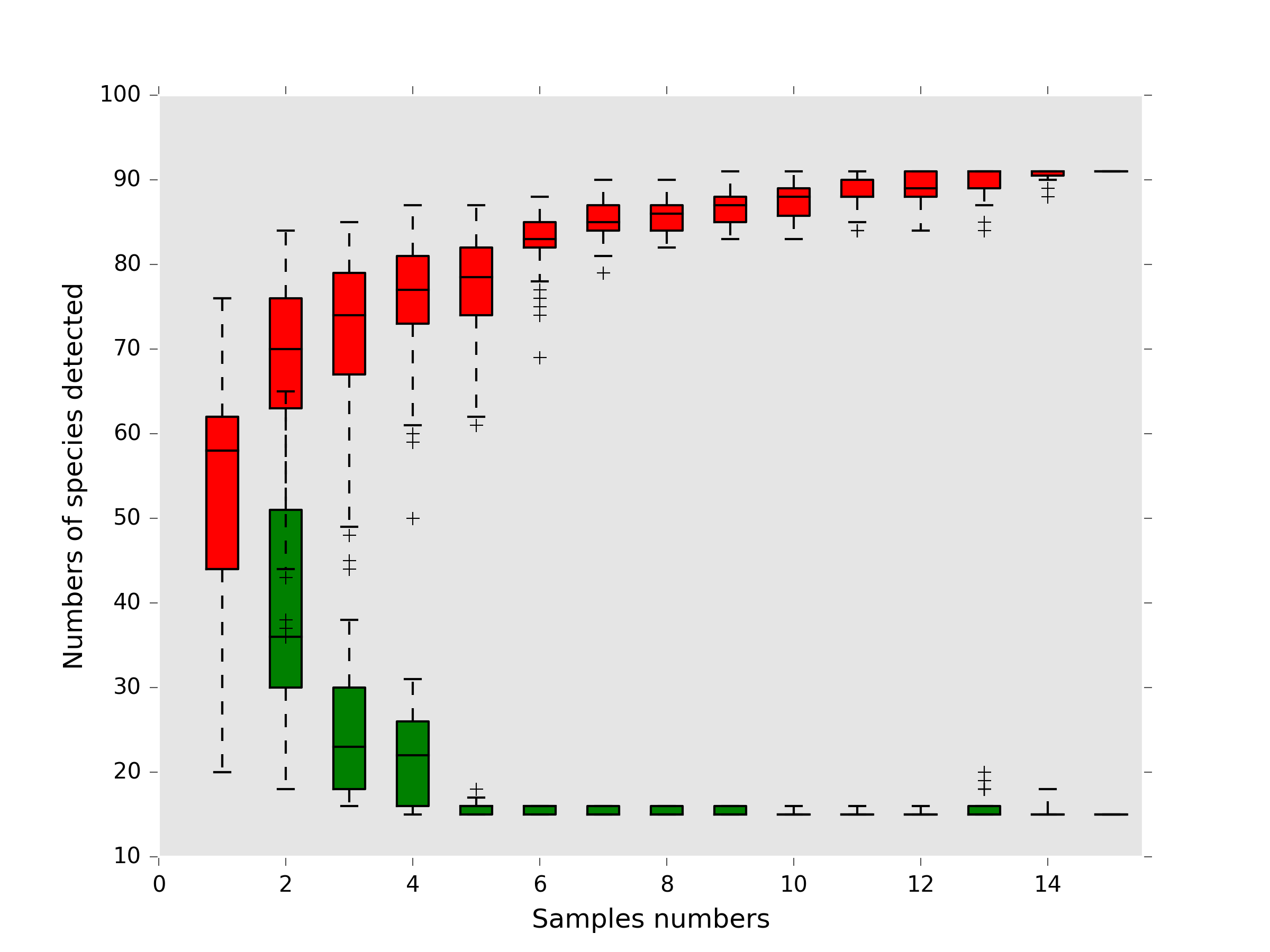

Supplement: Supplementary file 1 — customer_backup. [file MBO3-14-e70178-s001.zip › customer_backup/customer_backup/alpha_diversity/specaccum/treat/treat.class.specaccum.png]

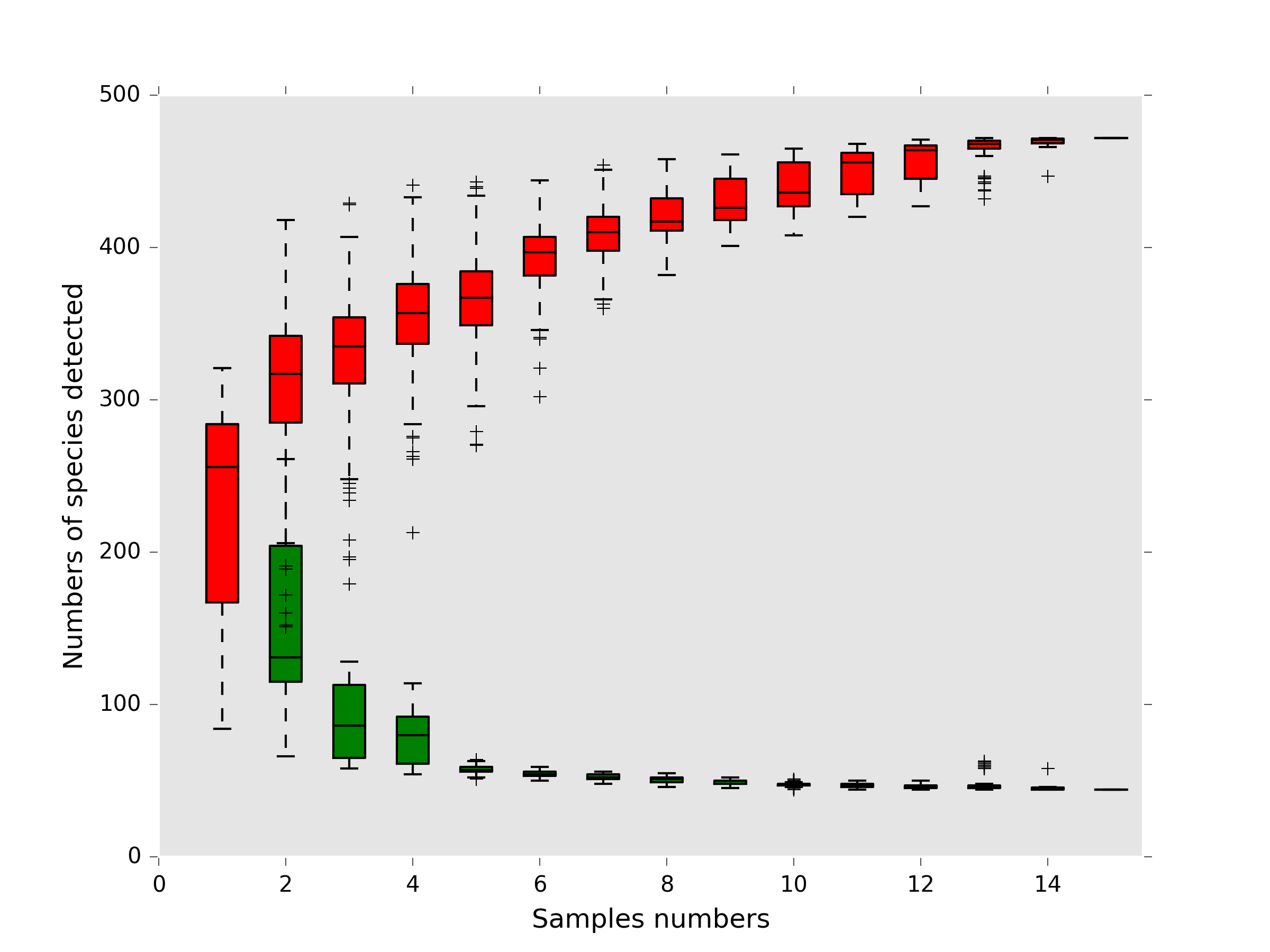

Supplement: Supplementary file 1 — customer_backup. [file MBO3-14-e70178-s001.zip › customer_backup/customer_backup/alpha_diversity/specaccum/treat/treat.family.specaccum.png]

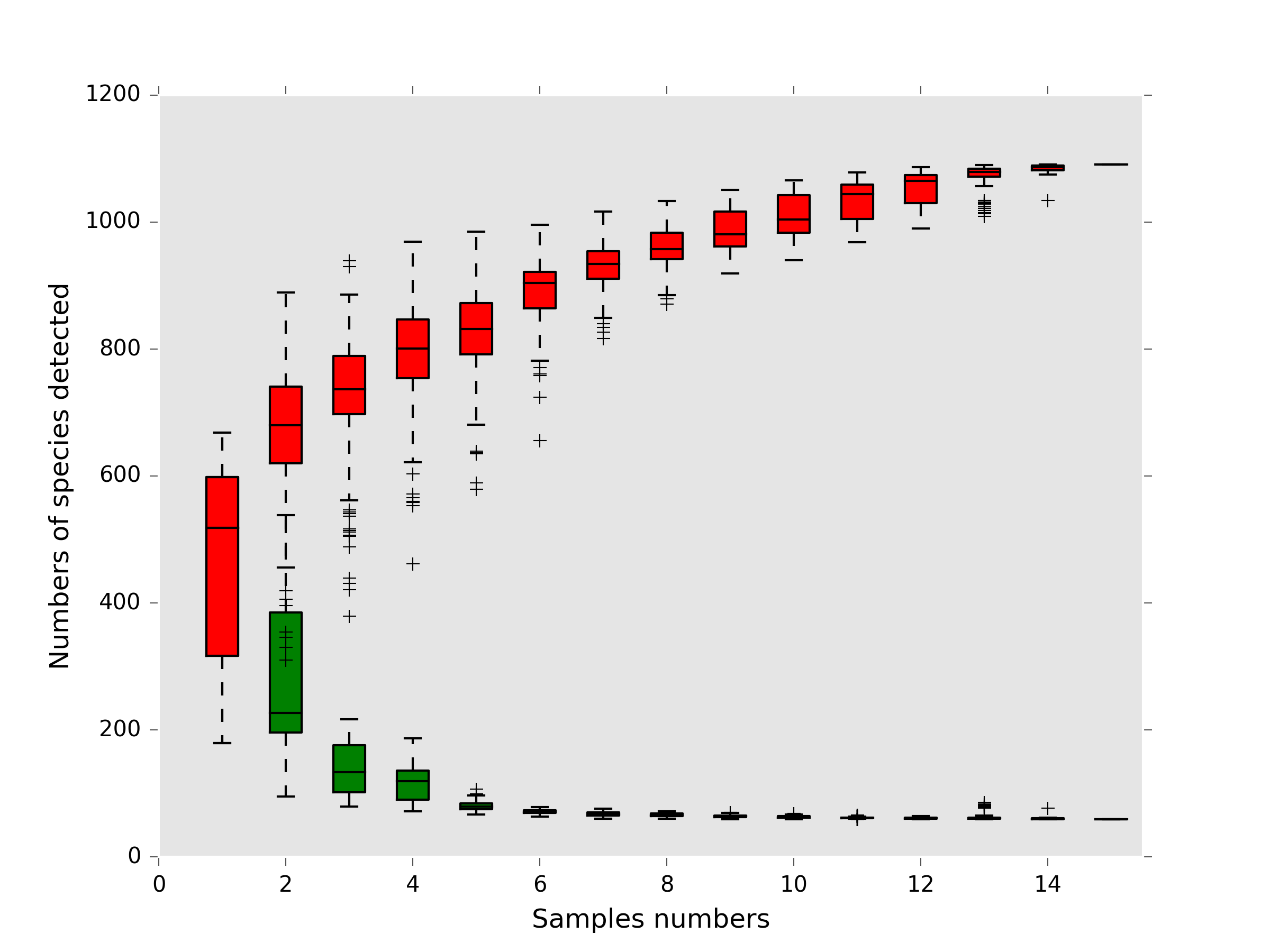

Supplement: Supplementary file 1 — customer_backup. [file MBO3-14-e70178-s001.zip › customer_backup/customer_backup/alpha_diversity/specaccum/treat/treat.genus.specaccum.png]

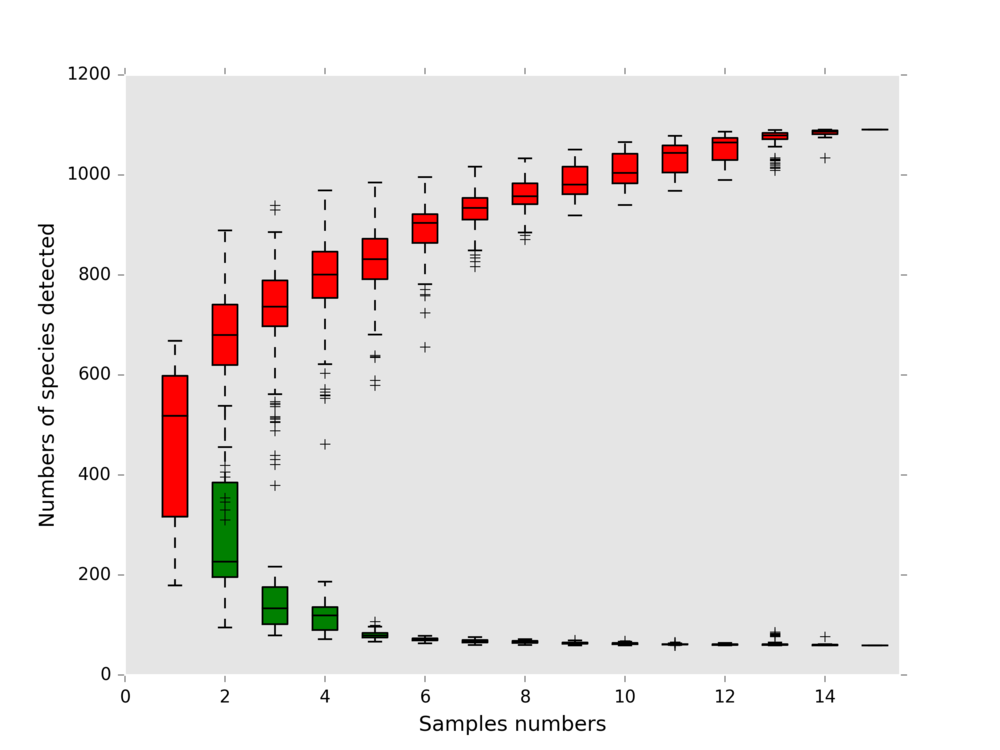

Supplement: Supplementary file 1 — customer_backup. [file MBO3-14-e70178-s001.zip › customer_backup/customer_backup/alpha_diversity/specaccum/treat/treat.genus.specaccum_small.png]

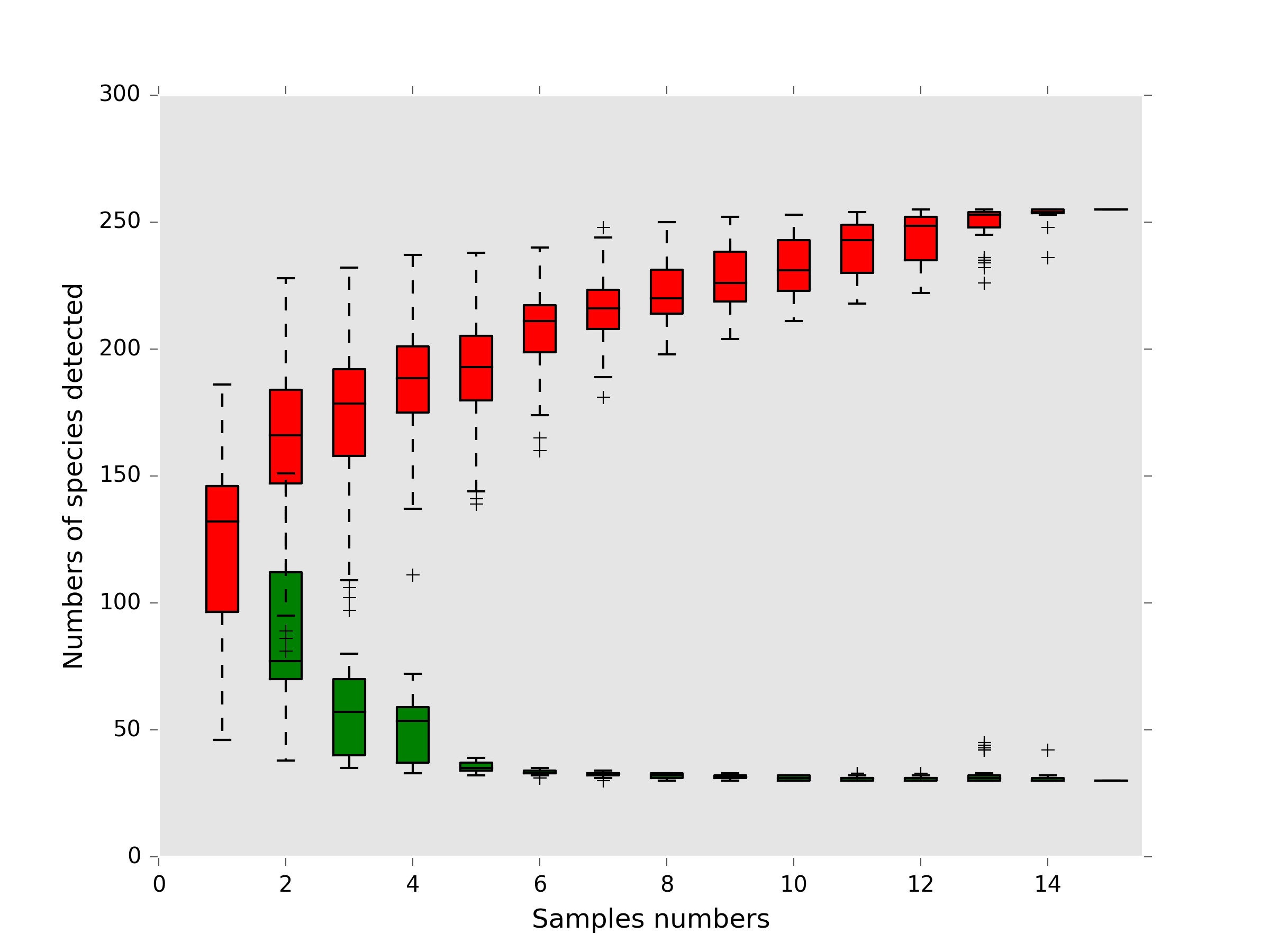

Supplement: Supplementary file 1 — customer_backup. [file MBO3-14-e70178-s001.zip › customer_backup/customer_backup/alpha_diversity/specaccum/treat/treat.order.specaccum.png]

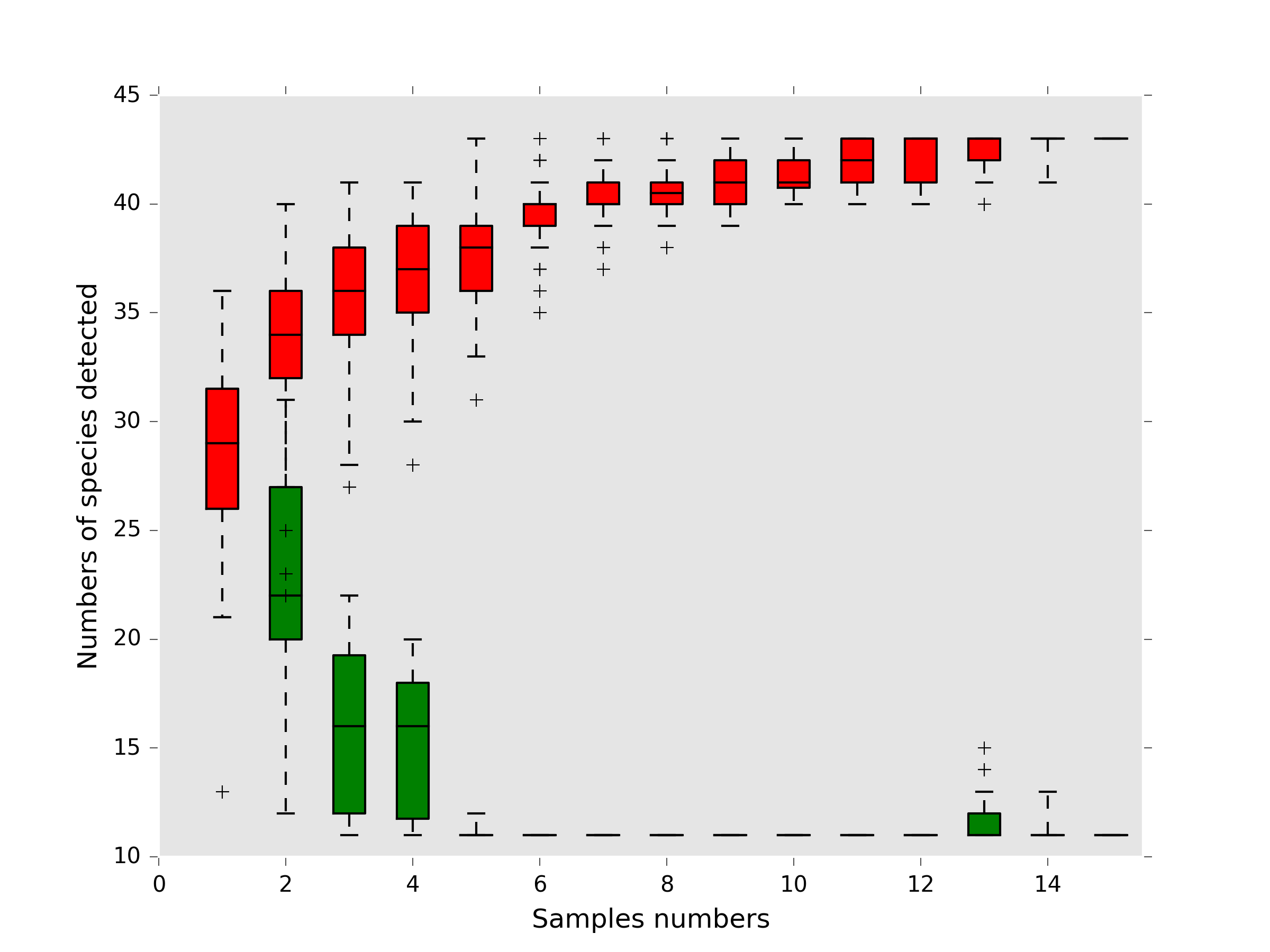

Supplement: Supplementary file 1 — customer_backup. [file MBO3-14-e70178-s001.zip › customer_backup/customer_backup/alpha_diversity/specaccum/treat/treat.phylum.specaccum.png]

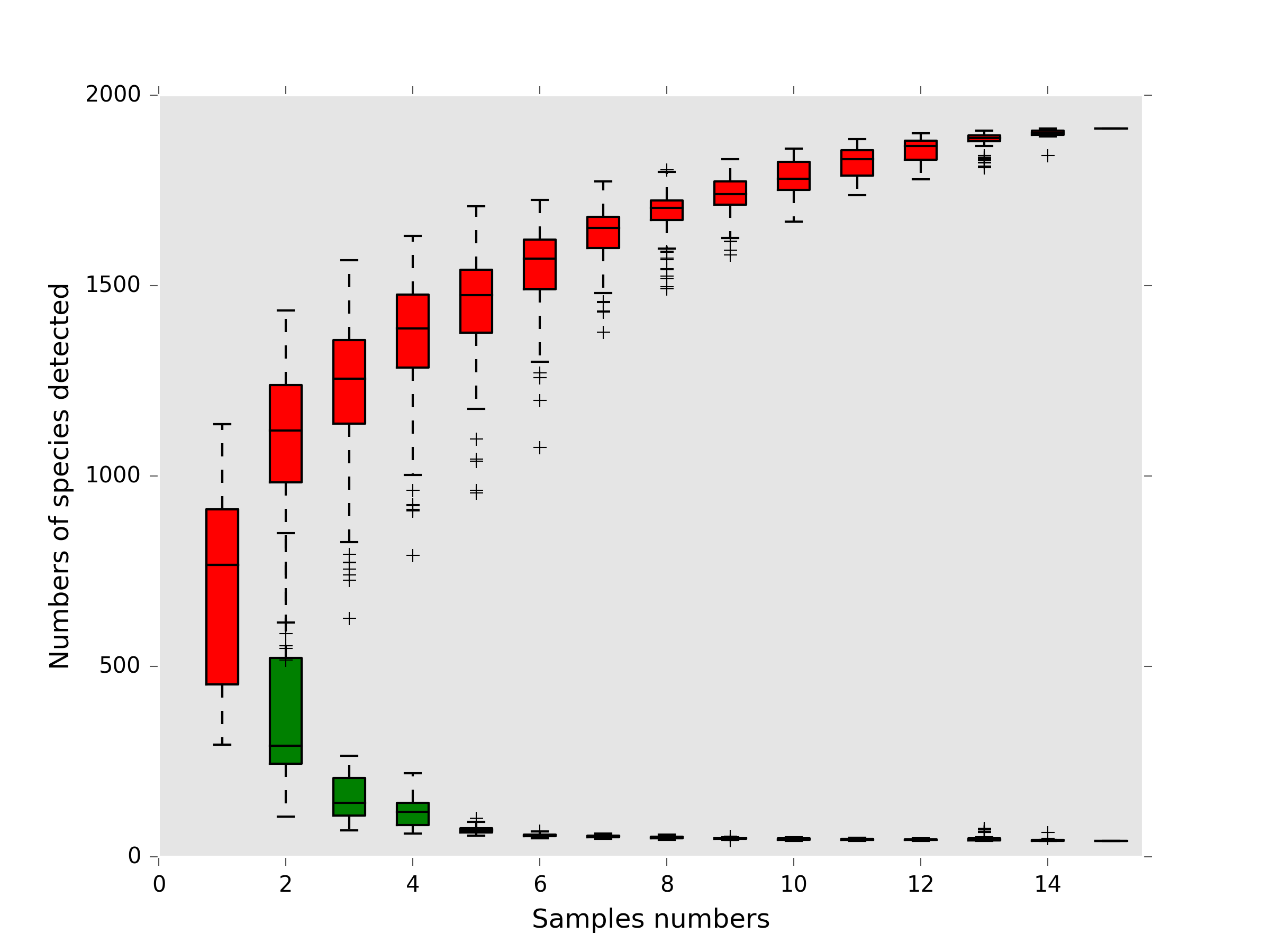

Supplement: Supplementary file 1 — customer_backup. [file MBO3-14-e70178-s001.zip › customer_backup/customer_backup/alpha_diversity/specaccum/treat/treat.species.specaccum.png]

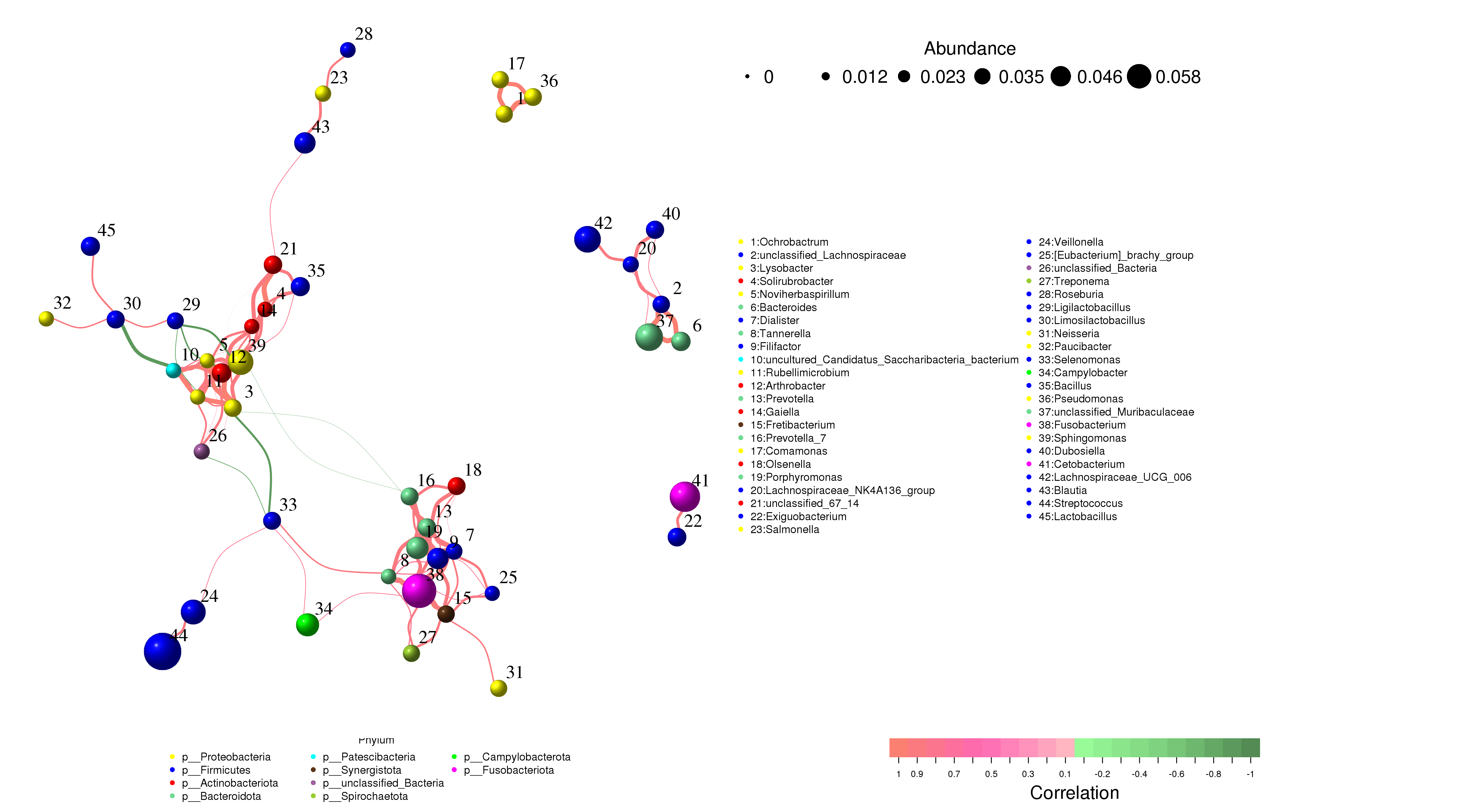

Supplement: Supplementary file 1 — customer_backup. [file MBO3-14-e70178-s001.zip › customer_backup/customer_backup/association_analysis/Network/allsample/allsample.genus.network.nicely.png]

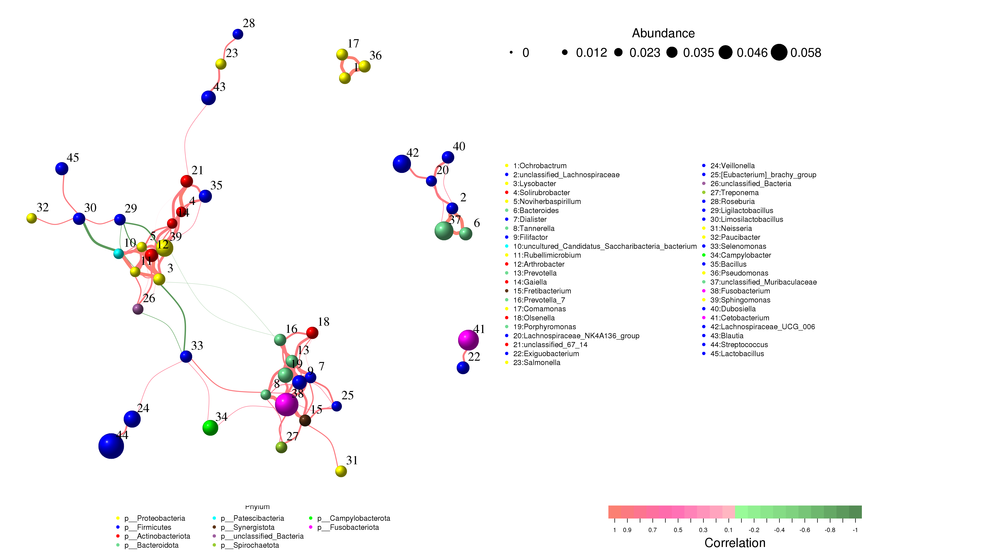

Supplement: Supplementary file 1 — customer_backup. [file MBO3-14-e70178-s001.zip › customer_backup/customer_backup/association_analysis/Network/allsample/allsample.genus.network.nicely_small.png]

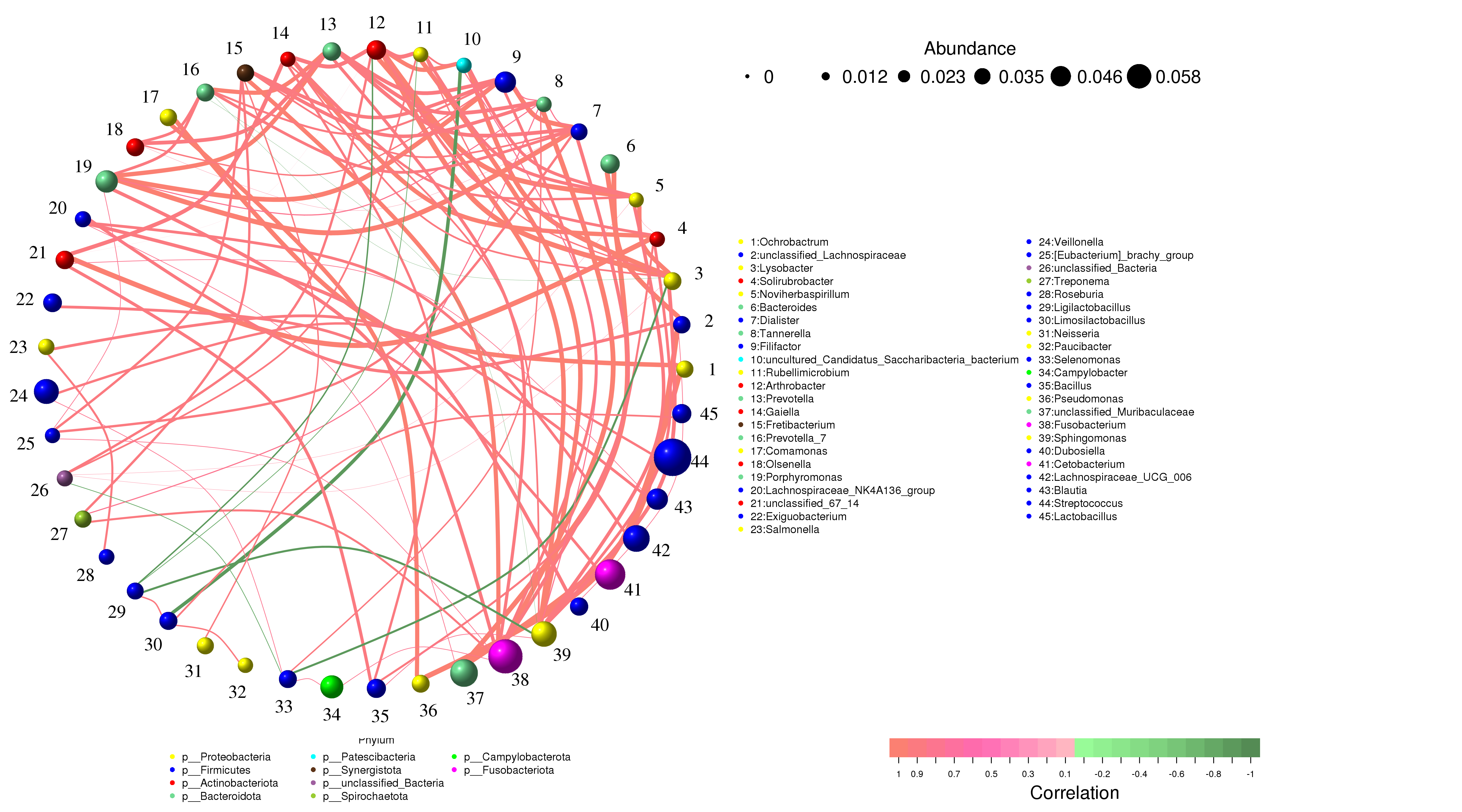

Supplement: Supplementary file 1 — customer_backup. [file MBO3-14-e70178-s001.zip › customer_backup/customer_backup/association_analysis/Network/allsample/allsample.genus.network.png]

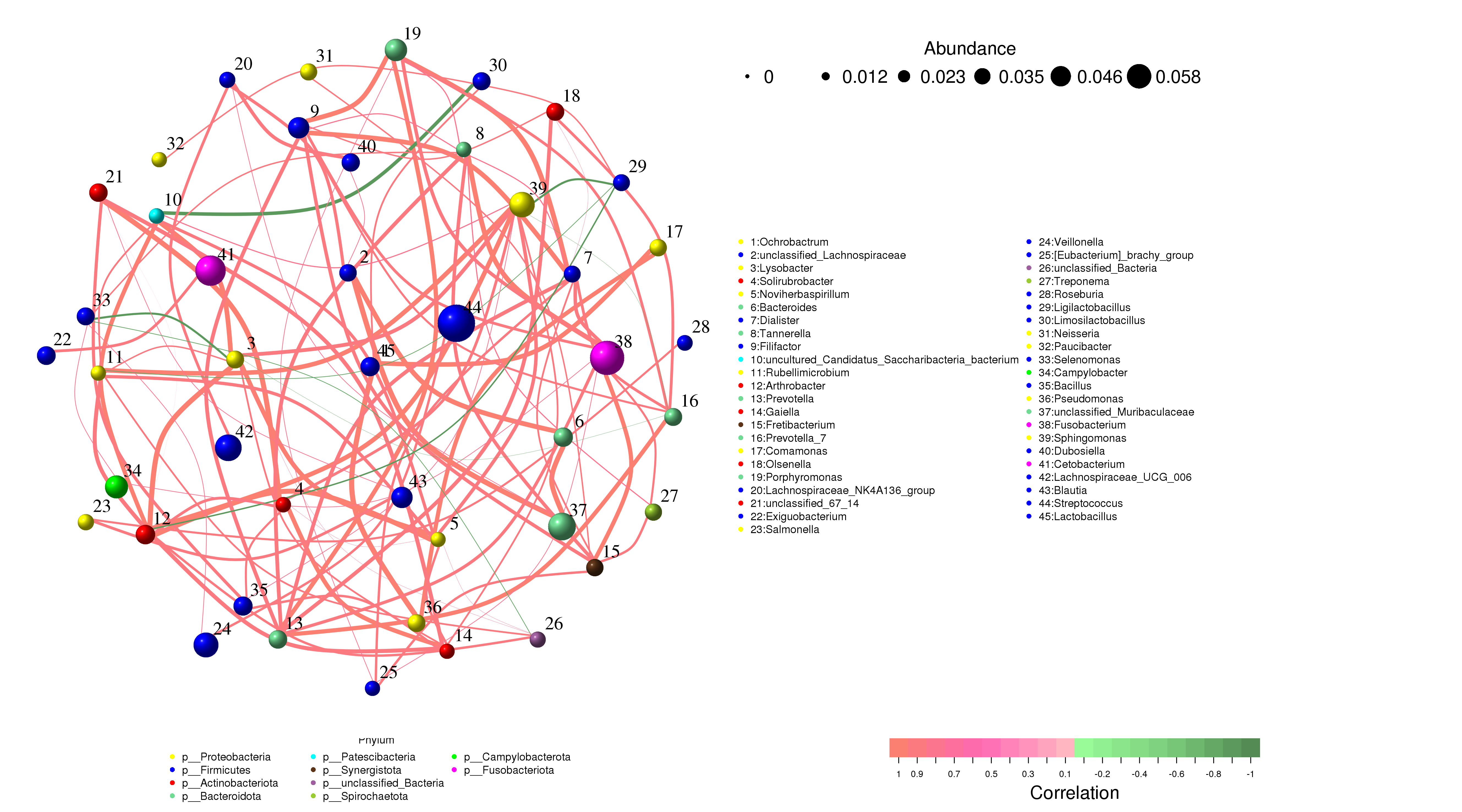

Supplement: Supplementary file 1 — customer_backup. [file MBO3-14-e70178-s001.zip › customer_backup/customer_backup/association_analysis/Network/allsample/allsample.genus.network.sphere.png]

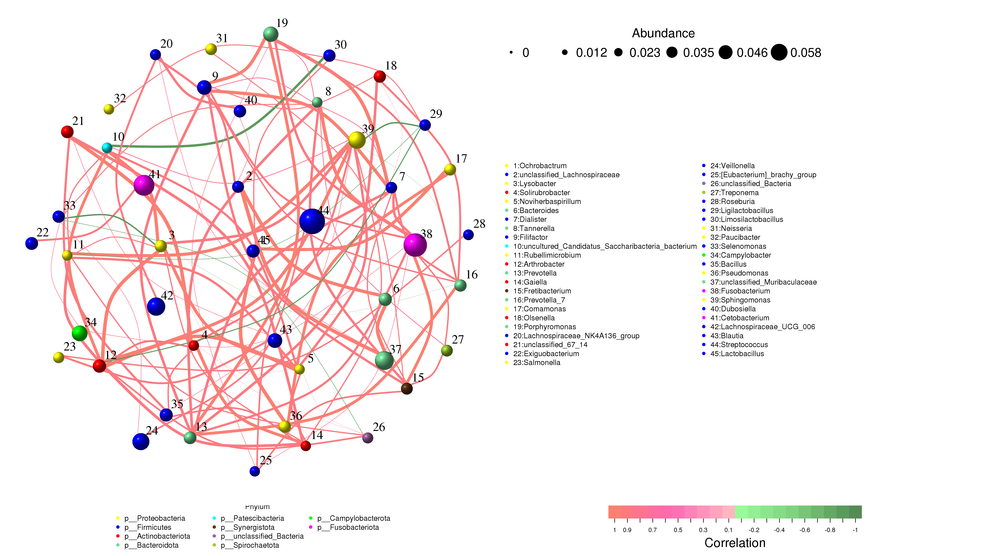

Supplement: Supplementary file 1 — customer_backup. [file MBO3-14-e70178-s001.zip › customer_backup/customer_backup/association_analysis/Network/allsample/allsample.genus.network.sphere_small.png]

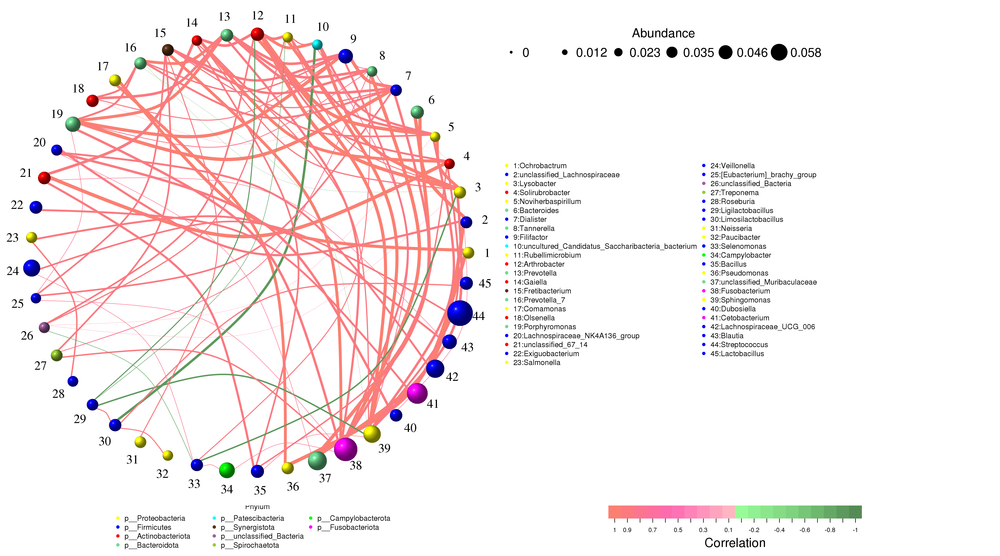

Supplement: Supplementary file 1 — customer_backup. [file MBO3-14-e70178-s001.zip › customer_backup/customer_backup/association_analysis/Network/allsample/allsample.genus.network_small.png]

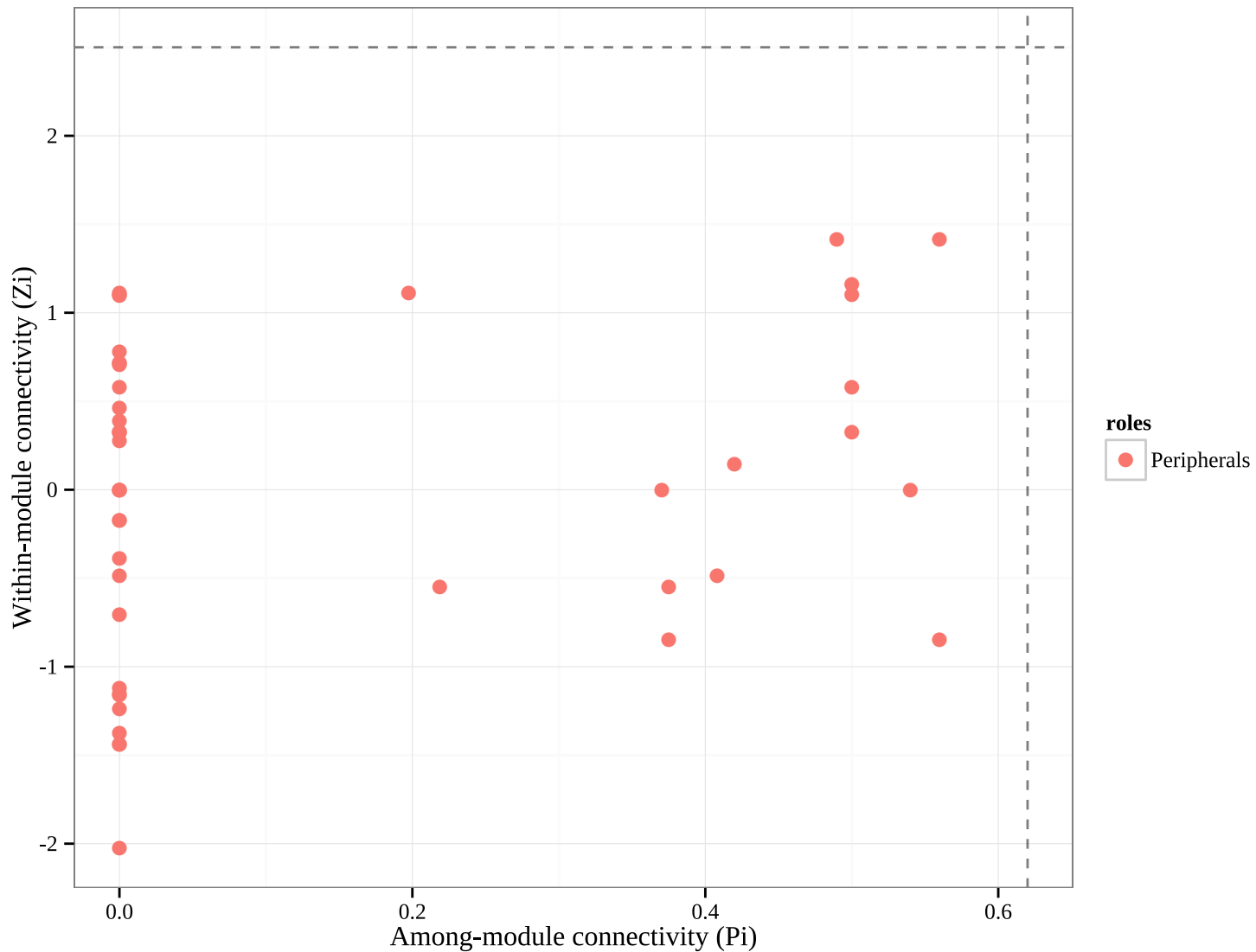

Supplement: Supplementary file 1 — customer_backup. [file MBO3-14-e70178-s001.zip › customer_backup/customer_backup/association_analysis/Network/allsample/allsample.ZiPi.pdf]

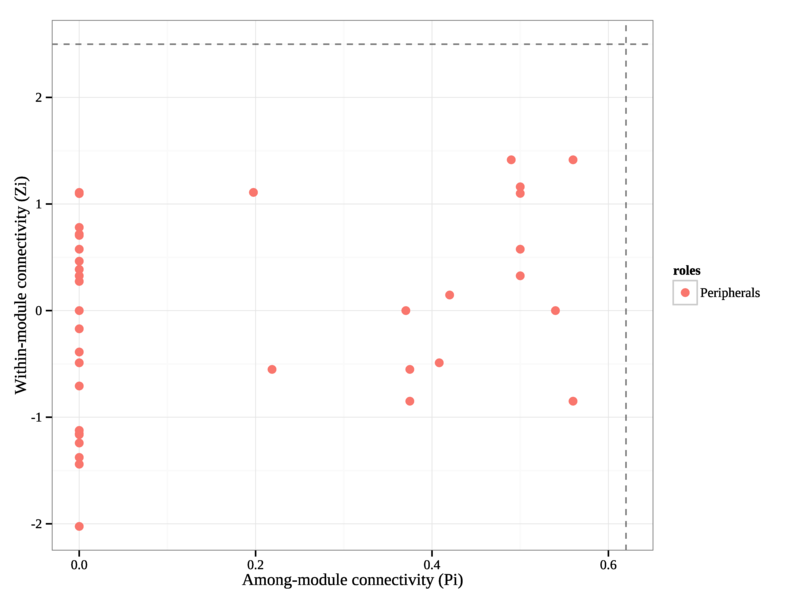

Supplement: Supplementary file 1 — customer_backup. [file MBO3-14-e70178-s001.zip › customer_backup/customer_backup/association_analysis/Network/allsample/allsample.ZiPi.png]

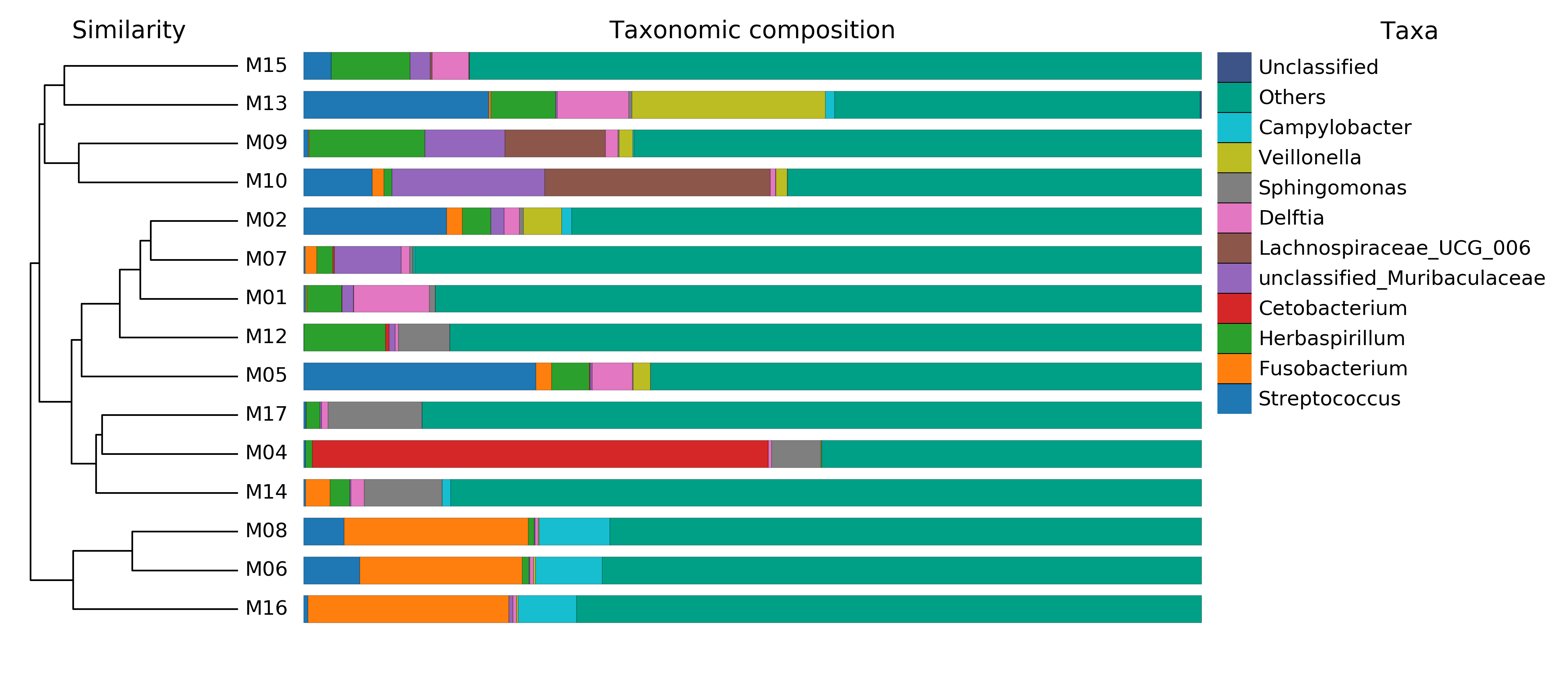

Supplement: Supplementary file 1 — customer_backup. [file MBO3-14-e70178-s001.zip › customer_backup/customer_backup/beta_diversity/ClusterTree_bar/treat/treat.binary_jaccard.png]

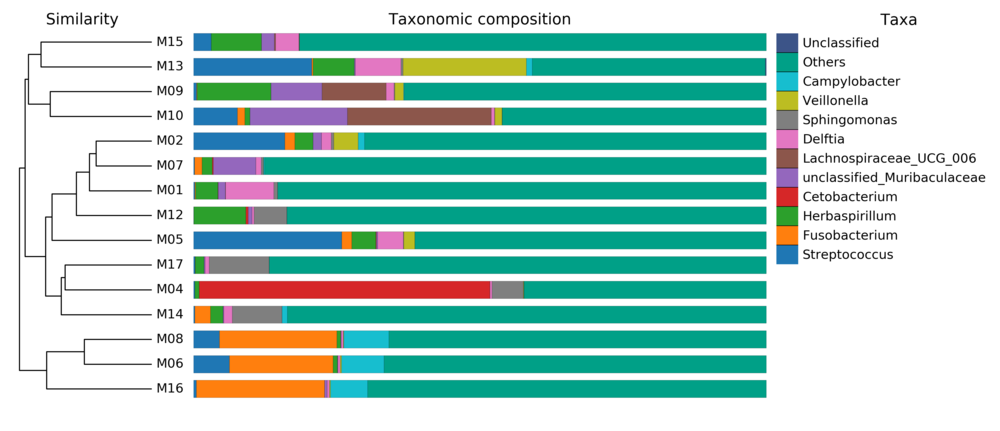

Supplement: Supplementary file 1 — customer_backup. [file MBO3-14-e70178-s001.zip › customer_backup/customer_backup/beta_diversity/ClusterTree_bar/treat/treat.binary_jaccard_small.png]

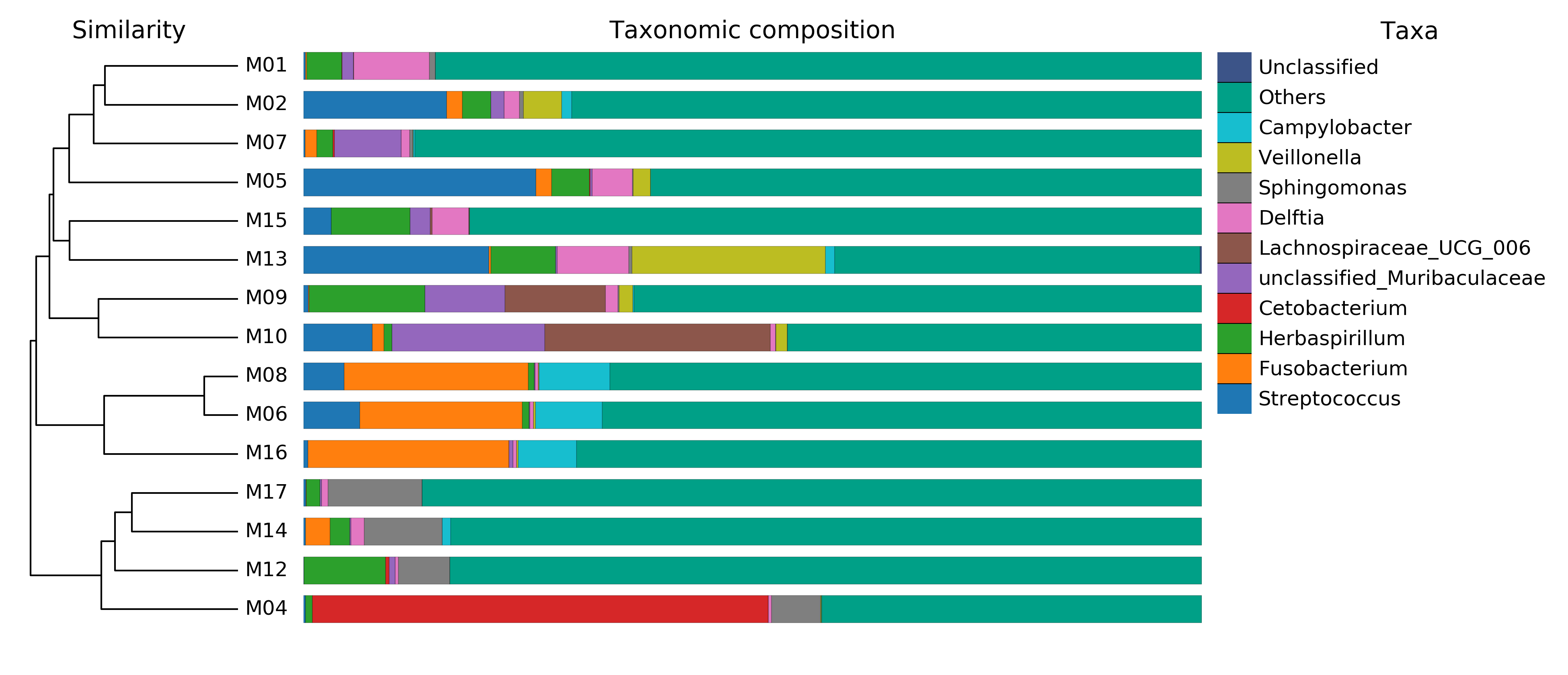

Supplement: Supplementary file 1 — customer_backup. [file MBO3-14-e70178-s001.zip › customer_backup/customer_backup/beta_diversity/ClusterTree_bar/treat/treat.bray_curtis.png]

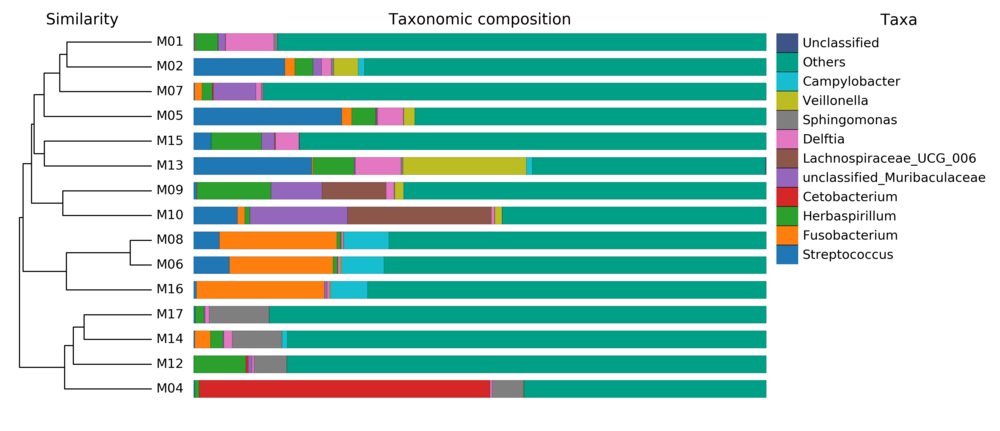

Supplement: Supplementary file 1 — customer_backup. [file MBO3-14-e70178-s001.zip › customer_backup/customer_backup/beta_diversity/ClusterTree_bar/treat/treat.bray_curtis_small.png]

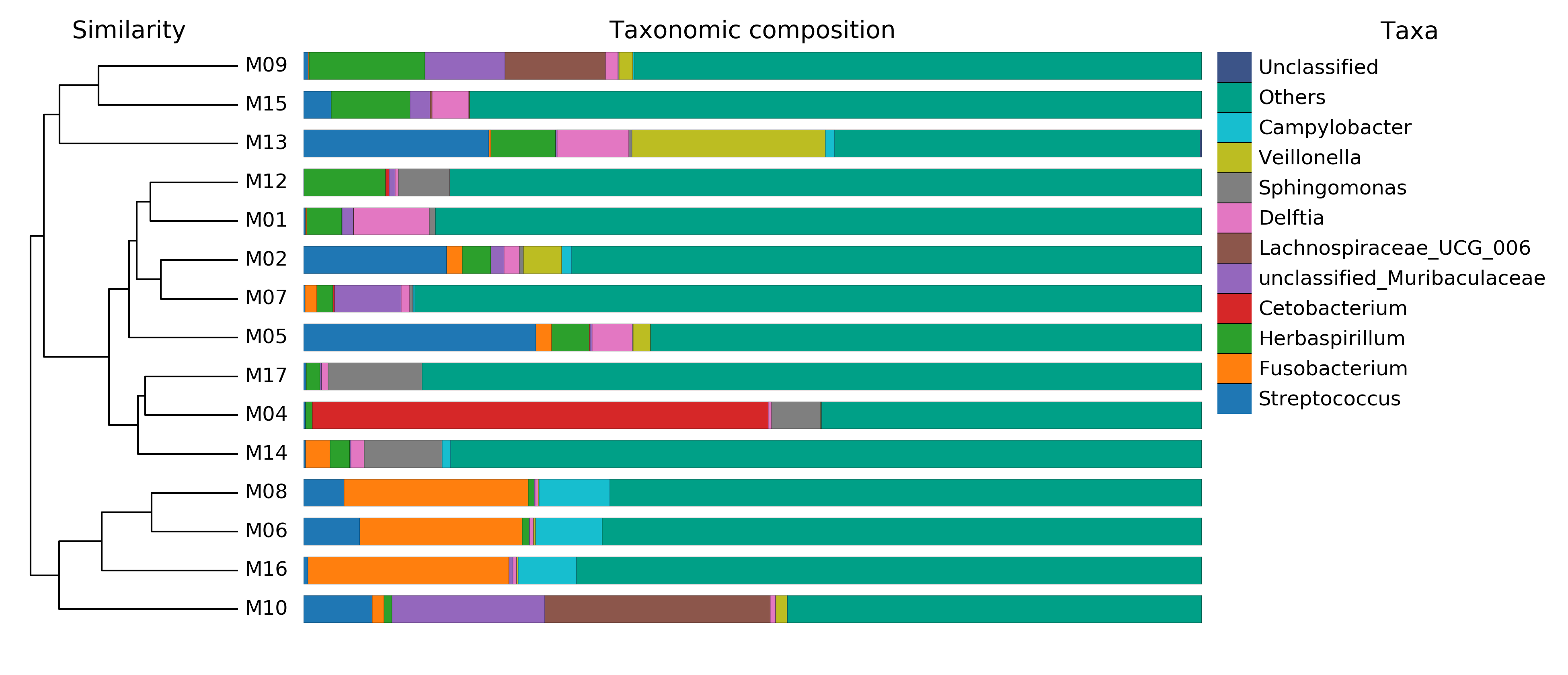

Supplement: Supplementary file 1 — customer_backup. [file MBO3-14-e70178-s001.zip › customer_backup/customer_backup/beta_diversity/ClusterTree_bar/treat/treat.unweighted_unifrac.png]

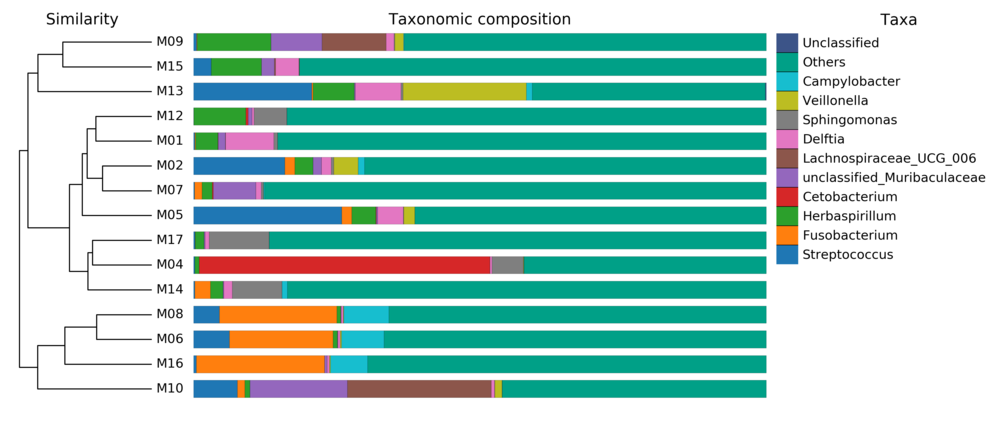

Supplement: Supplementary file 1 — customer_backup. [file MBO3-14-e70178-s001.zip › customer_backup/customer_backup/beta_diversity/ClusterTree_bar/treat/treat.unweighted_unifrac_small.png]

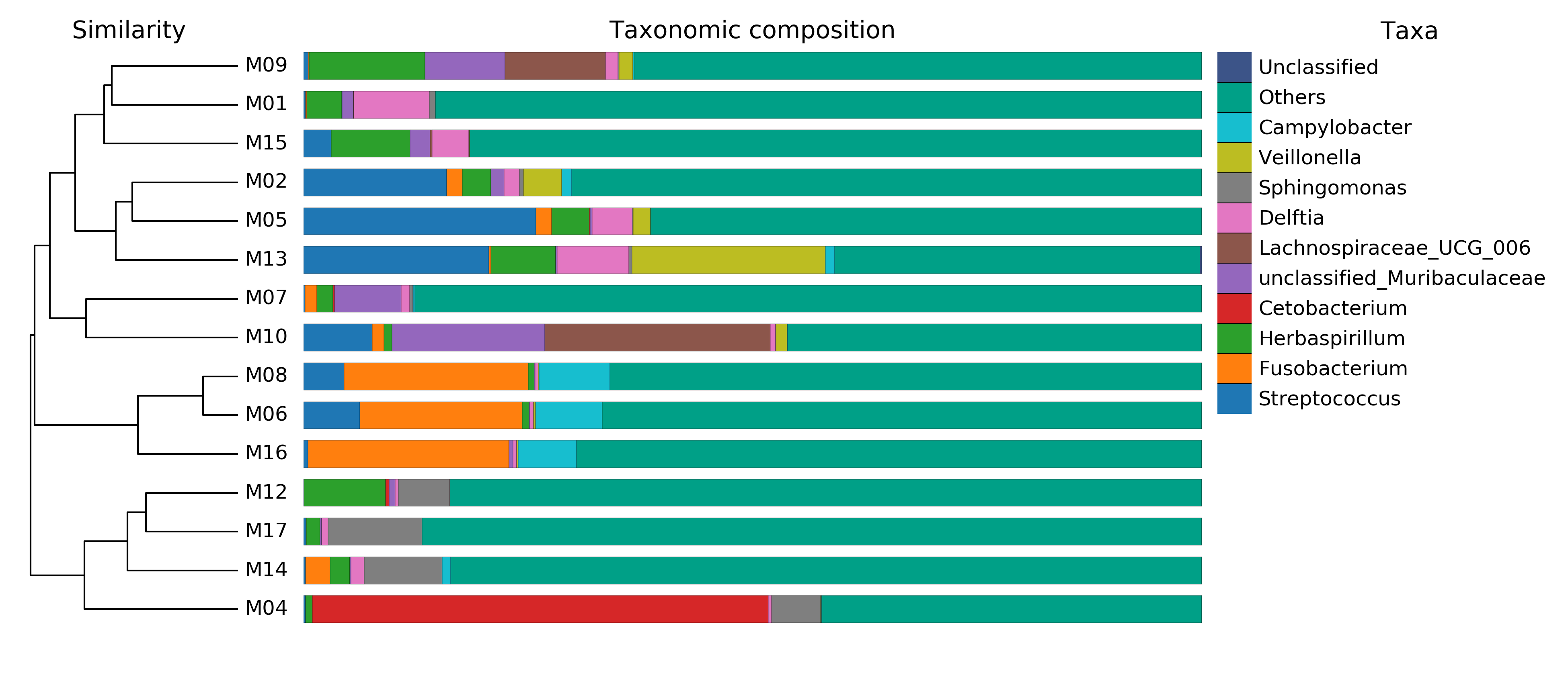

Supplement: Supplementary file 1 — customer_backup. [file MBO3-14-e70178-s001.zip › customer_backup/customer_backup/beta_diversity/ClusterTree_bar/treat/treat.weighted_unifrac.png]

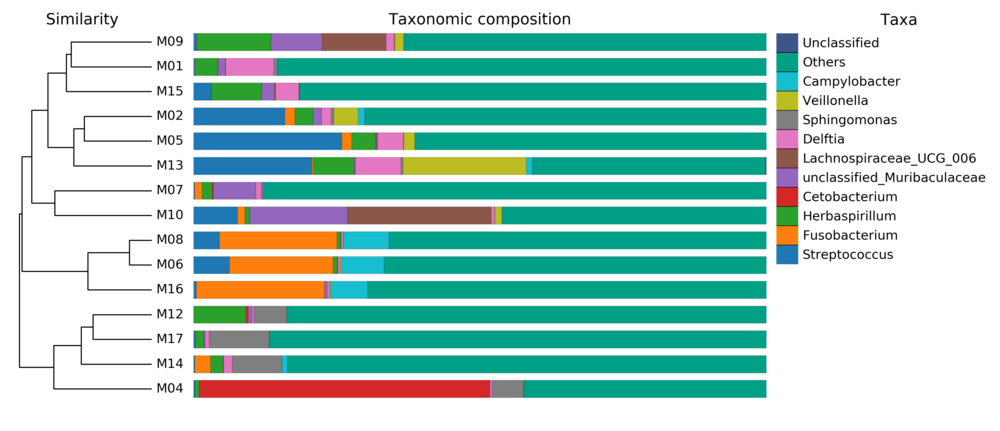

Supplement: Supplementary file 1 — customer_backup. [file MBO3-14-e70178-s001.zip › customer_backup/customer_backup/beta_diversity/ClusterTree_bar/treat/treat.weighted_unifrac_small.png]

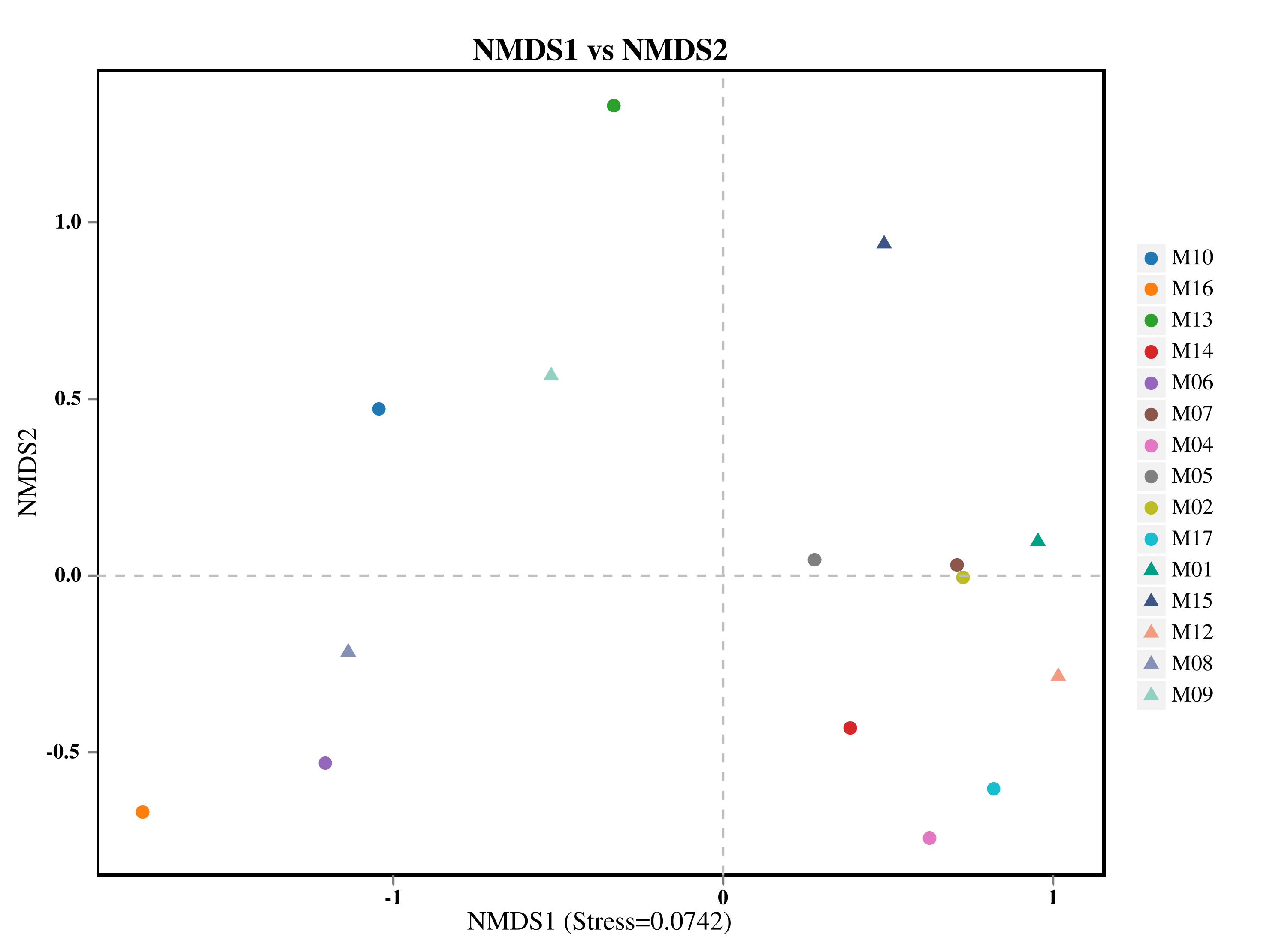

Supplement: Supplementary file 1 — customer_backup. [file MBO3-14-e70178-s001.zip › customer_backup/customer_backup/beta_diversity/nmds/treat/treat.binary_jaccard.NMDS1_NMDS2.png]

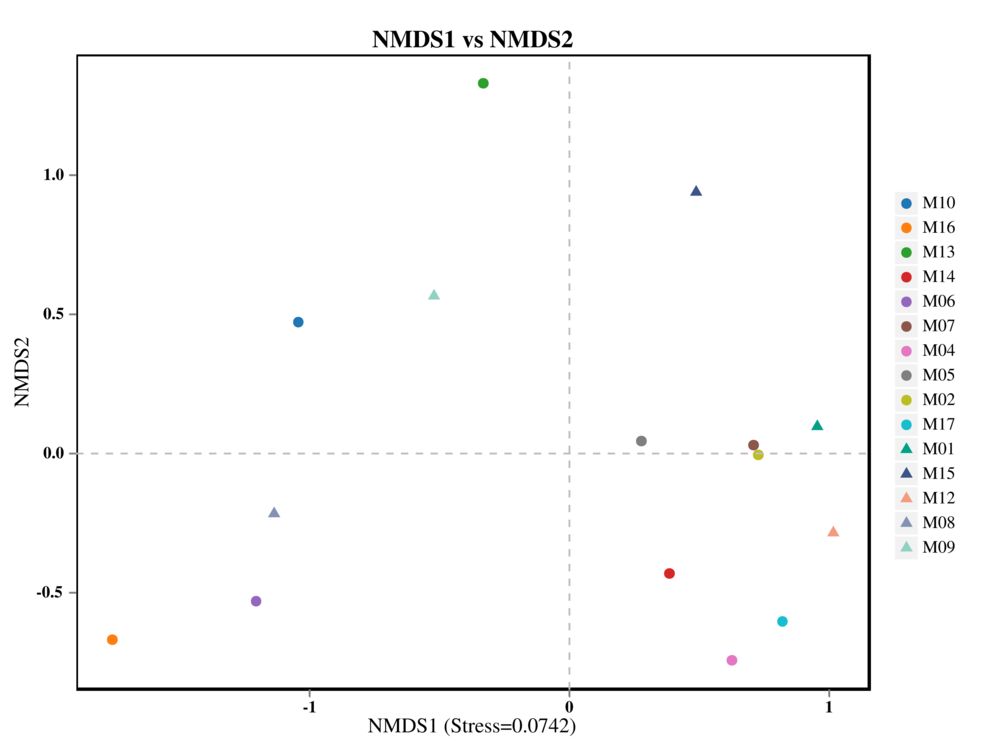

Supplement: Supplementary file 1 — customer_backup. [file MBO3-14-e70178-s001.zip › customer_backup/customer_backup/beta_diversity/nmds/treat/treat.binary_jaccard.NMDS1_NMDS2_small.png]

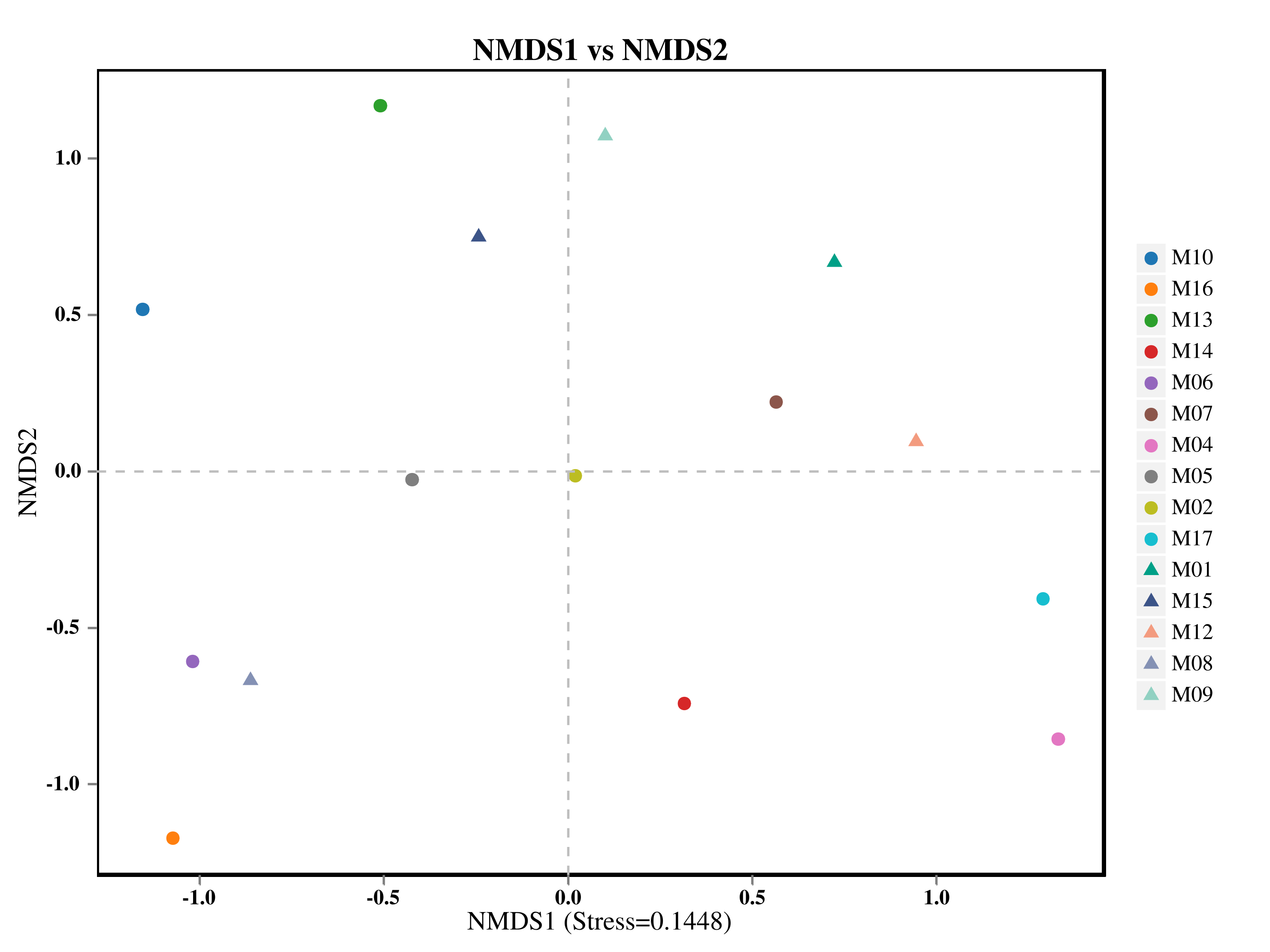

Supplement: Supplementary file 1 — customer_backup. [file MBO3-14-e70178-s001.zip › customer_backup/customer_backup/beta_diversity/nmds/treat/treat.bray_curtis.NMDS1_NMDS2.png]

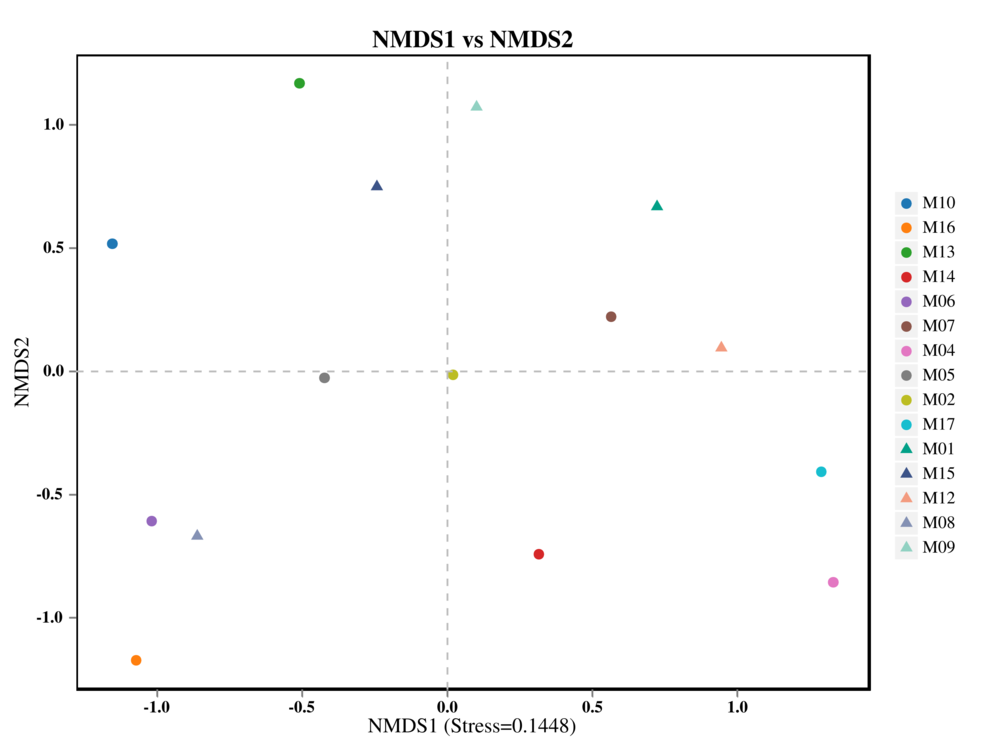

Supplement: Supplementary file 1 — customer_backup. [file MBO3-14-e70178-s001.zip › customer_backup/customer_backup/beta_diversity/nmds/treat/treat.bray_curtis.NMDS1_NMDS2_small.png]

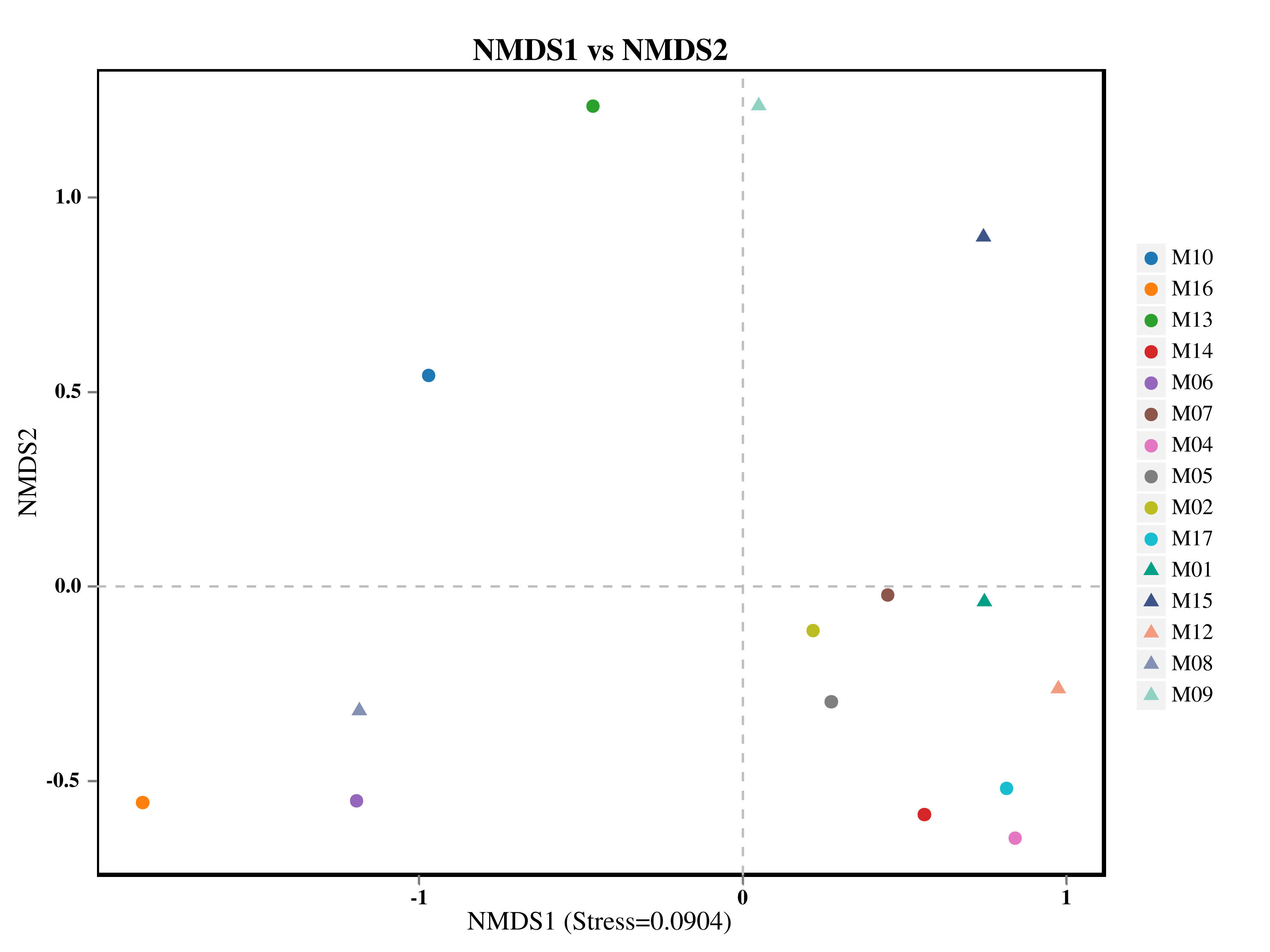

Supplement: Supplementary file 1 — customer_backup. [file MBO3-14-e70178-s001.zip › customer_backup/customer_backup/beta_diversity/nmds/treat/treat.unweighted_unifrac.NMDS1_NMDS2.png]

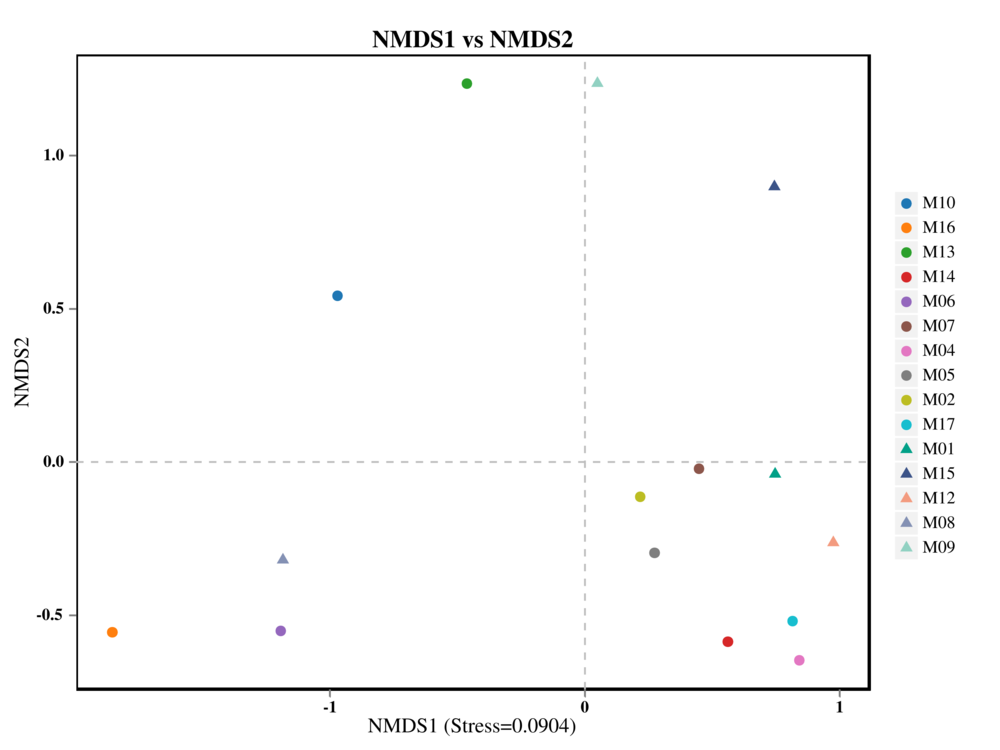

Supplement: Supplementary file 1 — customer_backup. [file MBO3-14-e70178-s001.zip › customer_backup/customer_backup/beta_diversity/nmds/treat/treat.unweighted_unifrac.NMDS1_NMDS2_small.png]

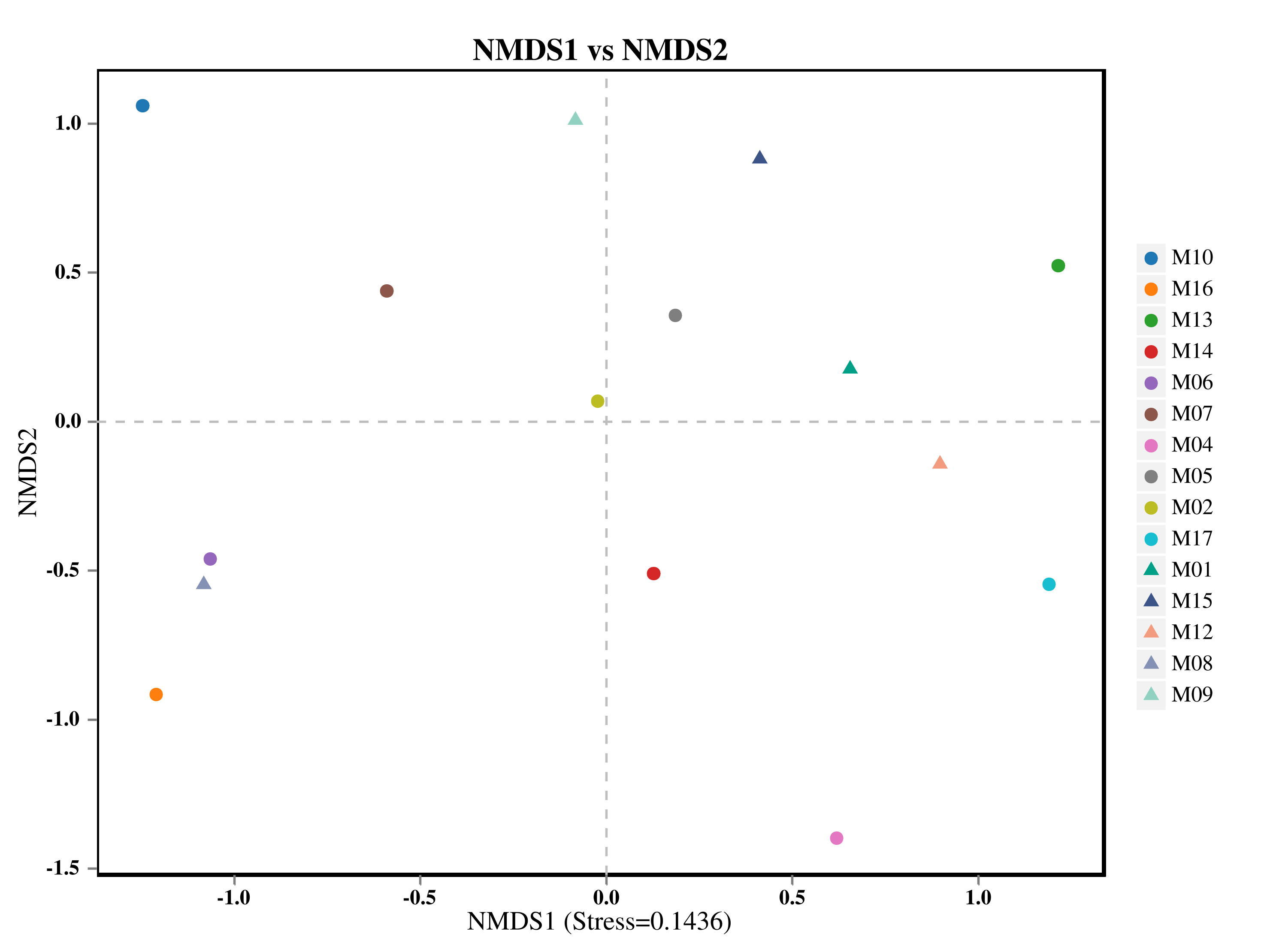

Supplement: Supplementary file 1 — customer_backup. [file MBO3-14-e70178-s001.zip › customer_backup/customer_backup/beta_diversity/nmds/treat/treat.weighted_unifrac.NMDS1_NMDS2.png]

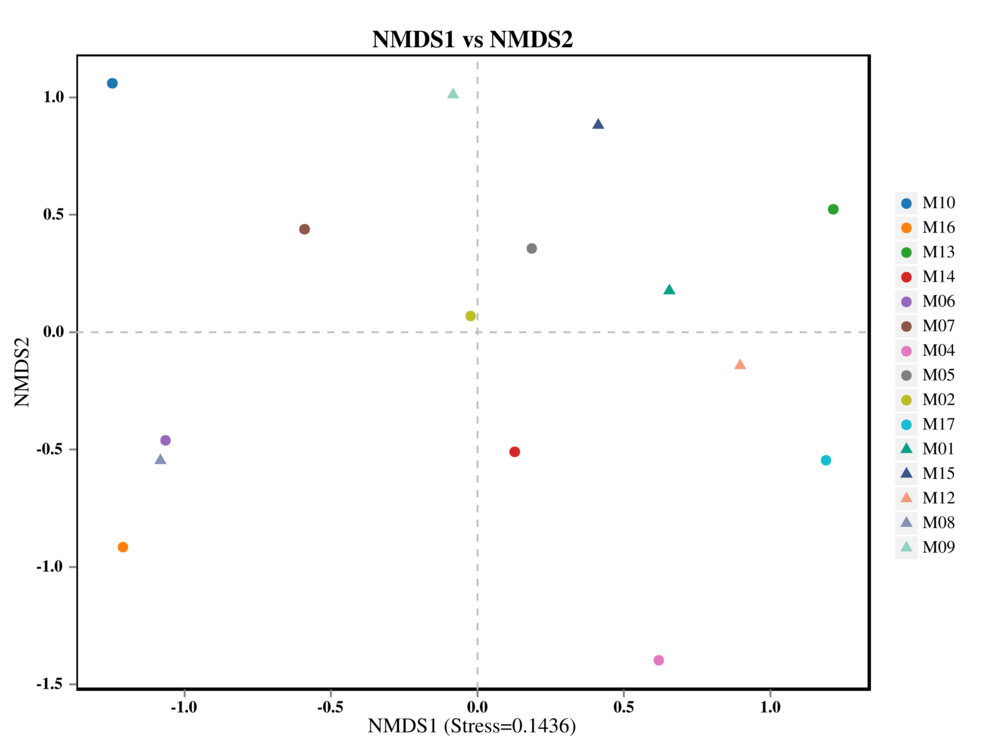

Supplement: Supplementary file 1 — customer_backup. [file MBO3-14-e70178-s001.zip › customer_backup/customer_backup/beta_diversity/nmds/treat/treat.weighted_unifrac.NMDS1_NMDS2_small.png]

PCA – PC1 vs PC2

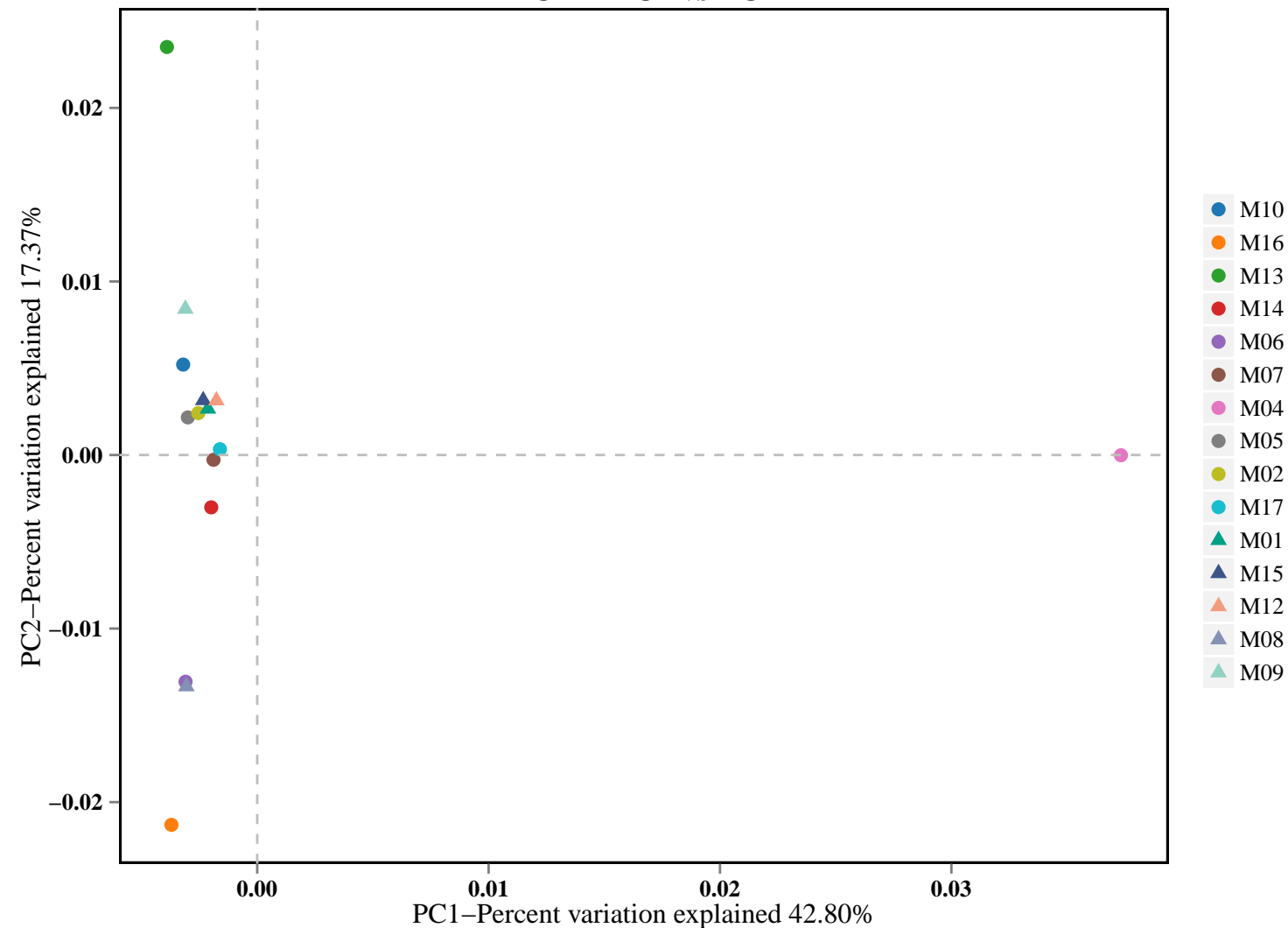

Supplement: Supplementary file 1 — customer_backup. [file MBO3-14-e70178-s001.zip › customer_backup/customer_backup/beta_diversity/pca/treat/PCA.treat.PC1_PC2.pdf]

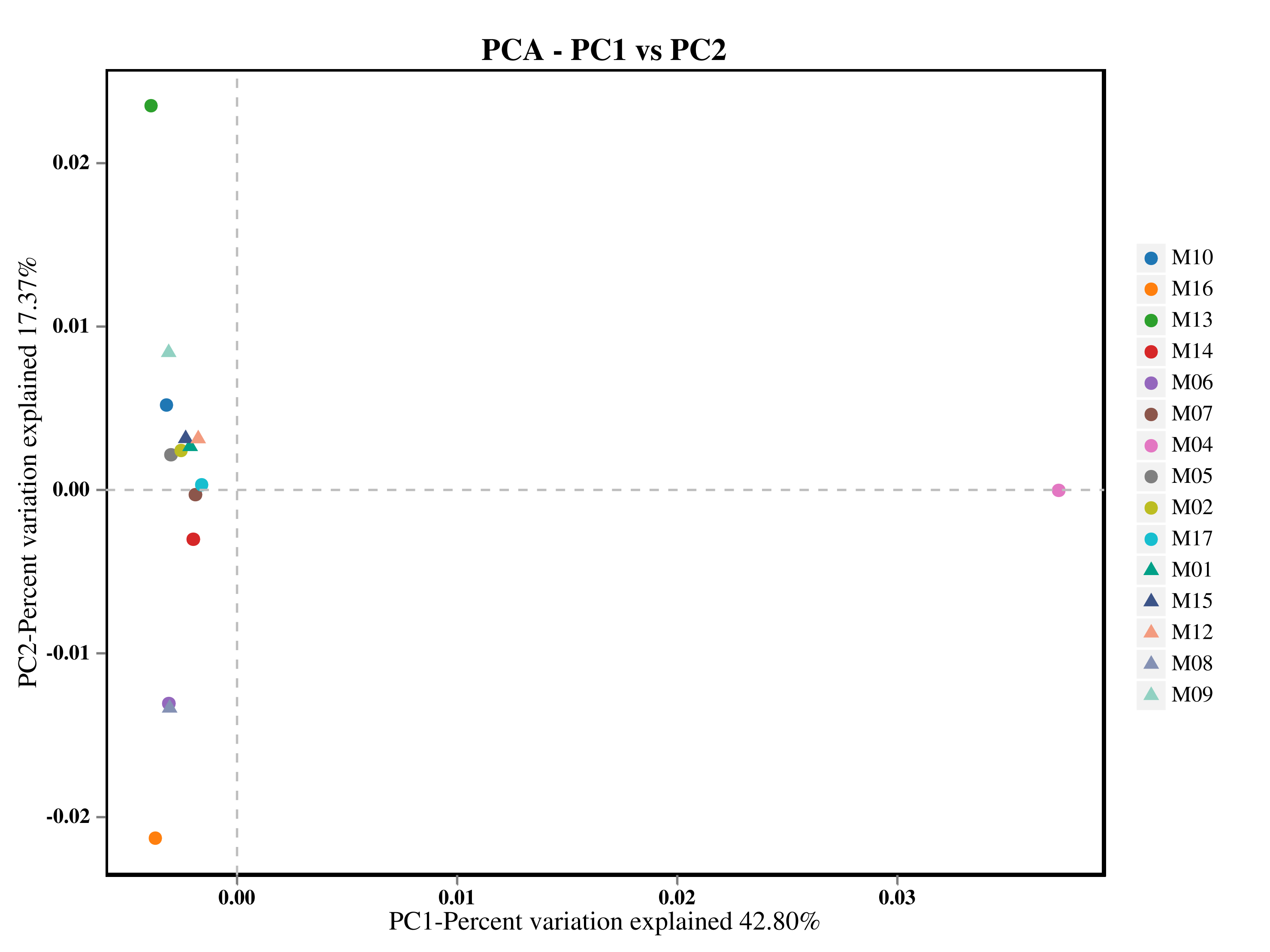

Supplement: Supplementary file 1 — customer_backup. [file MBO3-14-e70178-s001.zip › customer_backup/customer_backup/beta_diversity/pca/treat/PCA.treat.PC1_PC2.png]

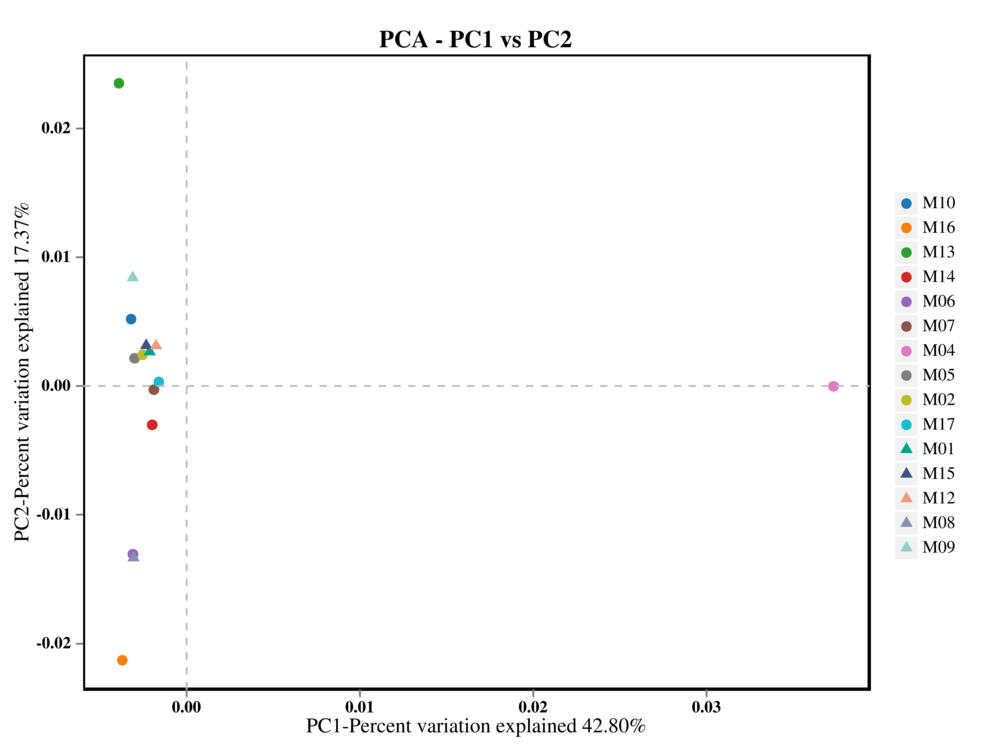

Supplement: Supplementary file 1 — customer_backup. [file MBO3-14-e70178-s001.zip › customer_backup/customer_backup/beta_diversity/pca/treat/PCA.treat.PC1_PC2_small.png]

# PCA – PC1 vs PC3

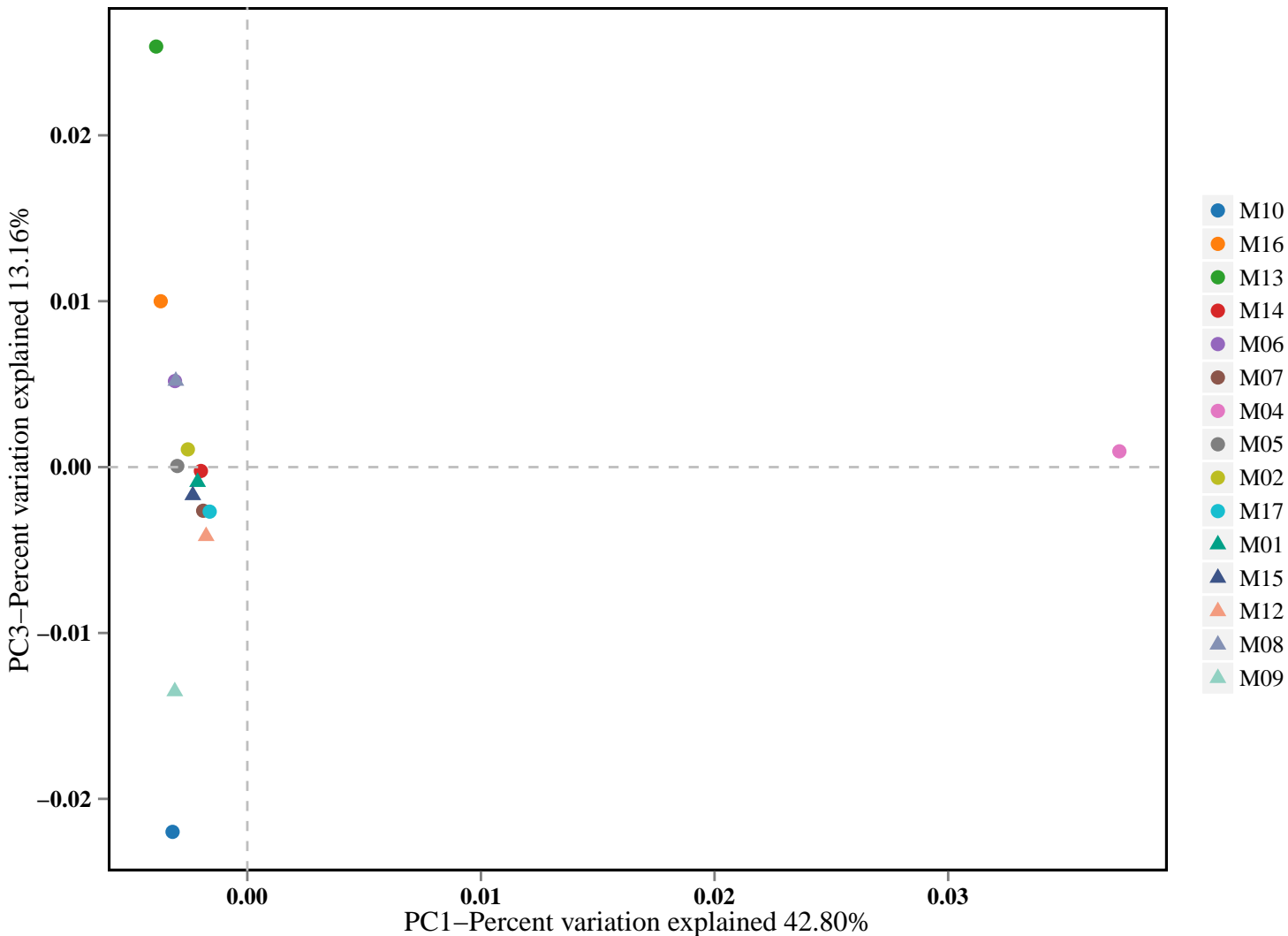

Supplement: Supplementary file 1 — customer_backup. [file MBO3-14-e70178-s001.zip › customer_backup/customer_backup/beta_diversity/pca/treat/PCA.treat.PC1_PC3.pdf]

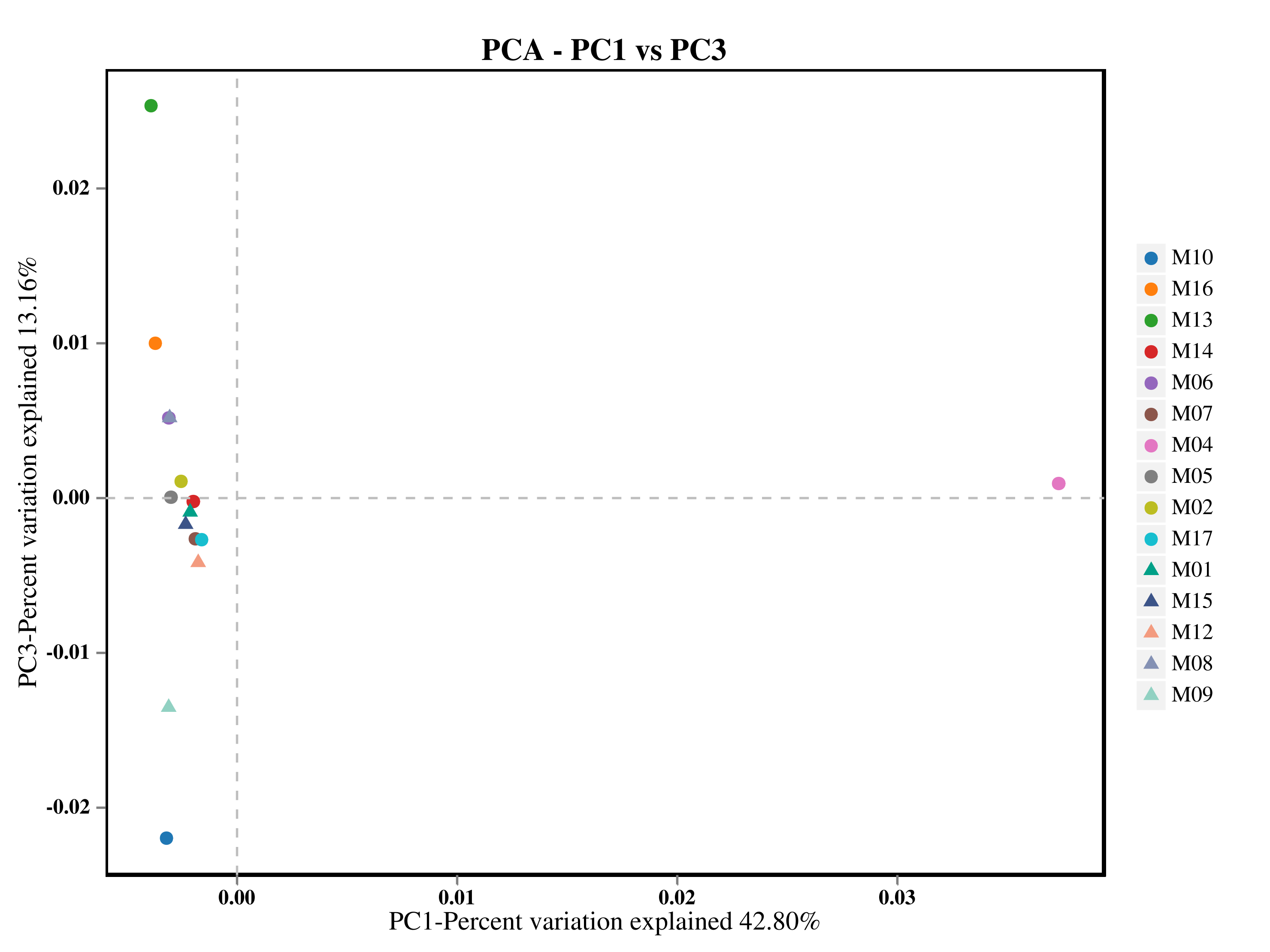

Supplement: Supplementary file 1 — customer_backup. [file MBO3-14-e70178-s001.zip › customer_backup/customer_backup/beta_diversity/pca/treat/PCA.treat.PC1_PC3.png]

PCA – PC2 vs PC3

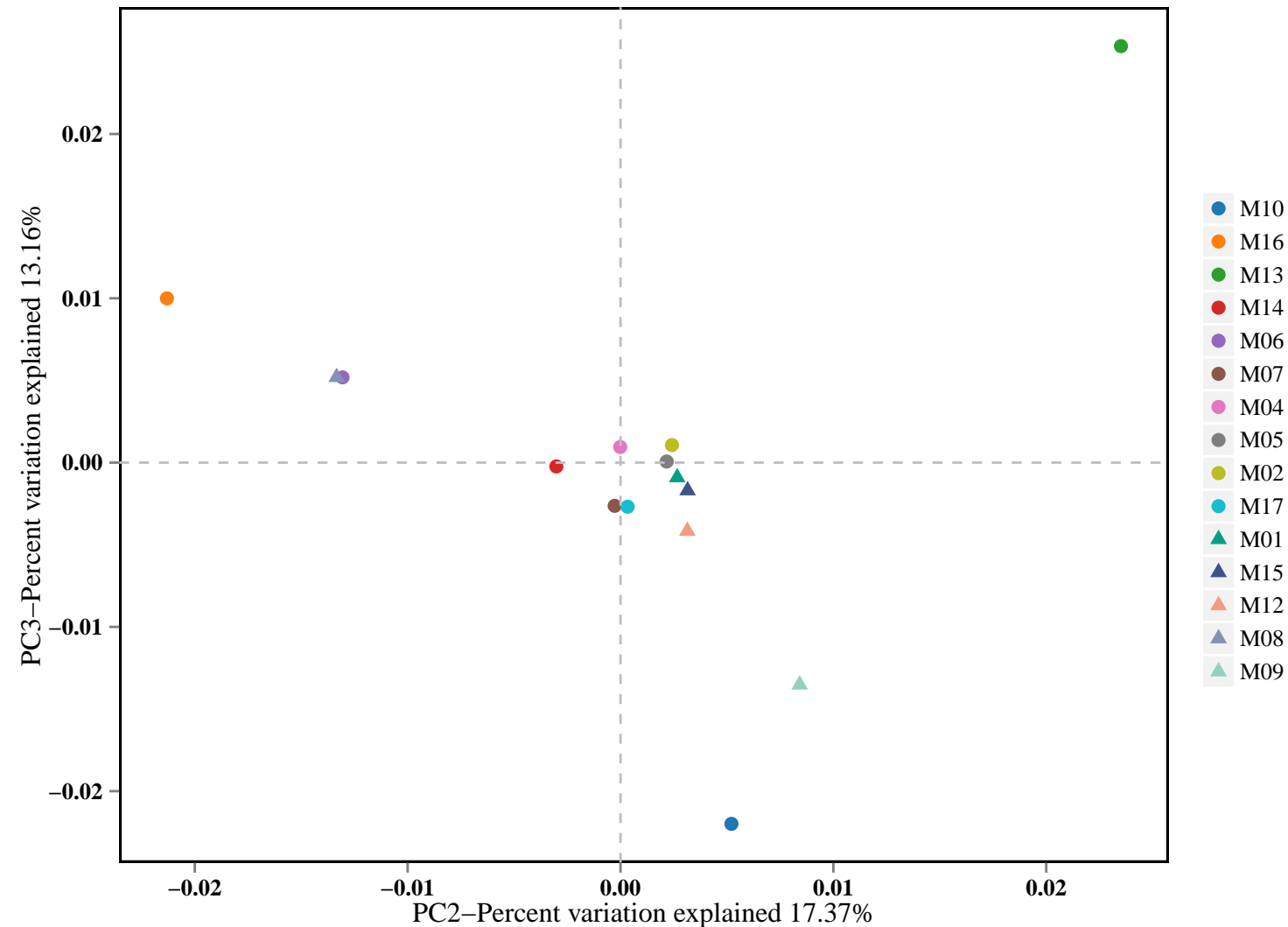

Supplement: Supplementary file 1 — customer_backup. [file MBO3-14-e70178-s001.zip › customer_backup/customer_backup/beta_diversity/pca/treat/PCA.treat.PC2_PC3.pdf]

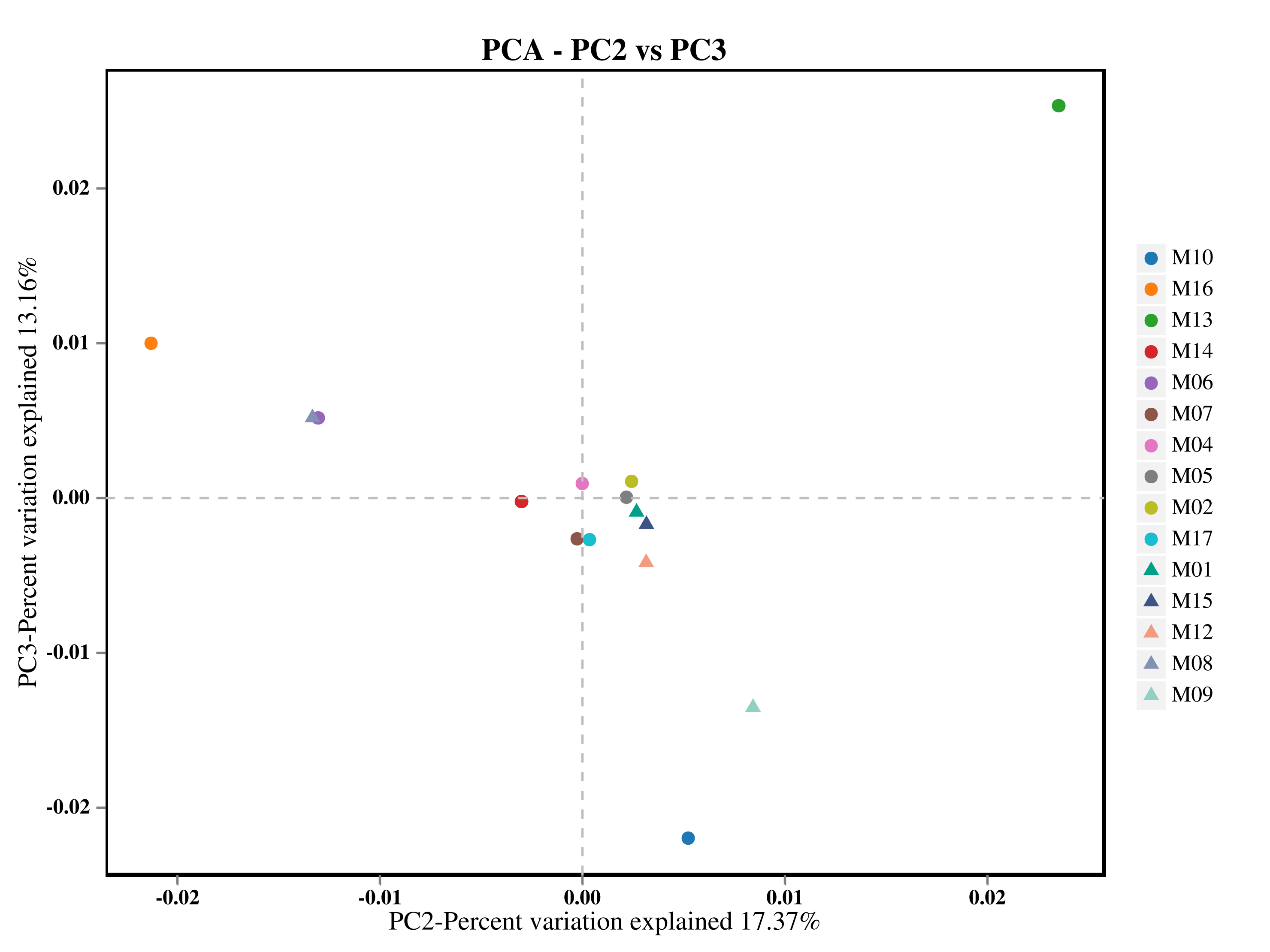

Supplement: Supplementary file 1 — customer_backup. [file MBO3-14-e70178-s001.zip › customer_backup/customer_backup/beta_diversity/pca/treat/PCA.treat.PC2_PC3.png]

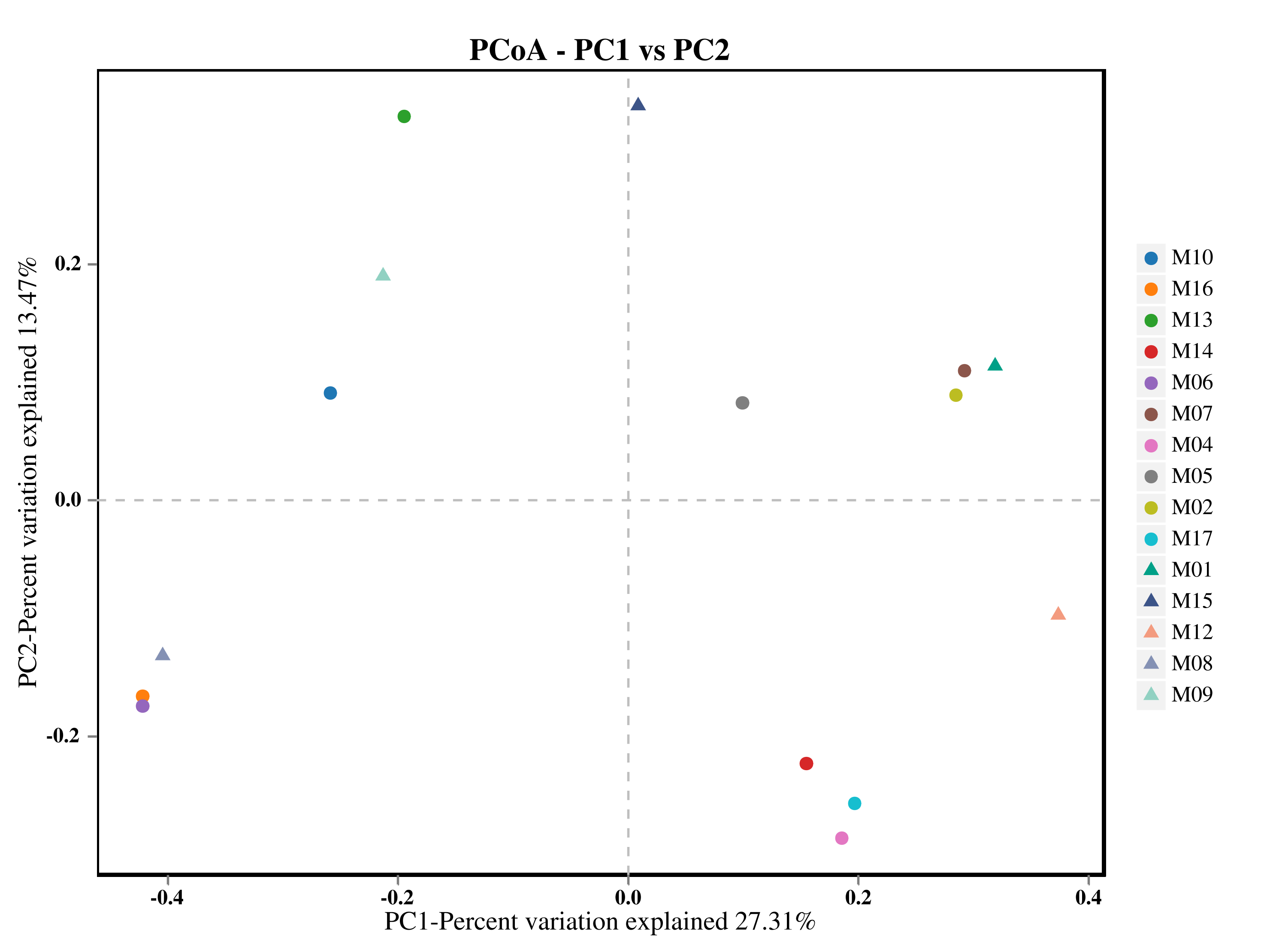

Supplement: Supplementary file 1 — customer_backup. [file MBO3-14-e70178-s001.zip › customer_backup/customer_backup/beta_diversity/pcoa/treat/treat.binary_jaccard.PC1_PC2.png]

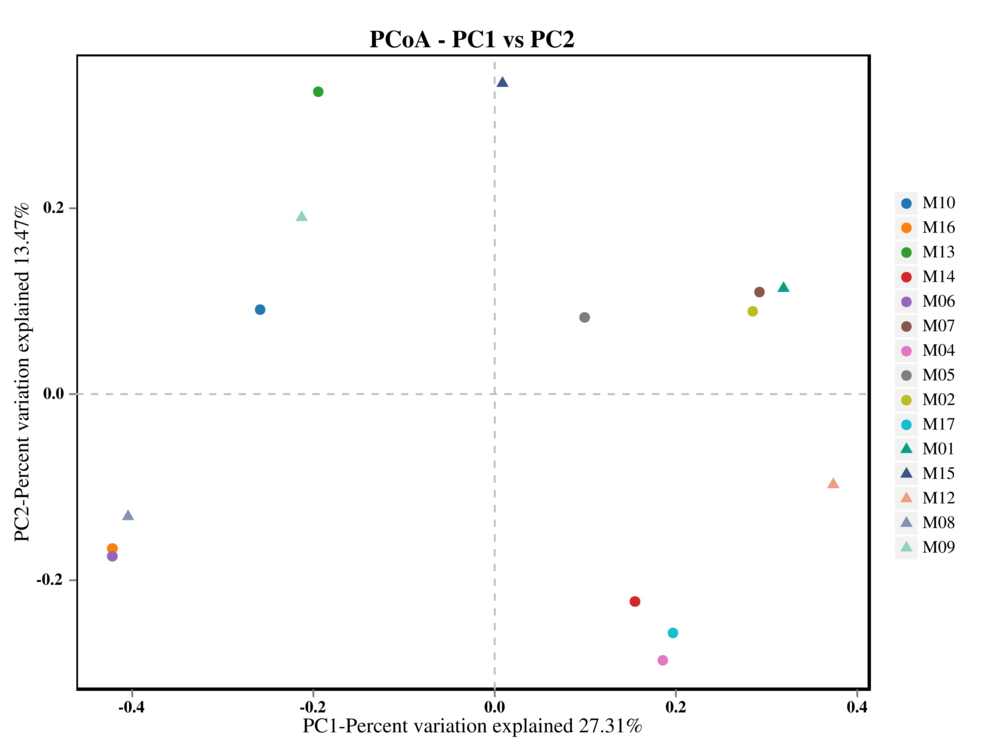

Supplement: Supplementary file 1 — customer_backup. [file MBO3-14-e70178-s001.zip › customer_backup/customer_backup/beta_diversity/pcoa/treat/treat.binary_jaccard.PC1_PC2_small.png]

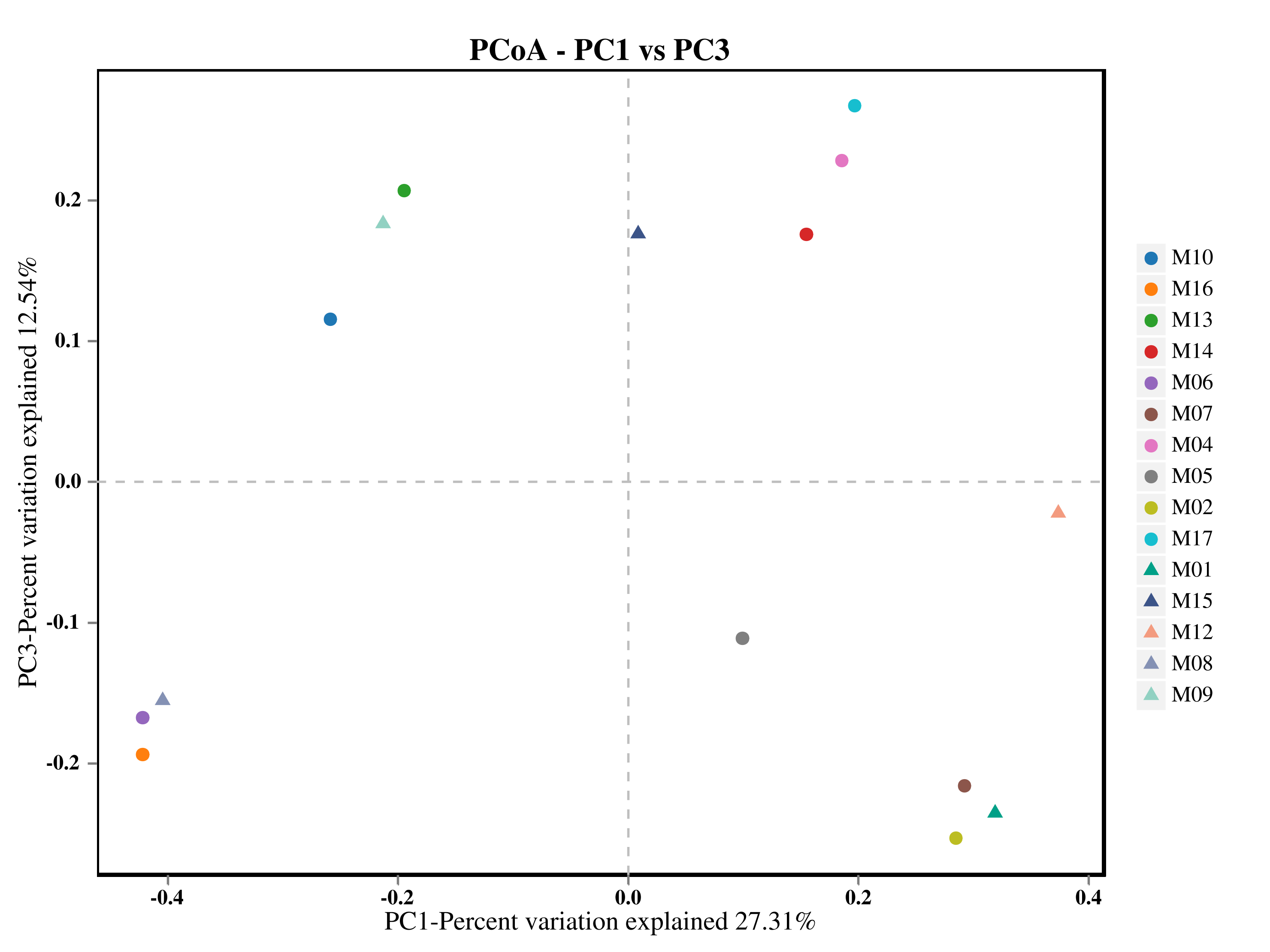

Supplement: Supplementary file 1 — customer_backup. [file MBO3-14-e70178-s001.zip › customer_backup/customer_backup/beta_diversity/pcoa/treat/treat.binary_jaccard.PC1_PC3.png]

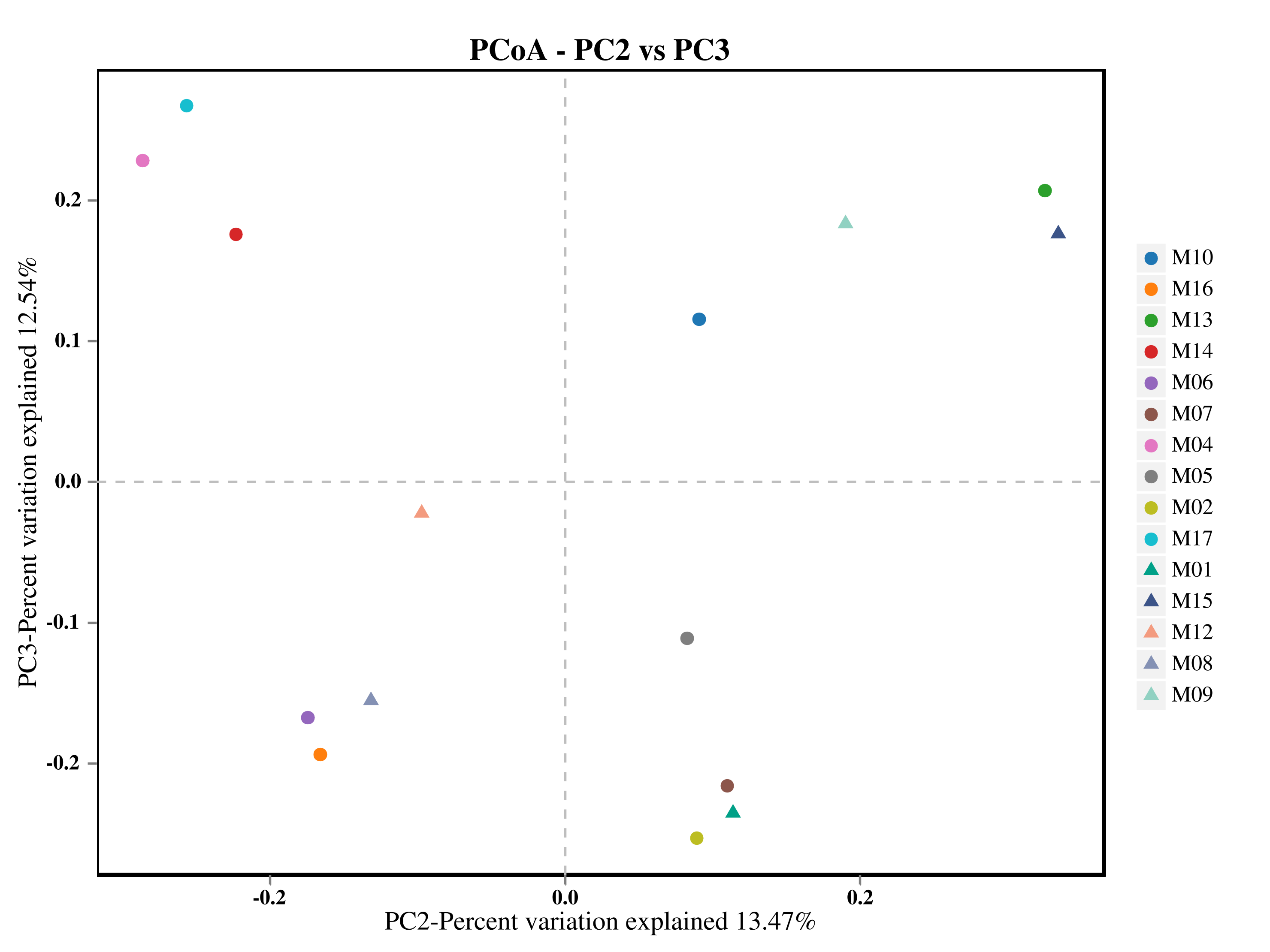

Supplement: Supplementary file 1 — customer_backup. [file MBO3-14-e70178-s001.zip › customer_backup/customer_backup/beta_diversity/pcoa/treat/treat.binary_jaccard.PC2_PC3.png]

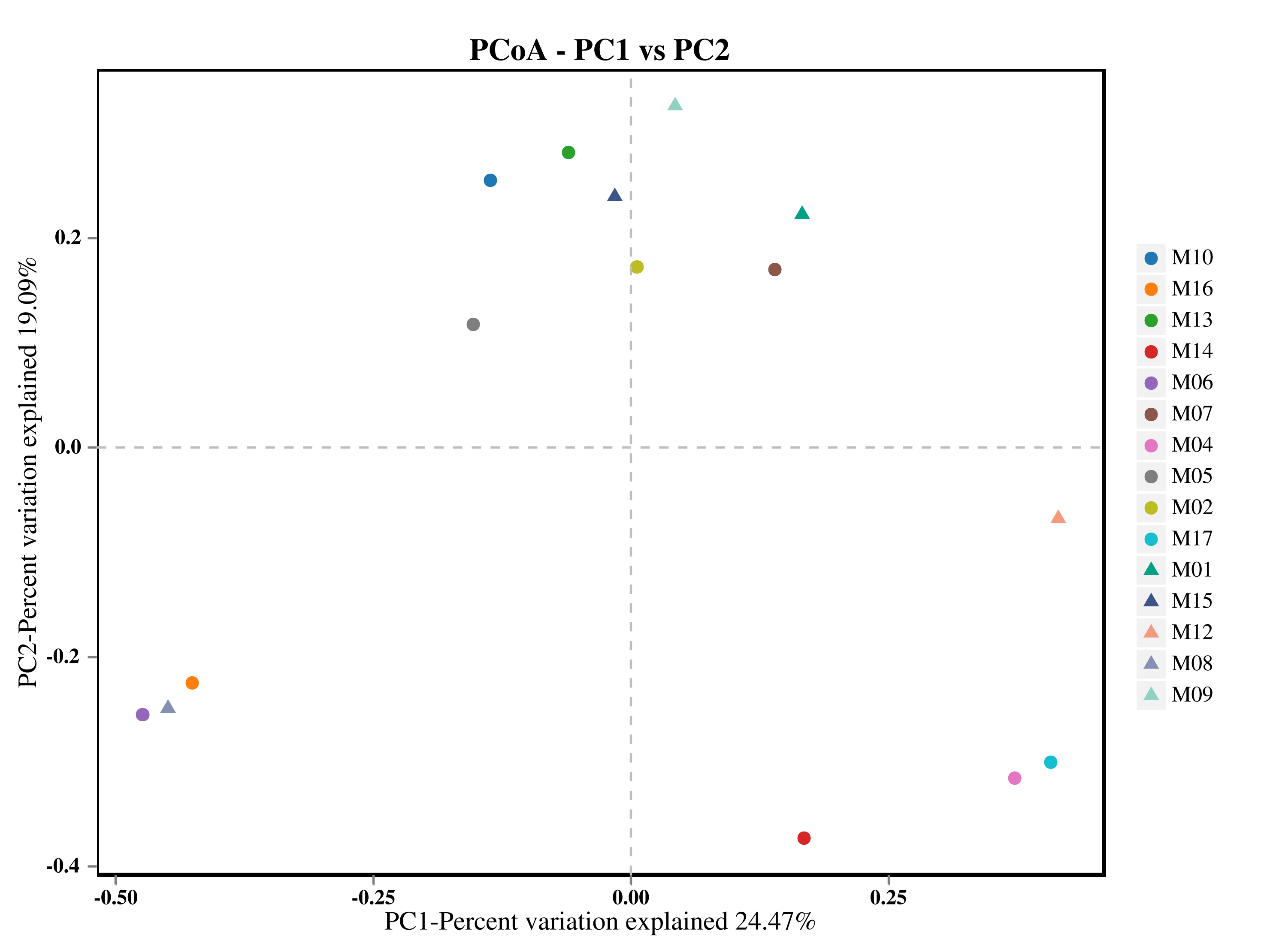

Supplement: Supplementary file 1 — customer_backup. [file MBO3-14-e70178-s001.zip › customer_backup/customer_backup/beta_diversity/pcoa/treat/treat.bray_curtis.PC1_PC2.png]

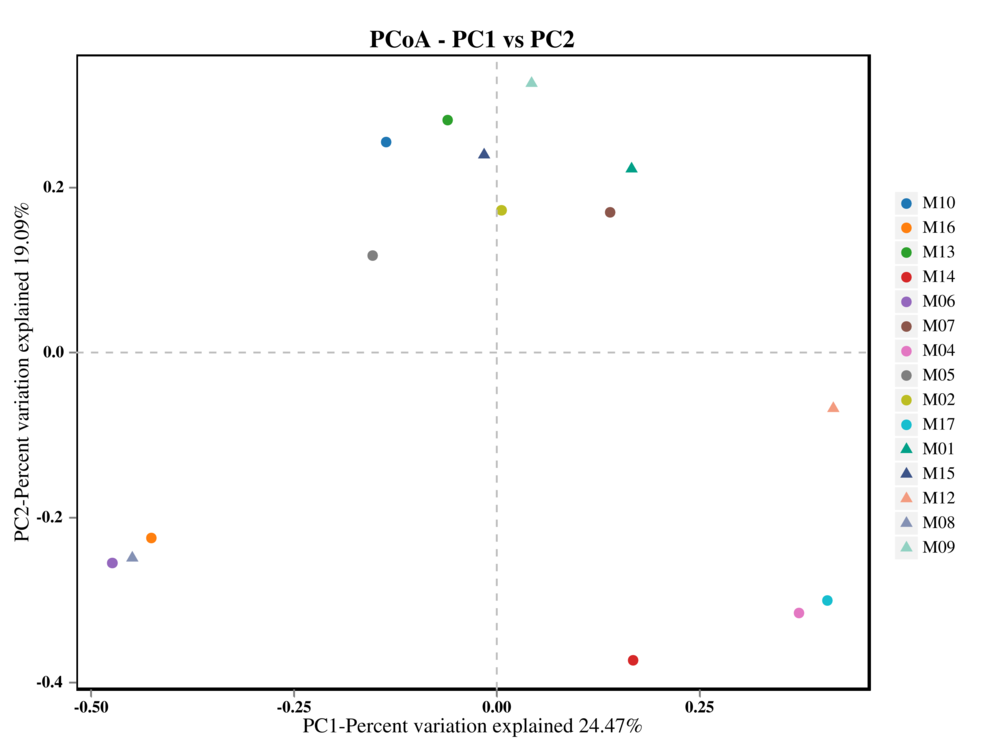

Supplement: Supplementary file 1 — customer_backup. [file MBO3-14-e70178-s001.zip › customer_backup/customer_backup/beta_diversity/pcoa/treat/treat.bray_curtis.PC1_PC2_small.png]

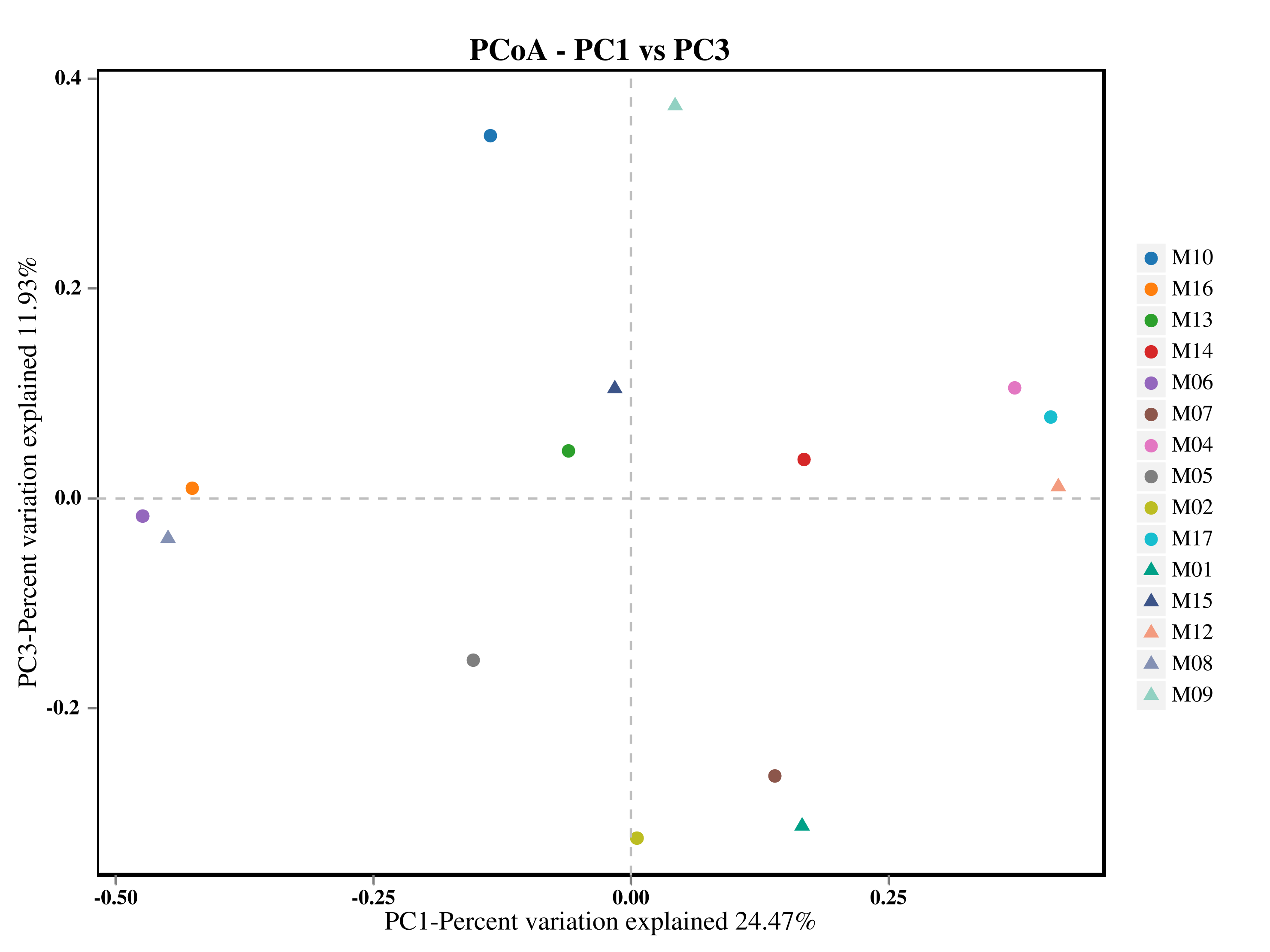

Supplement: Supplementary file 1 — customer_backup. [file MBO3-14-e70178-s001.zip › customer_backup/customer_backup/beta_diversity/pcoa/treat/treat.bray_curtis.PC1_PC3.png]

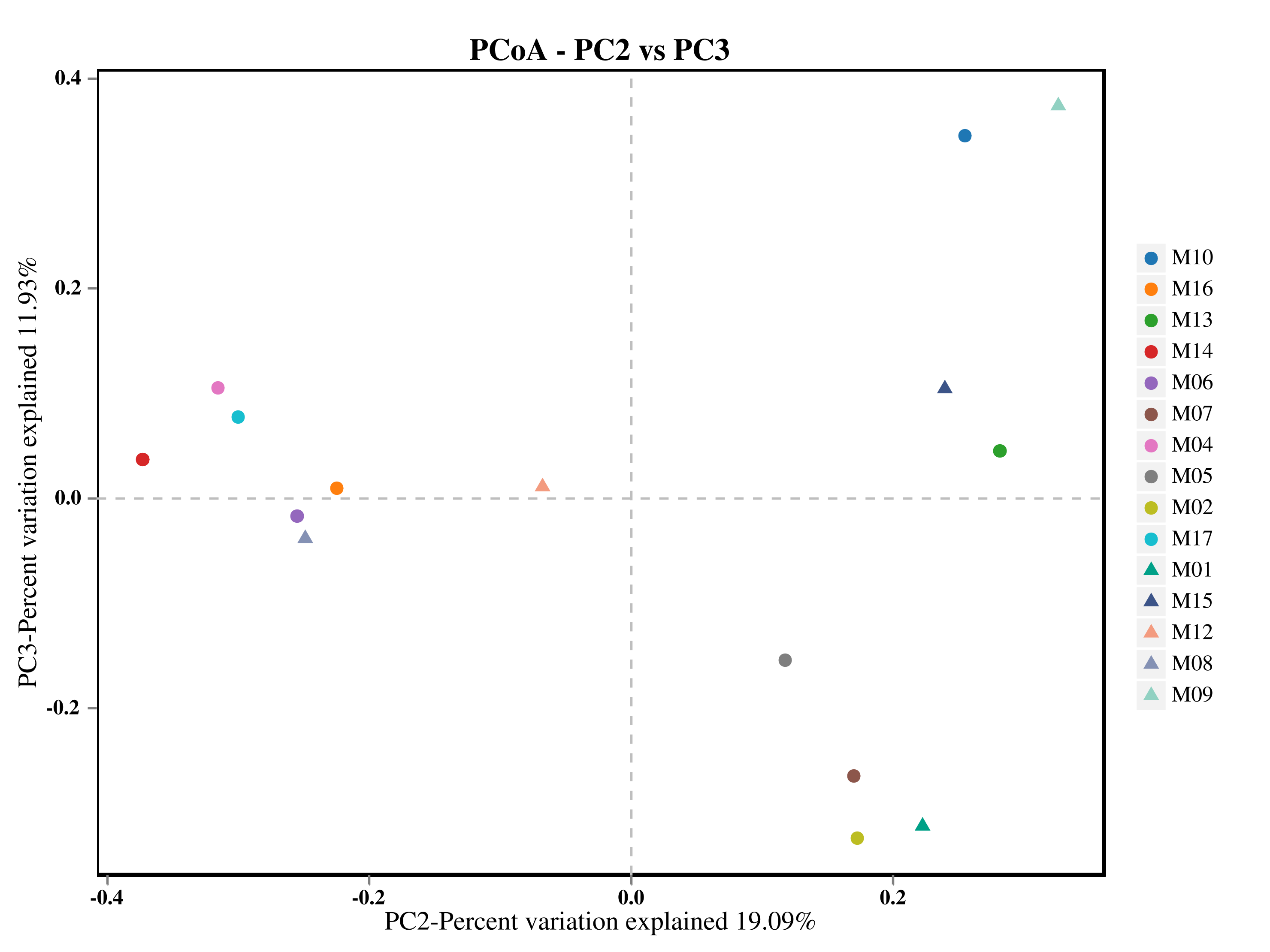

Supplement: Supplementary file 1 — customer_backup. [file MBO3-14-e70178-s001.zip › customer_backup/customer_backup/beta_diversity/pcoa/treat/treat.bray_curtis.PC2_PC3.png]

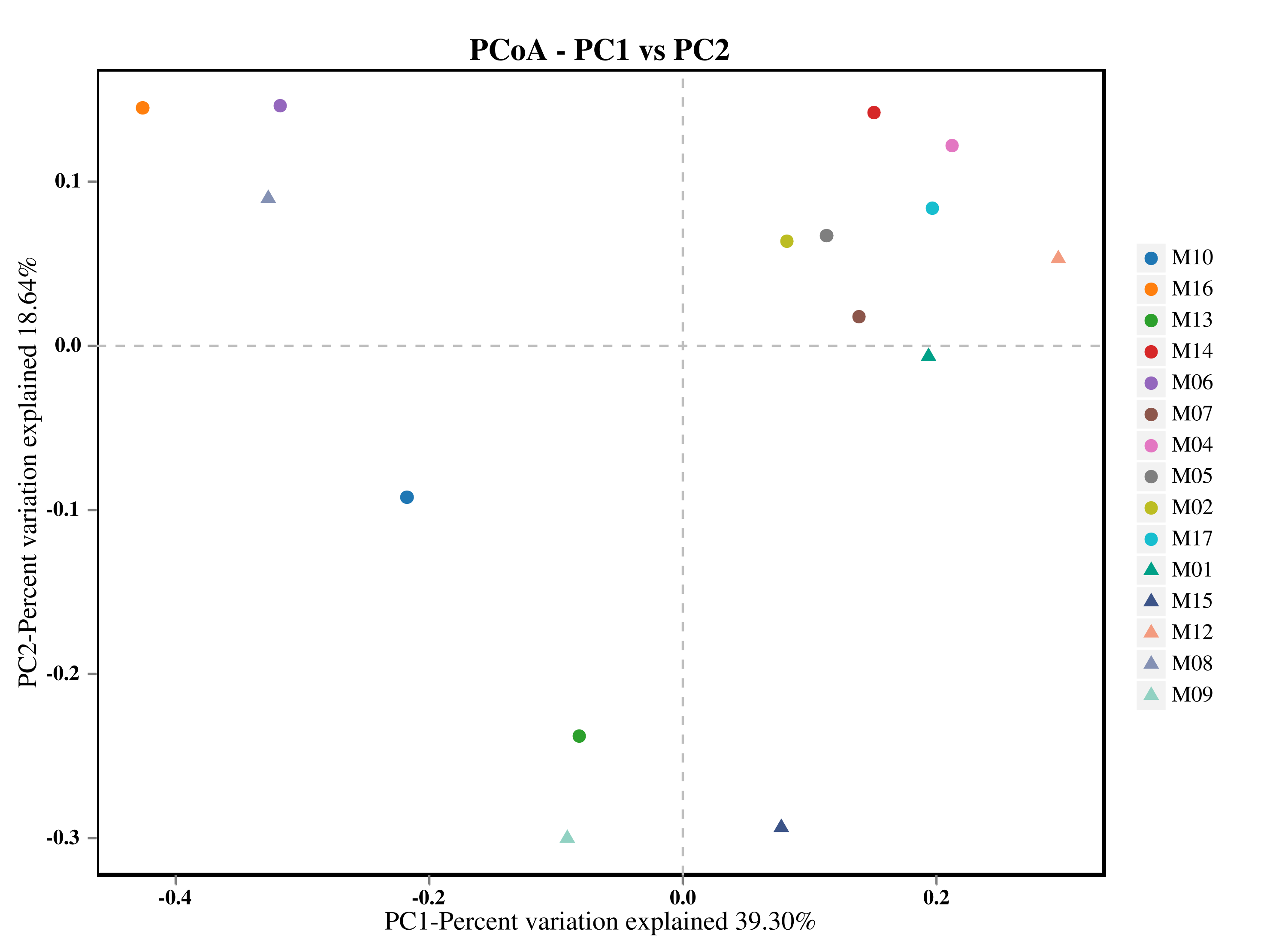

Supplement: Supplementary file 1 — customer_backup. [file MBO3-14-e70178-s001.zip › customer_backup/customer_backup/beta_diversity/pcoa/treat/treat.unweighted_unifrac.PC1_PC2.png]

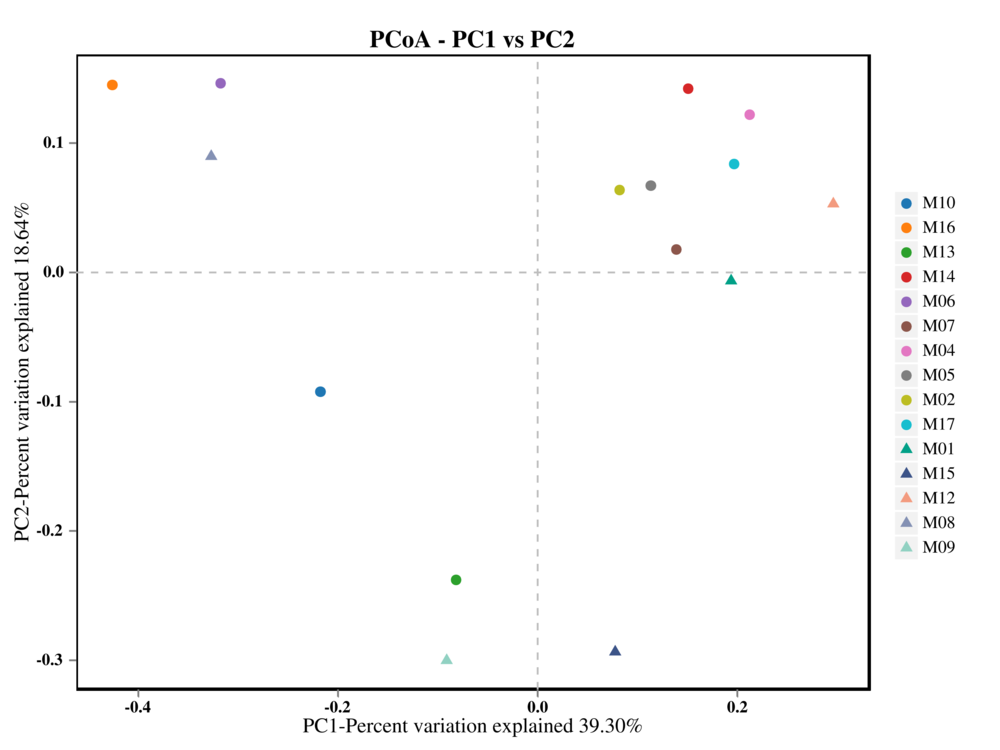

Supplement: Supplementary file 1 — customer_backup. [file MBO3-14-e70178-s001.zip › customer_backup/customer_backup/beta_diversity/pcoa/treat/treat.unweighted_unifrac.PC1_PC2_small.png]

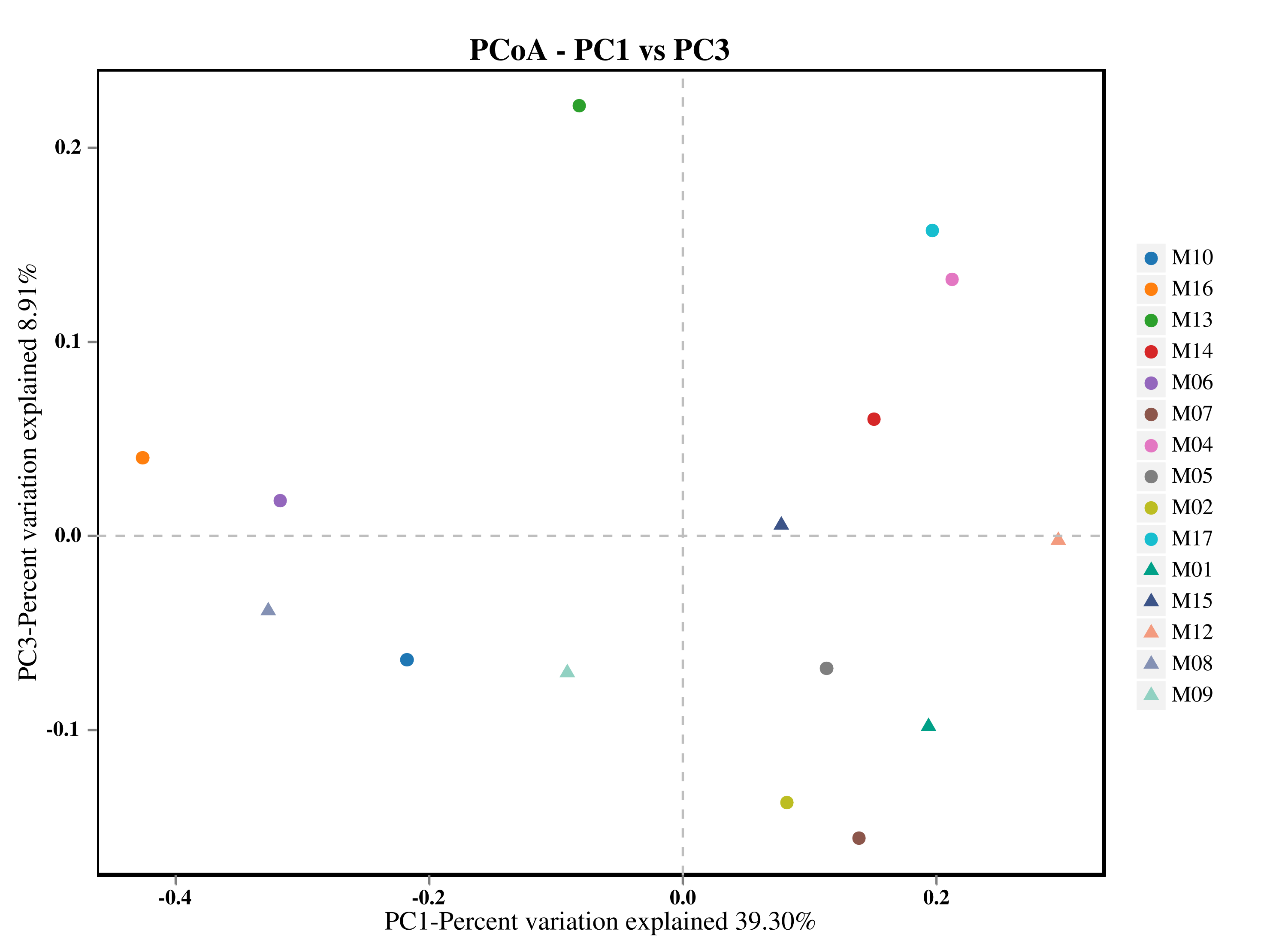

Supplement: Supplementary file 1 — customer_backup. [file MBO3-14-e70178-s001.zip › customer_backup/customer_backup/beta_diversity/pcoa/treat/treat.unweighted_unifrac.PC1_PC3.png]

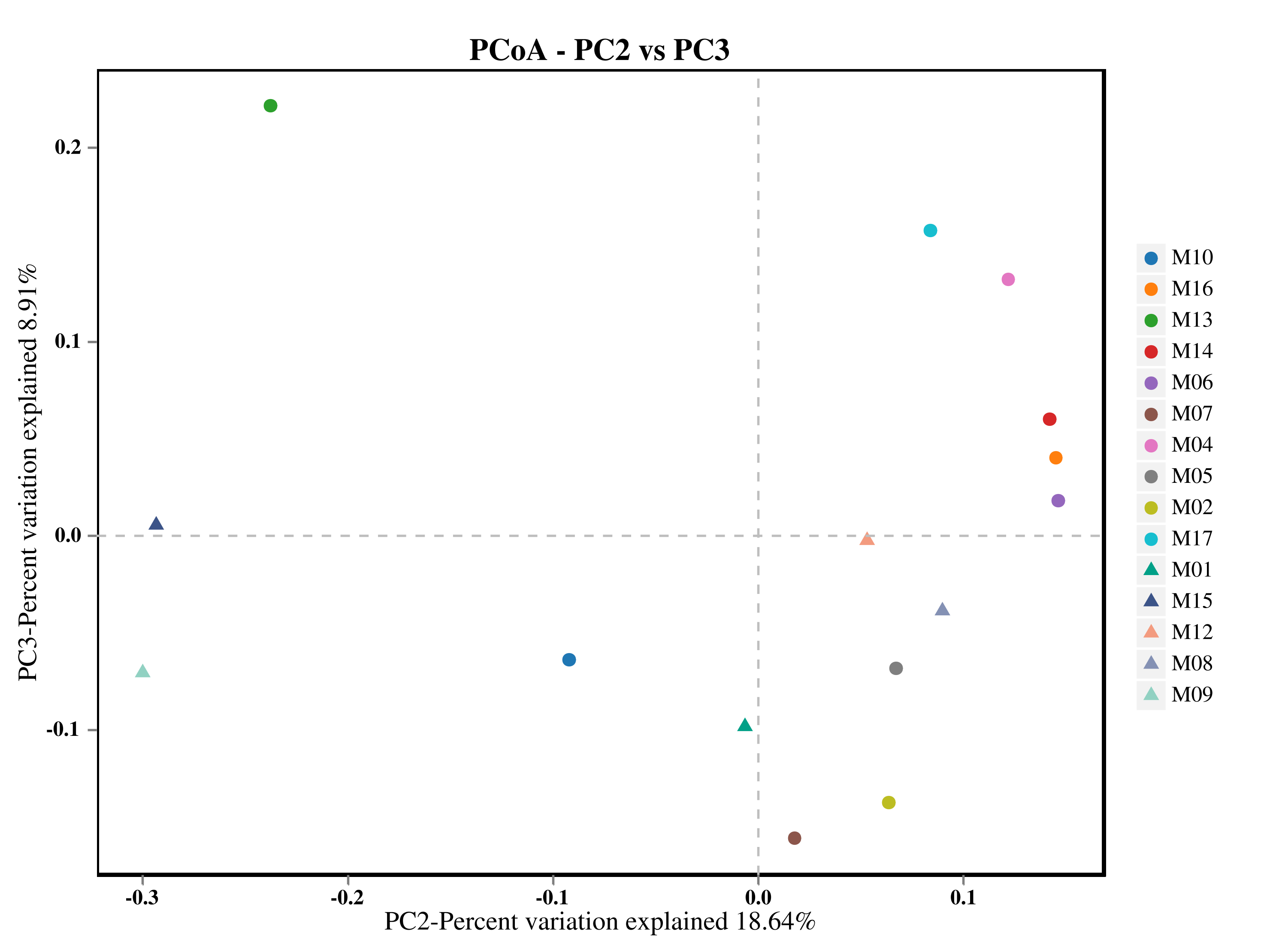

Supplement: Supplementary file 1 — customer_backup. [file MBO3-14-e70178-s001.zip › customer_backup/customer_backup/beta_diversity/pcoa/treat/treat.unweighted_unifrac.PC2_PC3.png]

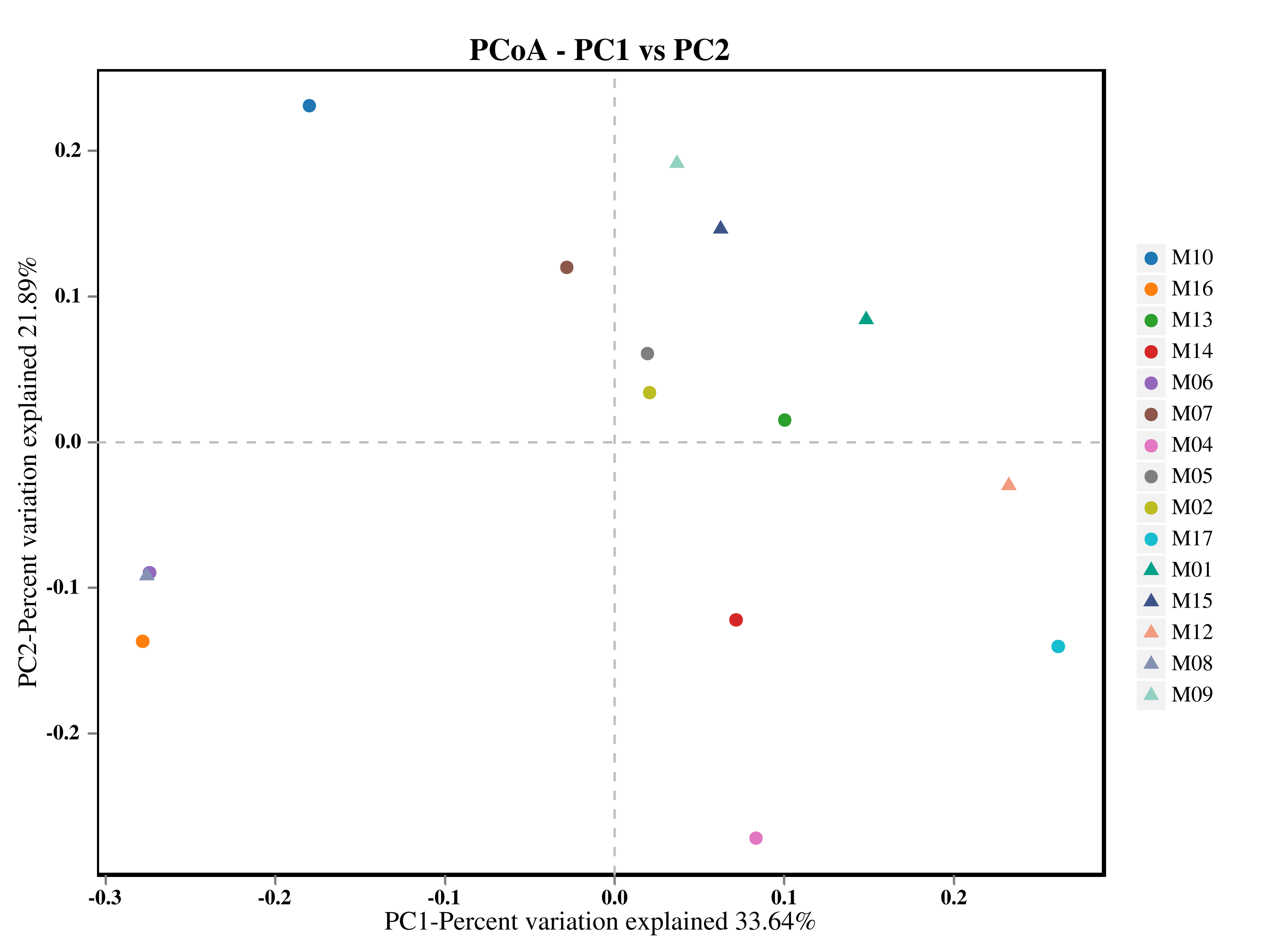

Supplement: Supplementary file 1 — customer_backup. [file MBO3-14-e70178-s001.zip › customer_backup/customer_backup/beta_diversity/pcoa/treat/treat.weighted_unifrac.PC1_PC2.png]

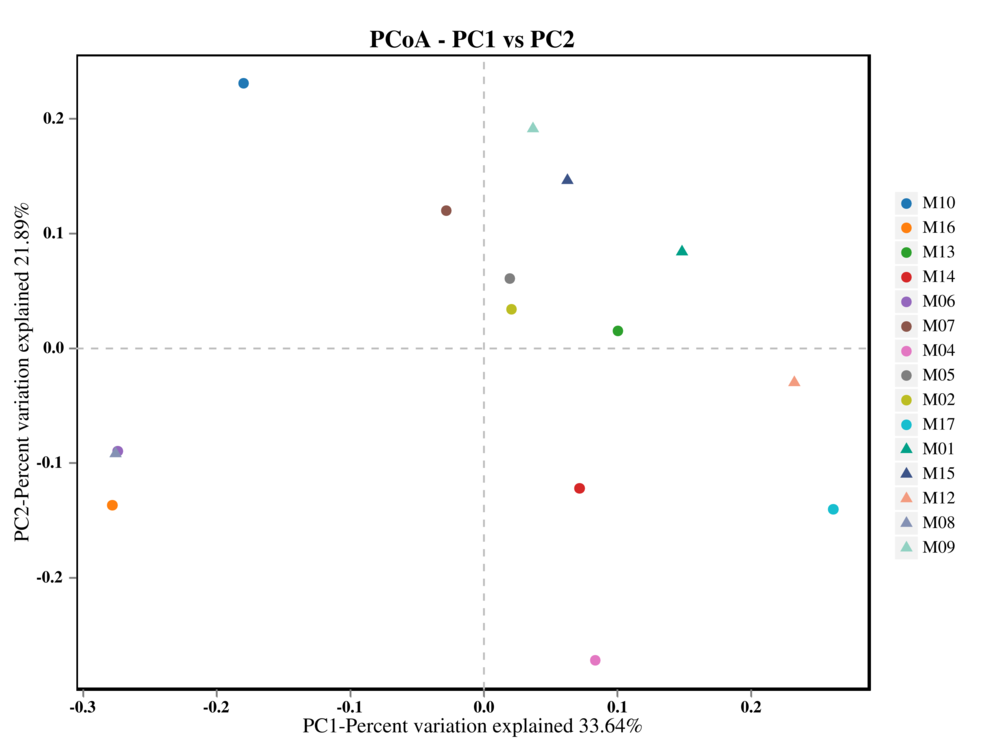

Supplement: Supplementary file 1 — customer_backup. [file MBO3-14-e70178-s001.zip › customer_backup/customer_backup/beta_diversity/pcoa/treat/treat.weighted_unifrac.PC1_PC2_small.png]

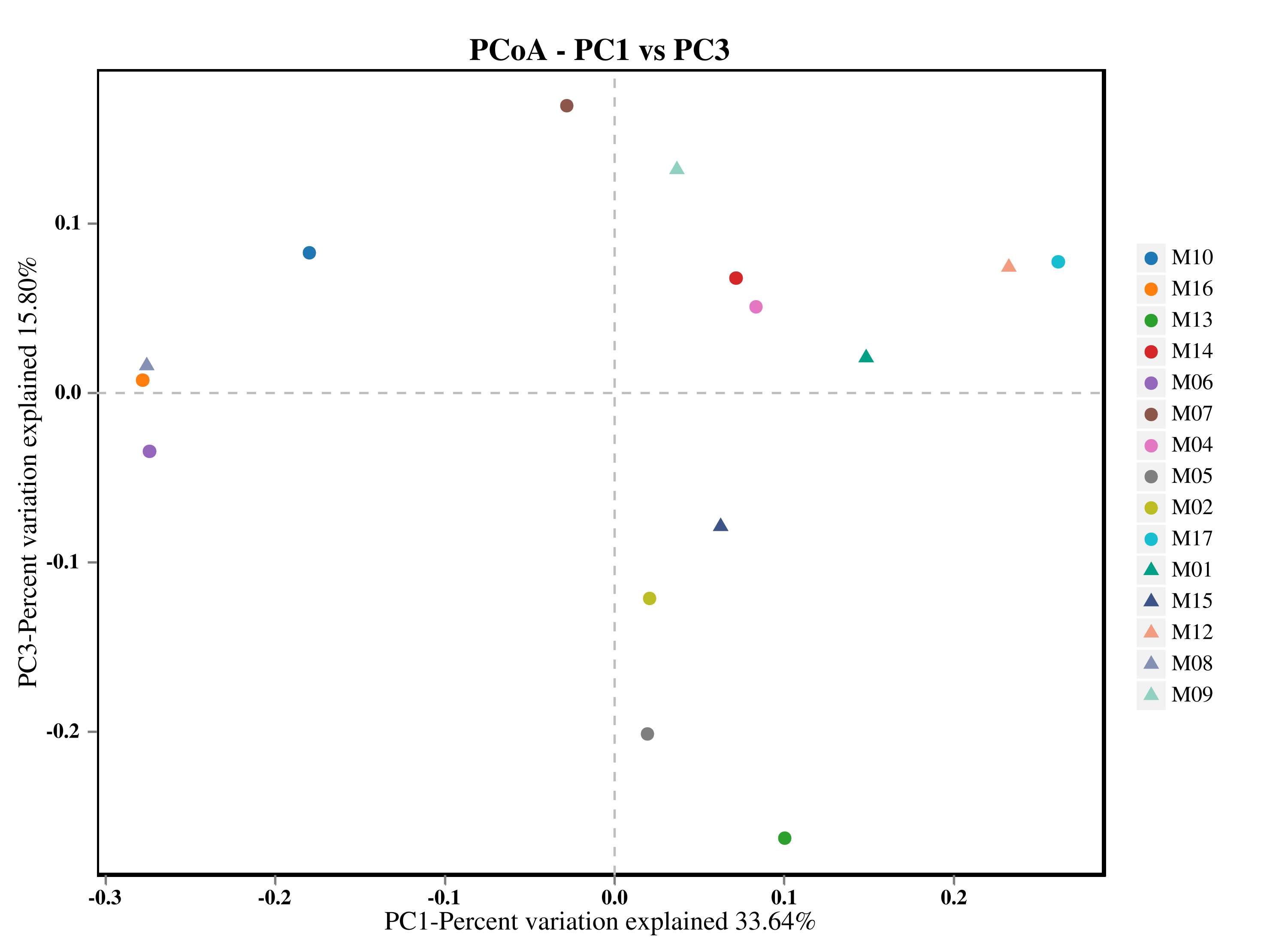

Supplement: Supplementary file 1 — customer_backup. [file MBO3-14-e70178-s001.zip › customer_backup/customer_backup/beta_diversity/pcoa/treat/treat.weighted_unifrac.PC1_PC3.png]

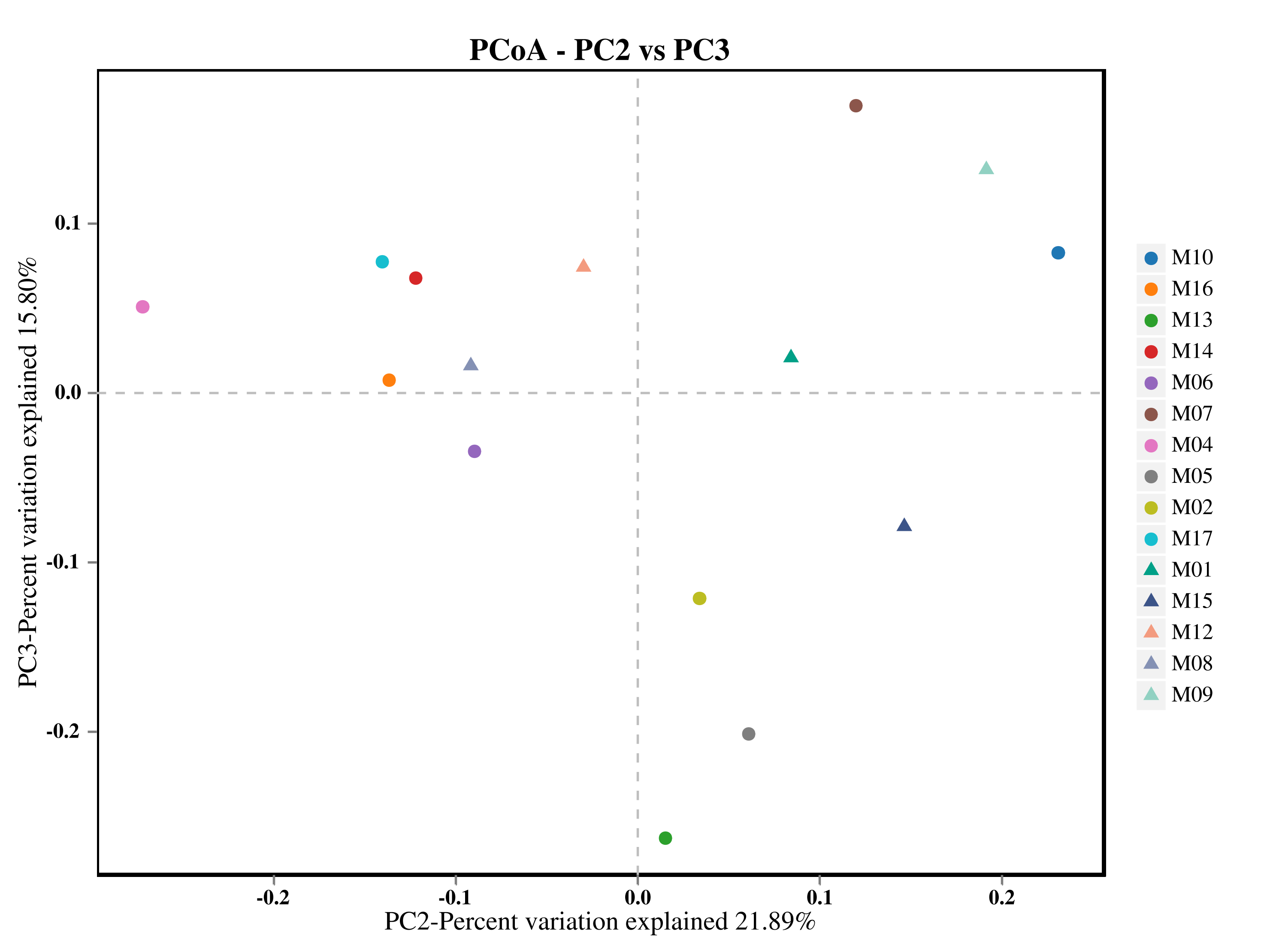

Supplement: Supplementary file 1 — customer_backup. [file MBO3-14-e70178-s001.zip › customer_backup/customer_backup/beta_diversity/pcoa/treat/treat.weighted_unifrac.PC2_PC3.png]

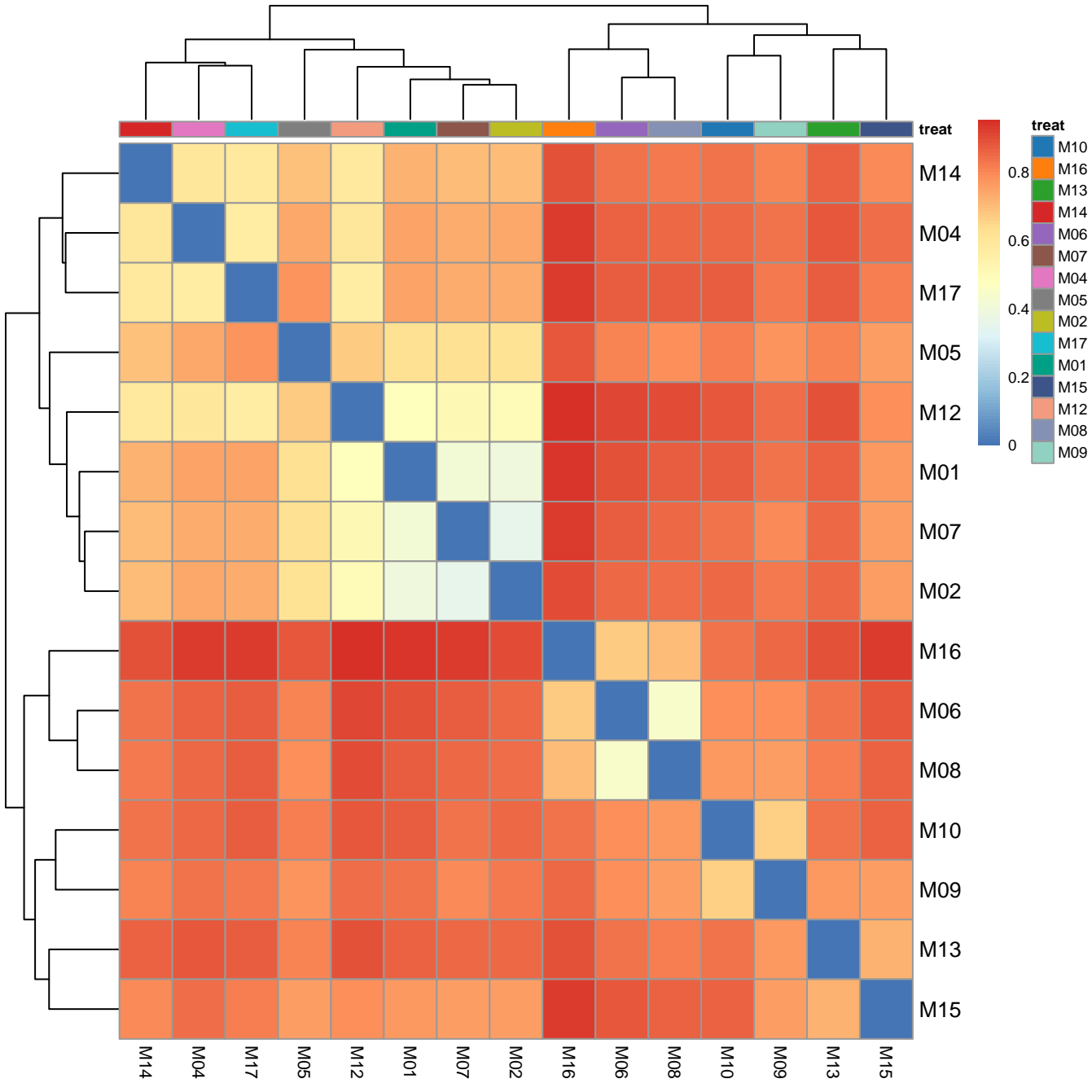

Supplement: Supplementary file 1 — customer_backup. [file MBO3-14-e70178-s001.zip › customer_backup/customer_backup/beta_diversity/sample_heatmap/treat/treat.binary_jaccard_dm.heatmap.pdf]

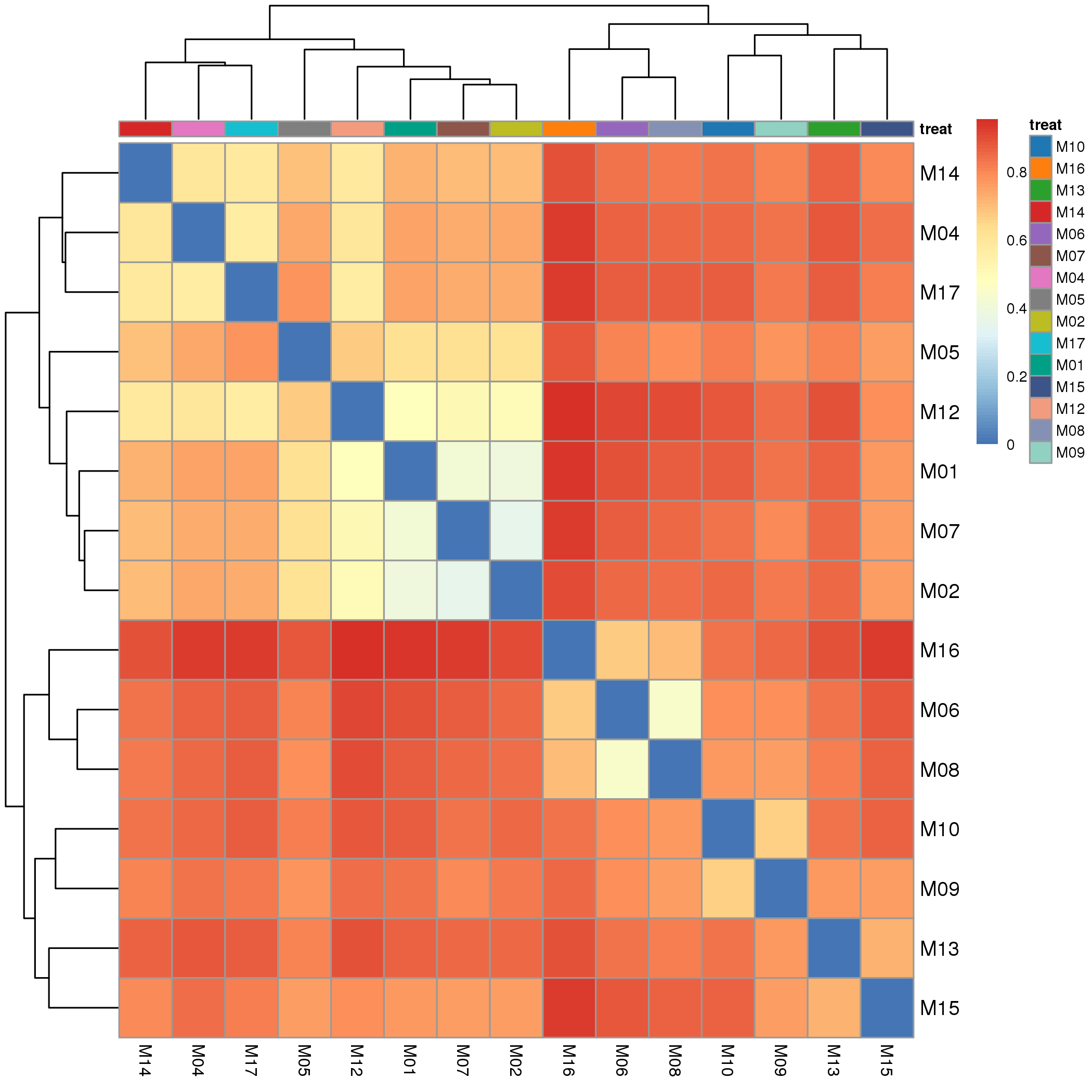

Supplement: Supplementary file 1 — customer_backup. [file MBO3-14-e70178-s001.zip › customer_backup/customer_backup/beta_diversity/sample_heatmap/treat/treat.binary_jaccard_dm.heatmap.png]

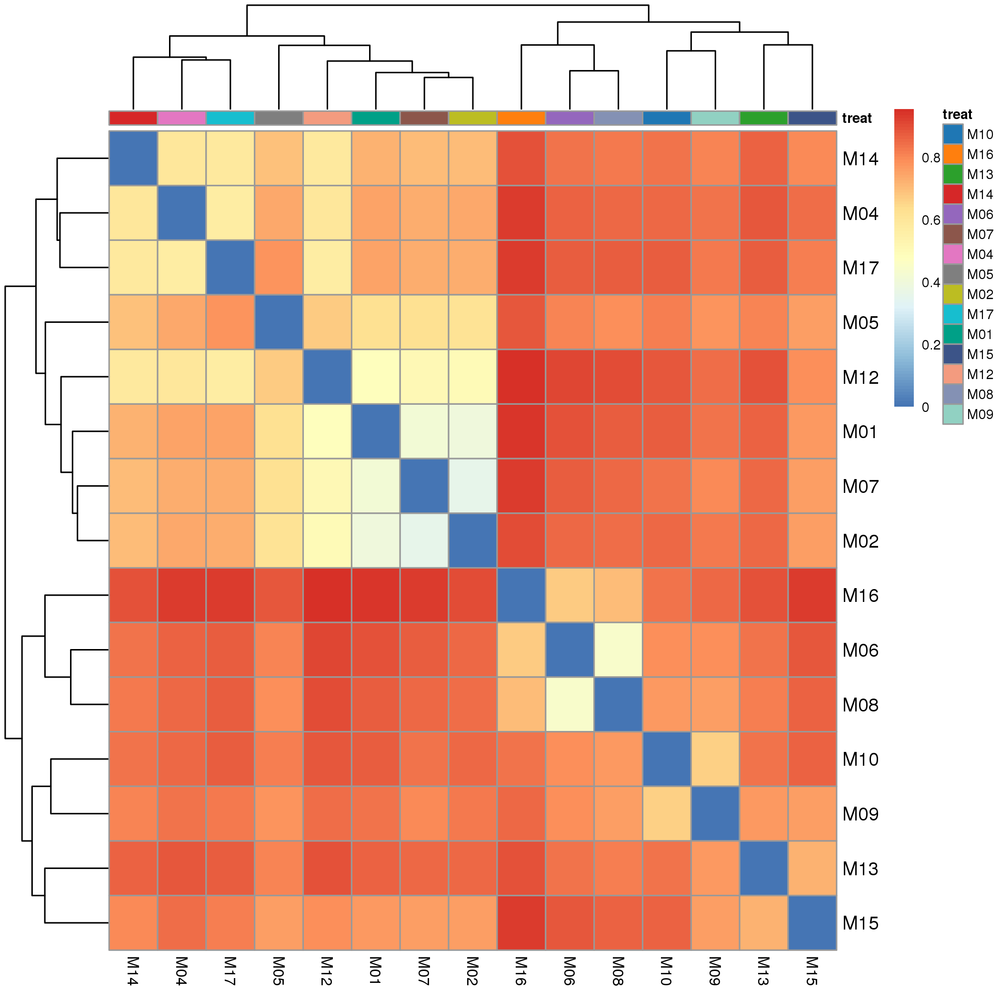

Supplement: Supplementary file 1 — customer_backup. [file MBO3-14-e70178-s001.zip › customer_backup/customer_backup/beta_diversity/sample_heatmap/treat/treat.binary_jaccard_dm.heatmap_small.png]

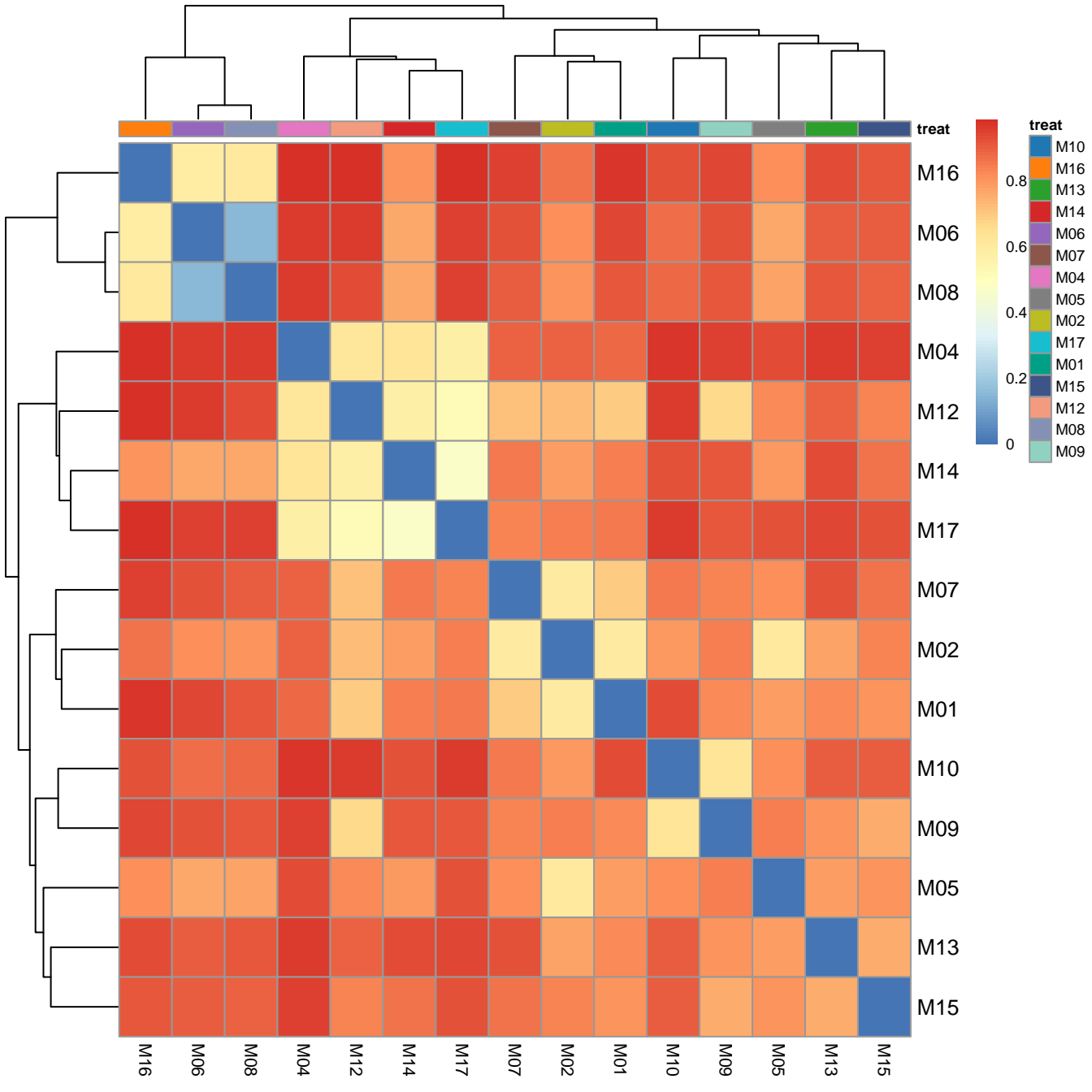

Supplement: Supplementary file 1 — customer_backup. [file MBO3-14-e70178-s001.zip › customer_backup/customer_backup/beta_diversity/sample_heatmap/treat/treat.bray_curtis_dm.heatmap.pdf]

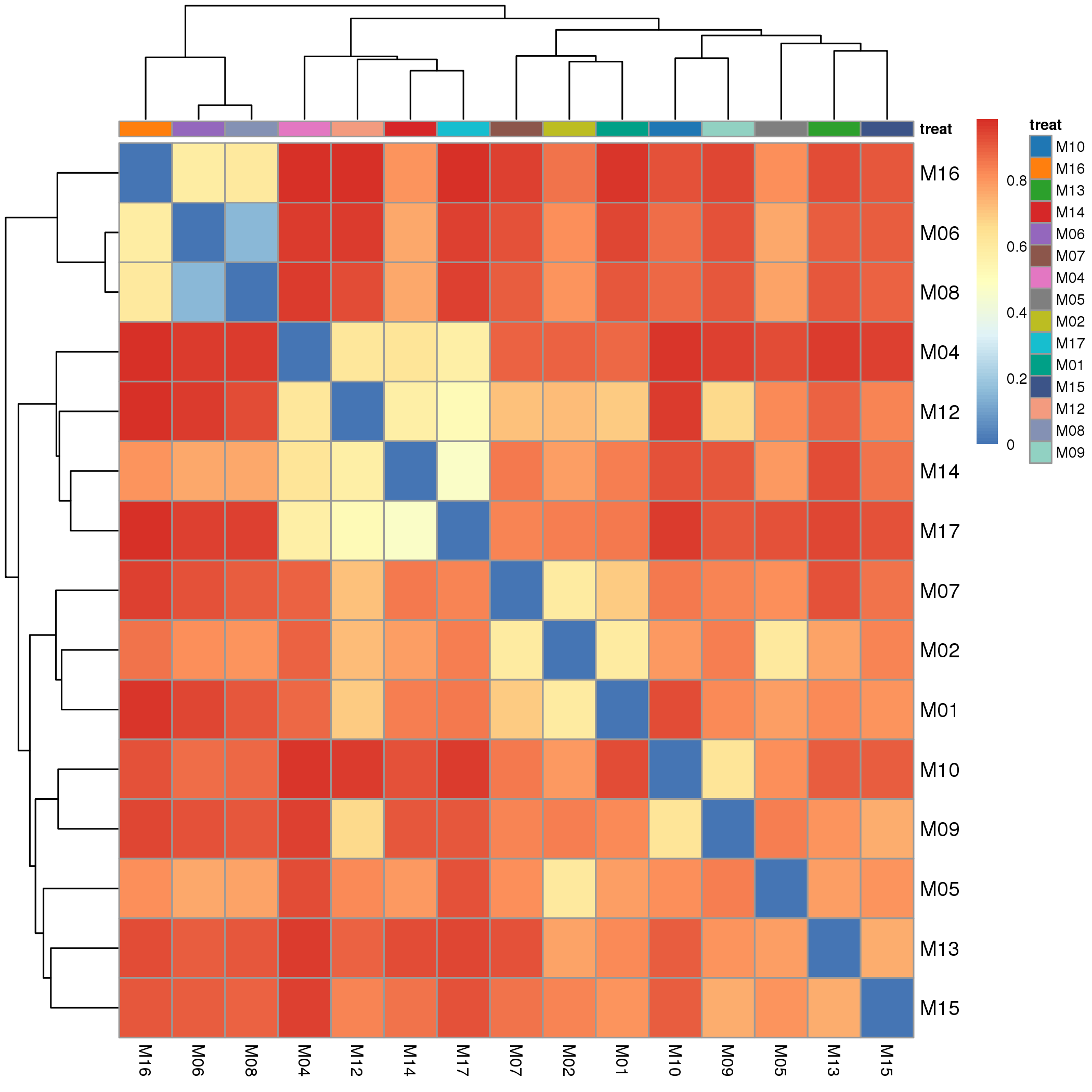

Supplement: Supplementary file 1 — customer_backup. [file MBO3-14-e70178-s001.zip › customer_backup/customer_backup/beta_diversity/sample_heatmap/treat/treat.bray_curtis_dm.heatmap.png]

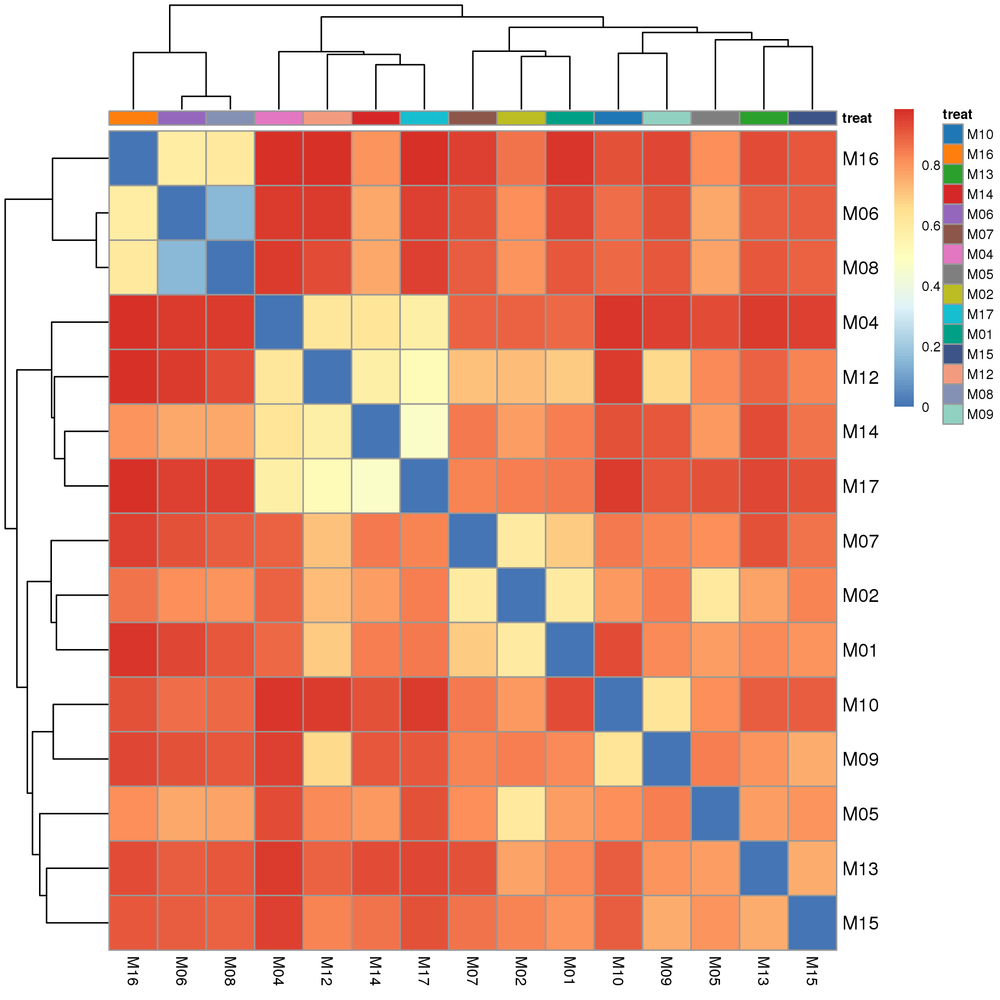

Supplement: Supplementary file 1 — customer_backup. [file MBO3-14-e70178-s001.zip › customer_backup/customer_backup/beta_diversity/sample_heatmap/treat/treat.bray_curtis_dm.heatmap_small.png]

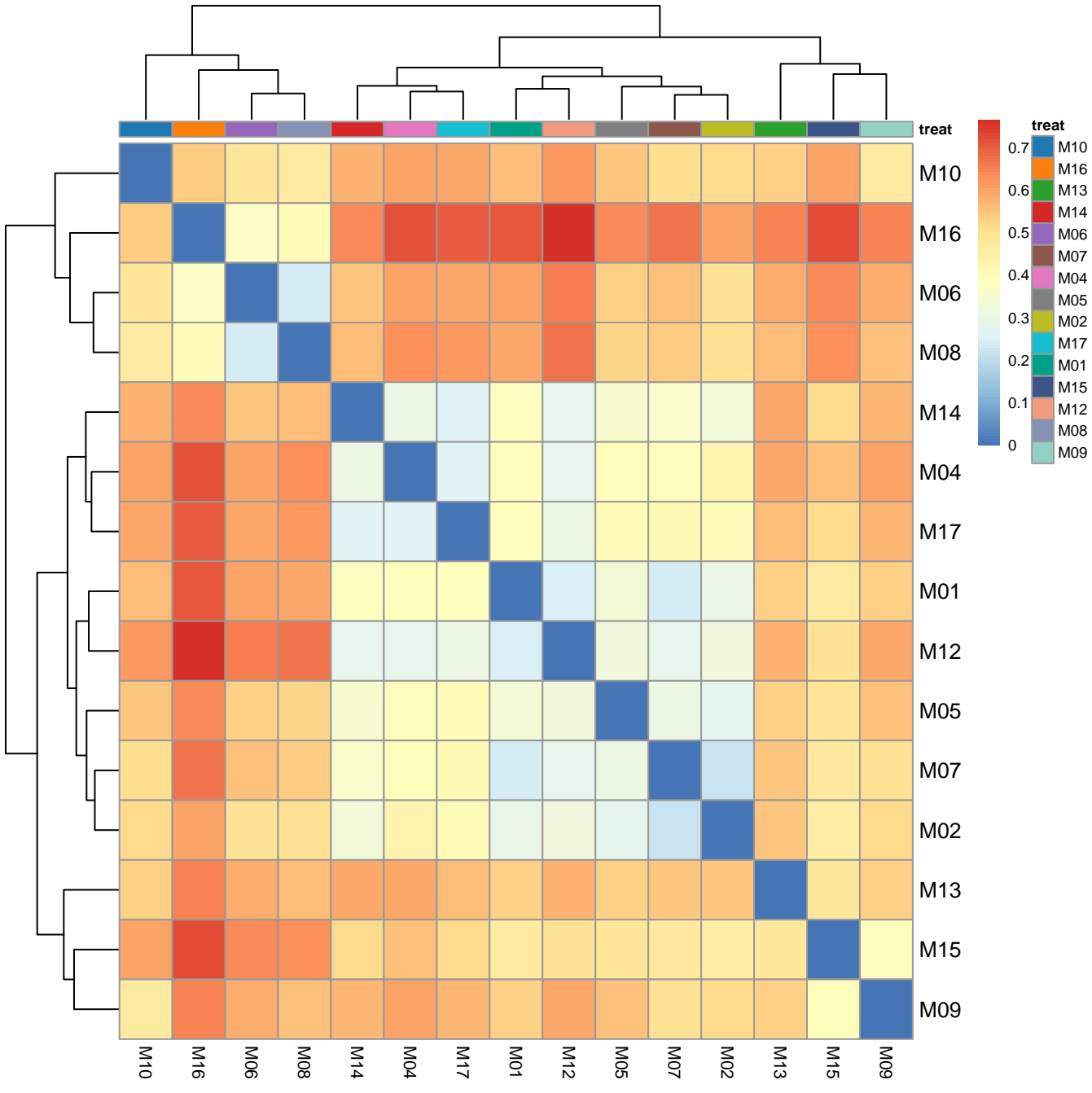

Supplement: Supplementary file 1 — customer_backup. [file MBO3-14-e70178-s001.zip › customer_backup/customer_backup/beta_diversity/sample_heatmap/treat/treat.unweighted_unifrac_dm.heatmap.pdf]

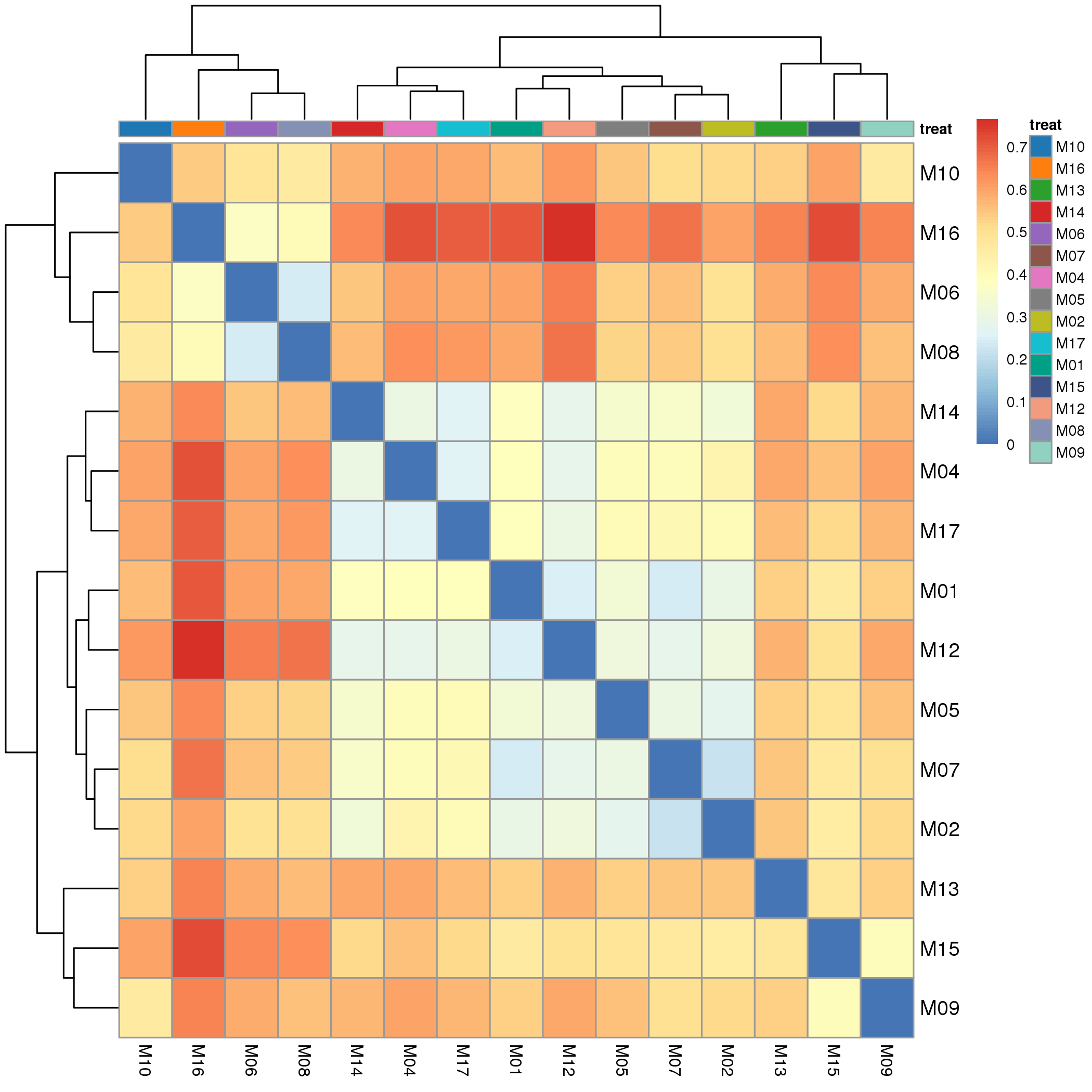

Supplement: Supplementary file 1 — customer_backup. [file MBO3-14-e70178-s001.zip › customer_backup/customer_backup/beta_diversity/sample_heatmap/treat/treat.unweighted_unifrac_dm.heatmap.png]

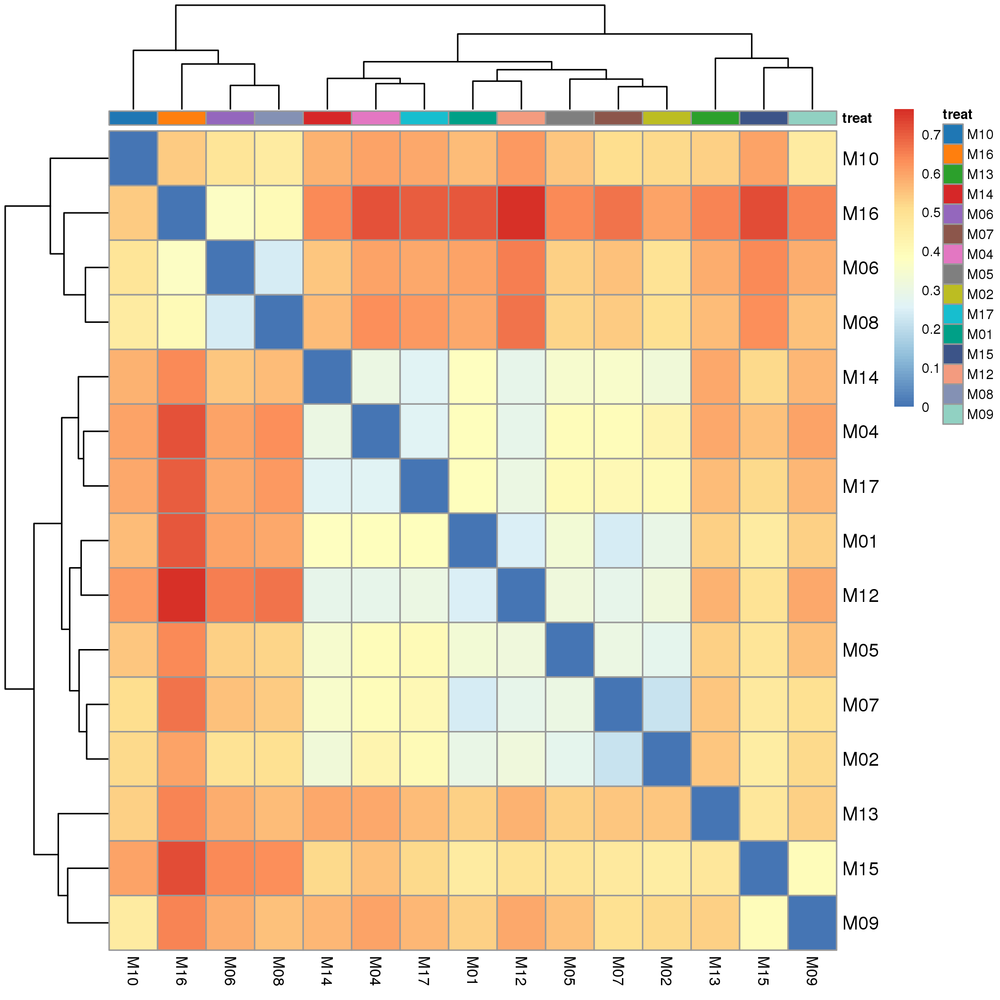

Supplement: Supplementary file 1 — customer_backup. [file MBO3-14-e70178-s001.zip › customer_backup/customer_backup/beta_diversity/sample_heatmap/treat/treat.unweighted_unifrac_dm.heatmap_small.png]

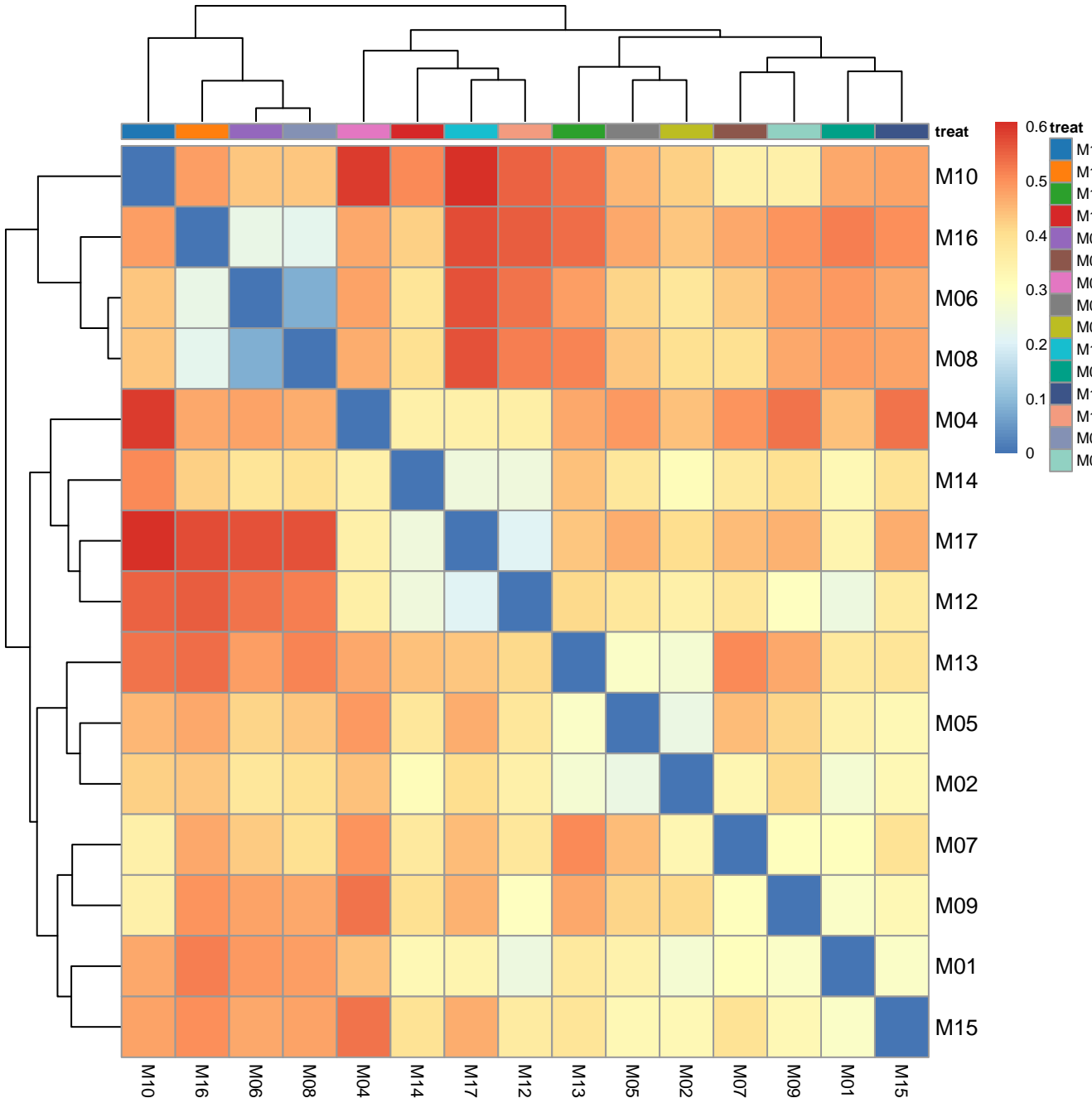

Supplement: Supplementary file 1 — customer_backup. [file MBO3-14-e70178-s001.zip › customer_backup/customer_backup/beta_diversity/sample_heatmap/treat/treat.weighted_unifrac_dm.heatmap.pdf]

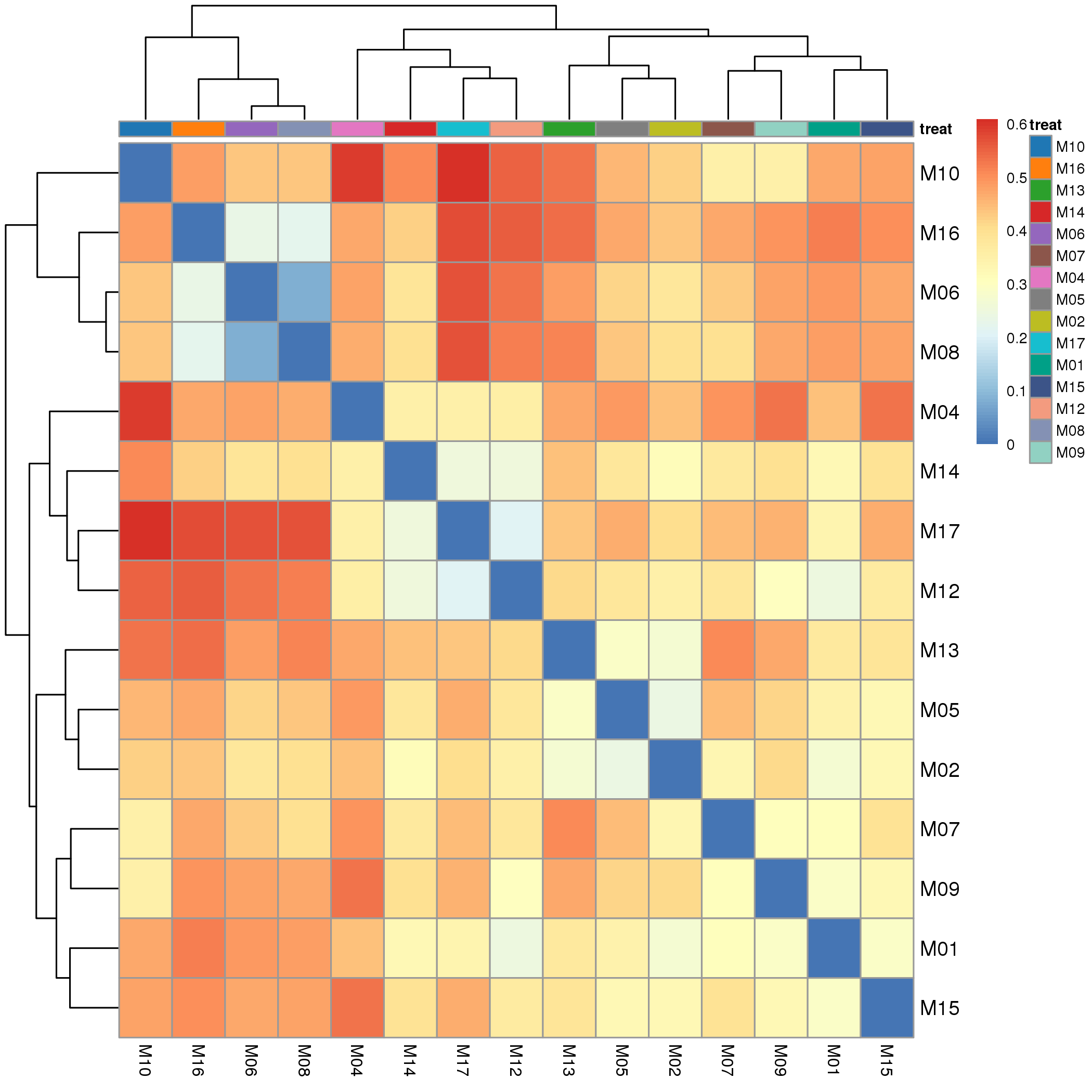

Supplement: Supplementary file 1 — customer_backup. [file MBO3-14-e70178-s001.zip › customer_backup/customer_backup/beta_diversity/sample_heatmap/treat/treat.weighted_unifrac_dm.heatmap.png]

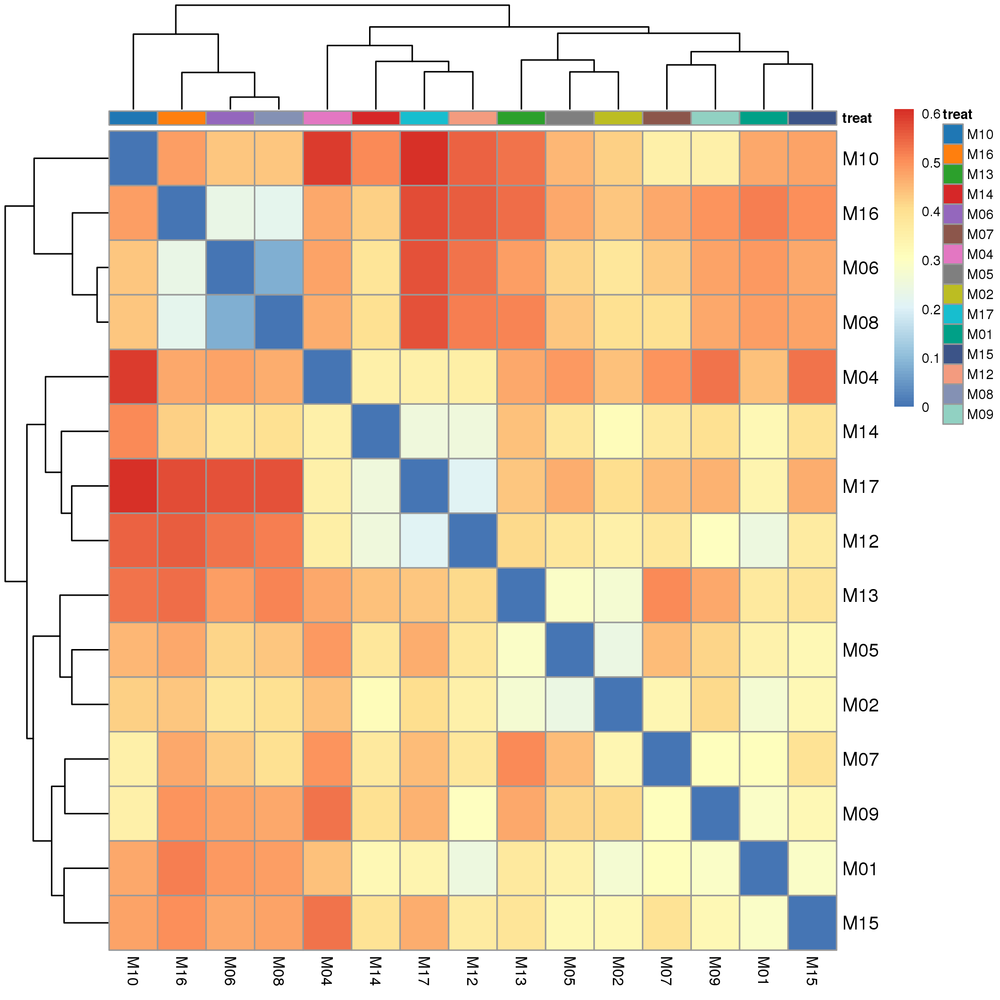

Supplement: Supplementary file 1 — customer_backup. [file MBO3-14-e70178-s001.zip › customer_backup/customer_backup/beta_diversity/sample_heatmap/treat/treat.weighted_unifrac_dm.heatmap_small.png]

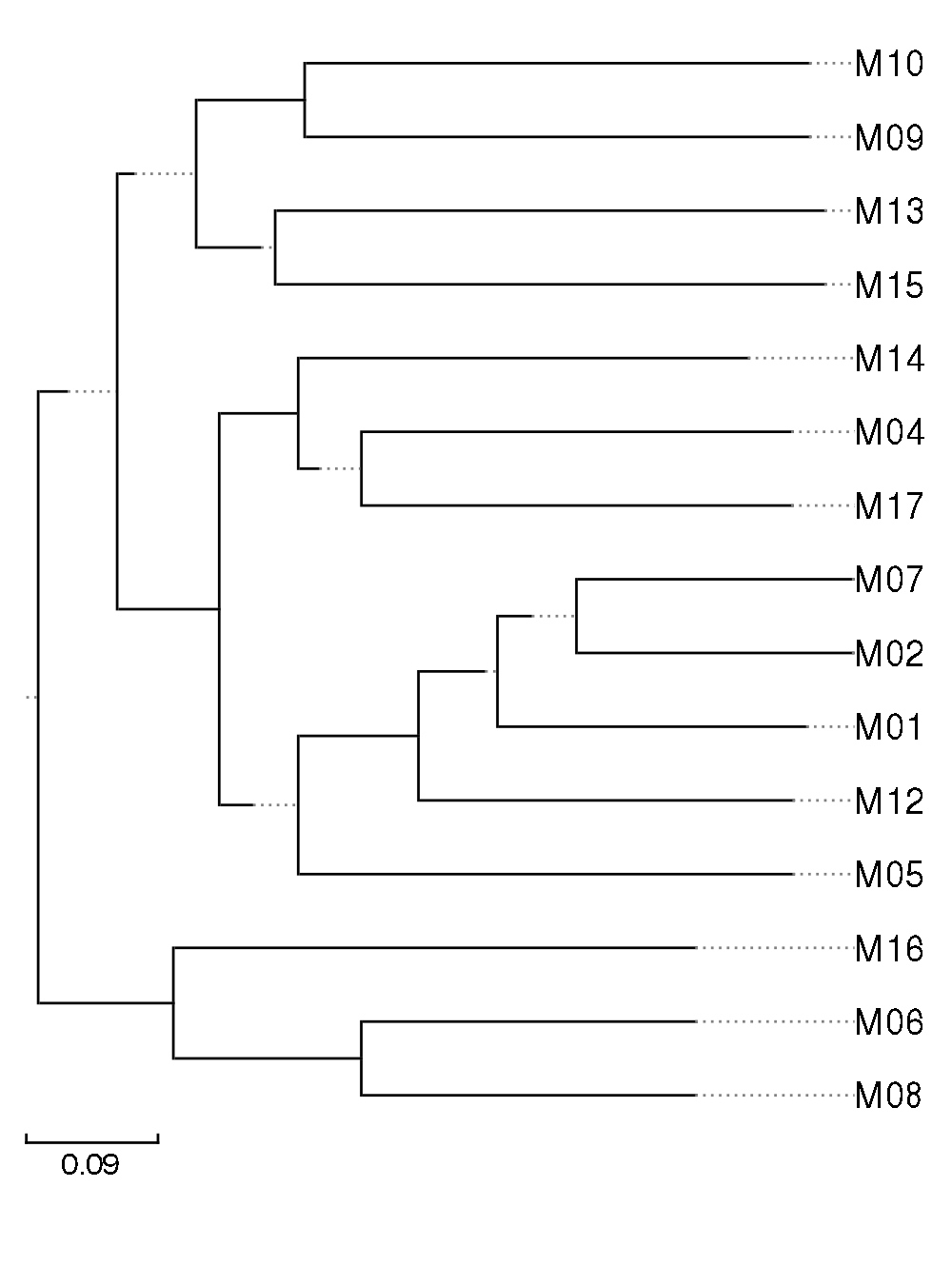

Supplement: Supplementary file 1 — customer_backup. [file MBO3-14-e70178-s001.zip › customer_backup/customer_backup/beta_diversity/upgma_tree/treat/treat.binary_jaccard.png]

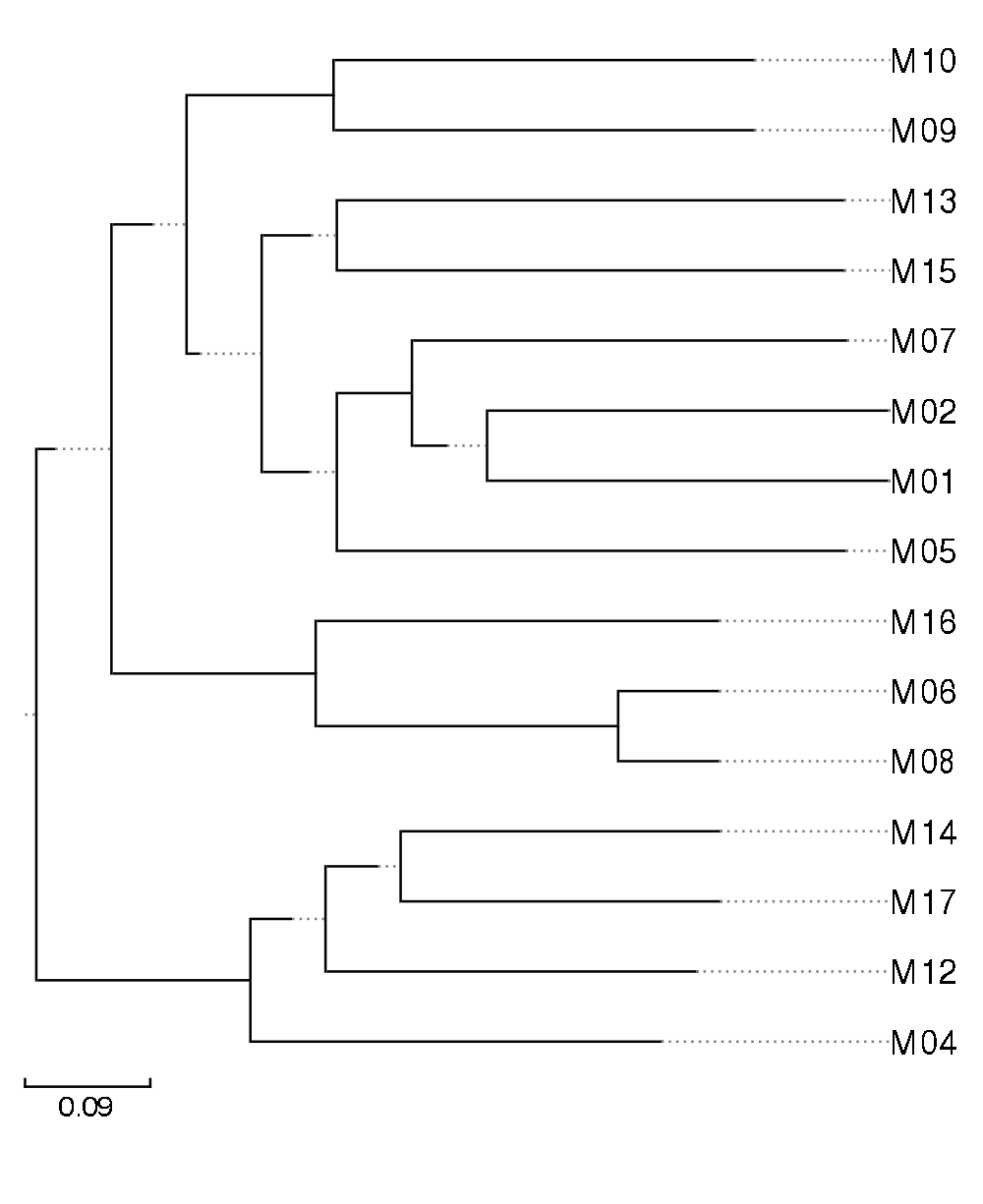

Supplement: Supplementary file 1 — customer_backup. [file MBO3-14-e70178-s001.zip › customer_backup/customer_backup/beta_diversity/upgma_tree/treat/treat.bray_curtis.png]

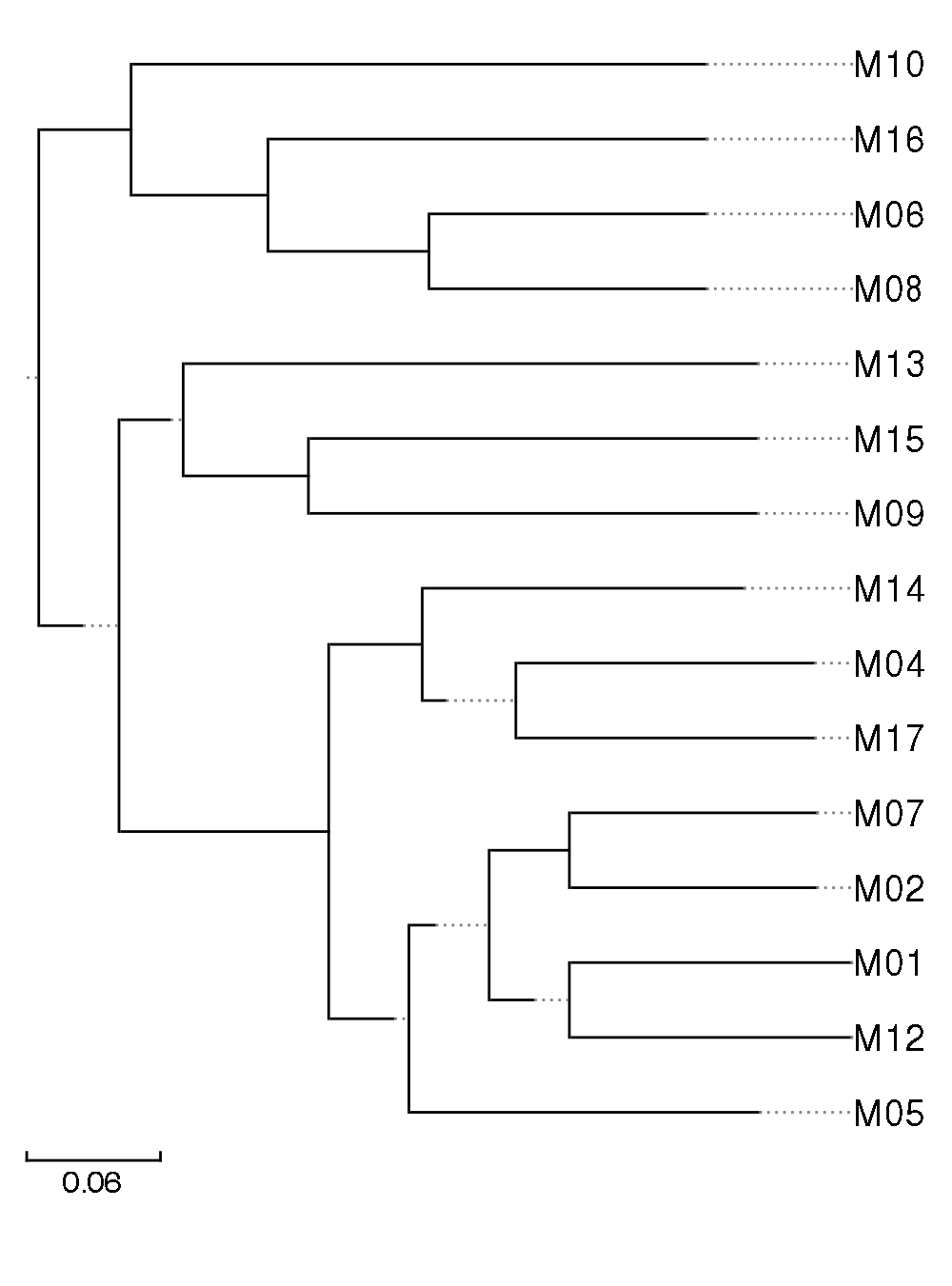

Supplement: Supplementary file 1 — customer_backup. [file MBO3-14-e70178-s001.zip › customer_backup/customer_backup/beta_diversity/upgma_tree/treat/treat.unweighted_unifrac.png]

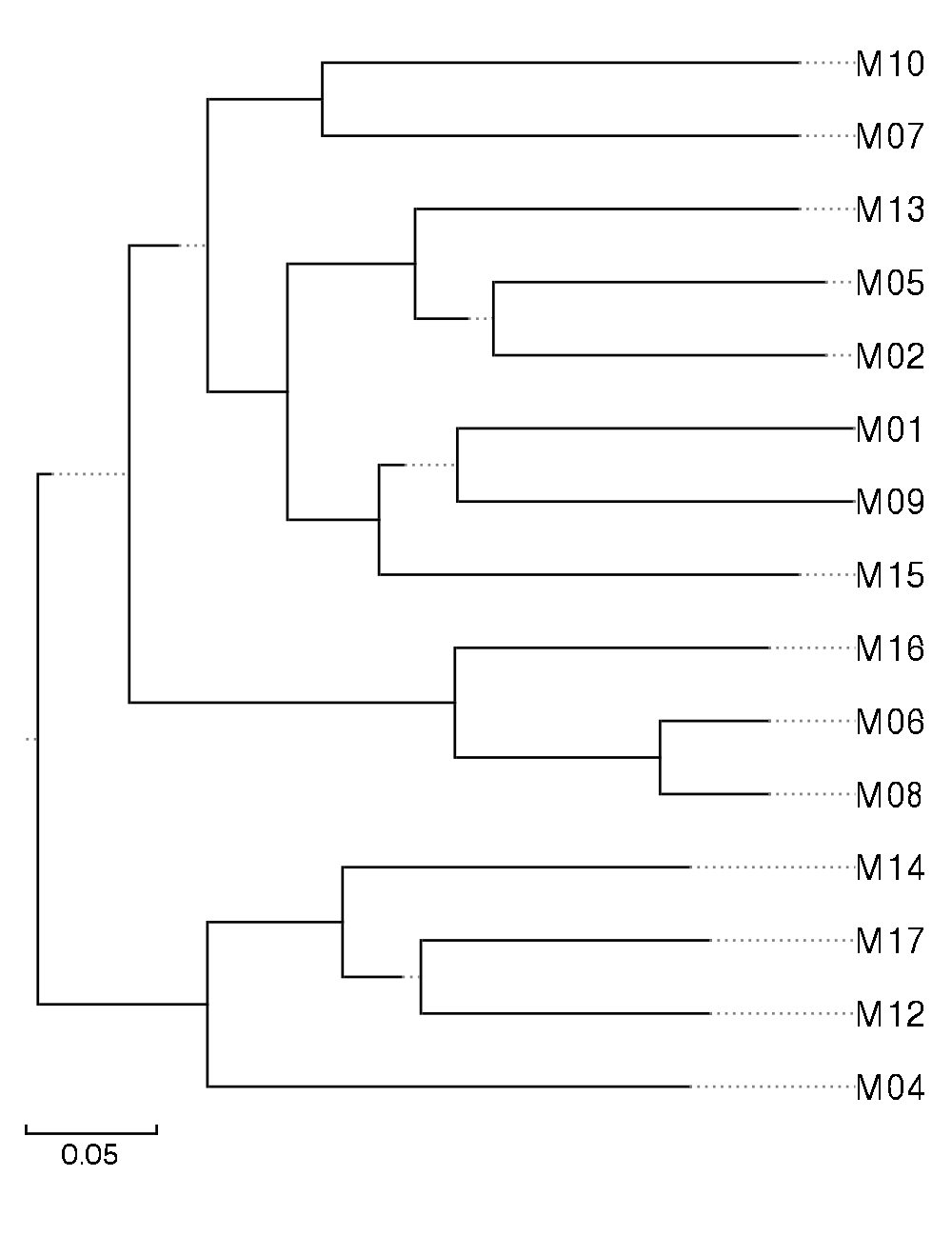

Supplement: Supplementary file 1 — customer_backup. [file MBO3-14-e70178-s001.zip › customer_backup/customer_backup/beta_diversity/upgma_tree/treat/treat.weighted_unifrac.png]

# Length Distribution

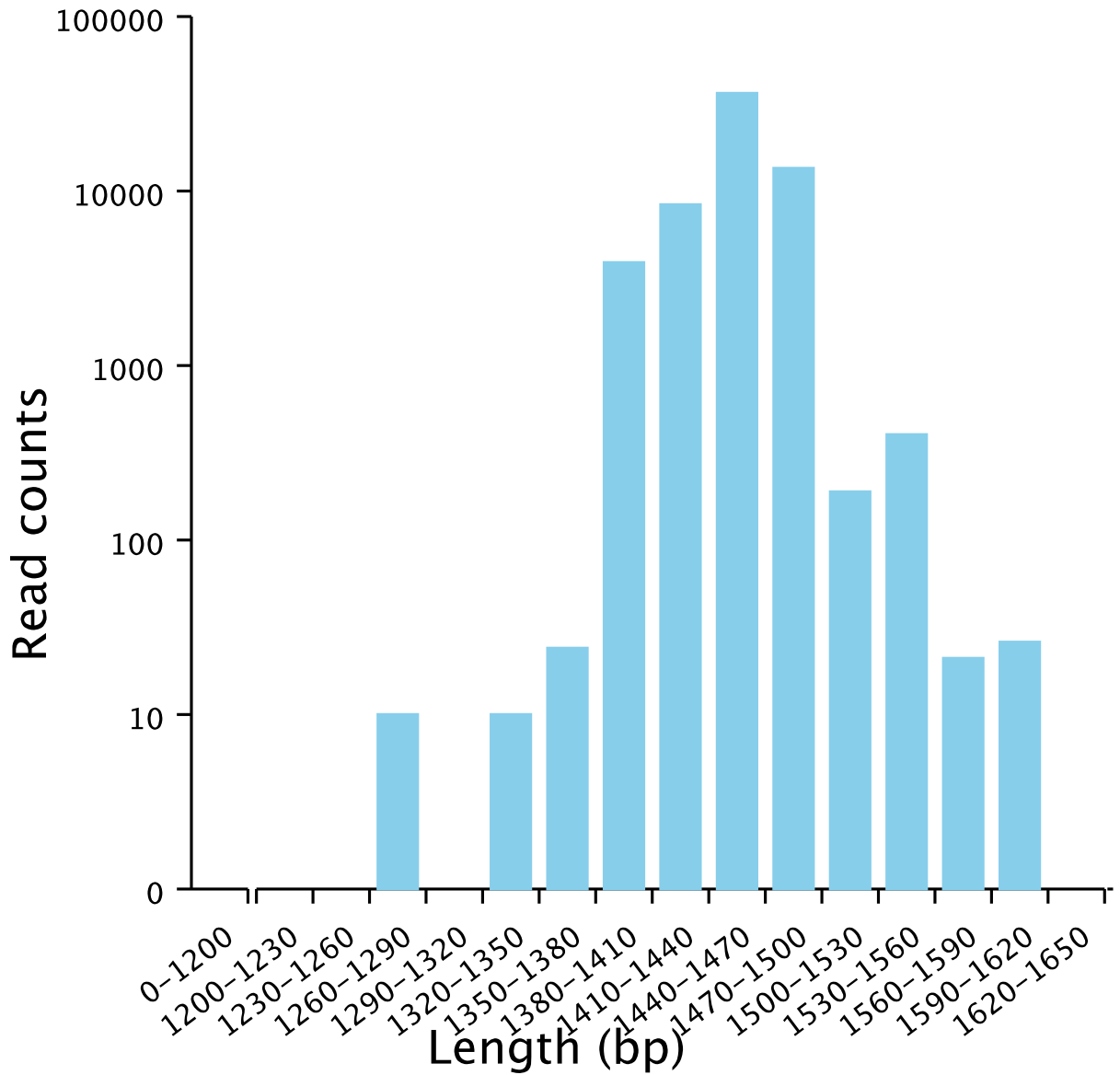

Supplement: Supplementary file 1 — customer_backup. [file MBO3-14-e70178-s001.zip › customer_backup/customer_backup/data_assement/M01_reads_length.pdf]
